# Analysis of total microcystins and nodularins by oxidative cleavage of their ADMAdda, DMAdda, and Adda moieties

**DOI:** 10.1016/j.acax.2020.100060

**Published:** 2020-09-02

**Authors:** Amanda J. Foss, Christopher O. Miles, Alistair L. Wilkins, Frode Rise, Kristian W. Trovik, Kamil Cieslik, Mark T. Aubel

**Affiliations:** a GreenWater Laboratories/CyanoLab, 205 Zeagler Drive, Palatka, FL, 32177, USA; b Measurement Science and Standards, National Research Council, 1411 Oxford Street, Halifax, NS, B3H 3Z1, Canada; c Norwegian Veterinary Institute, P. O. Box 750, Sentrum, N-0106, Oslo, Norway; d Chemistry Department, University of Waikato, Private Bag 3105, 3240, Hamilton, New Zealand; e Department of Chemistry, University of Oslo, P.O. Box 1033, N-0315, Oslo, Norway

**Keywords:** Microcystin, Nodularin, MMPB, Adda, ADMAdda, DMAdda, Adda, 3*S*-amino-9*S*-methoxy-2*S*,6,8*S*-trimethyl-10-phenyldeca-4*E*,6*E*-dienoic acid, ADMAdda, 3*S*-amino-9*S*-acetyloxy-2*S*,6,8*S*-trimethyl-10-phenyldeca-4*E*,6*E*-dienoic acid, DMAdda, 3*S*-amino-9*S*-hydroxy-2*S*,6,8*S*-trimethyl-10-phenyldeca-4*E*,6*E*-dienoic acid, MAPB, 2*R*-methyl-3*S*-acetyloxy-4-phenylbutanoic acid, Microcystin, MC, MHPB, 2*R*-methyl-3*S*-hydroxy-4-phenylbutanoic acid, MMPB, 2*R*-methyl-3*S*-methoxy-4-phenylbutanoic acid, MOMAPH, 2-methyl-3-oxo-4*R*-methyl-5*S*-acetyloxy-6-phenylhexanoic acid, MOMHPH, 2-methyl-3-oxo-4*R*-methyl-5*S*-hydroxy-6-phenylhexanoic acid, MOMMPH, 2-methyl-3-oxo-4*R*-methyl-5*S*-methoxy-6-phenylhexanoic acid, NOD, Nodularin

## Abstract

Microcystins (MCs) and nodularins (NODs) exhibit high structural variability, including modifications of the Adda (3*S*-amino-9*S*-methoxy-2*S*,6,8*S*-trimethyl-10-phenyldeca-4*E*,6*E*-dienoic acid) moiety. Variations include 9-*O*-desmethylAdda (DMAdda) and 9-*O*-acetylDMAdda (ADMAdda) which, unless targeted, may go undetected. Therefore, reference standards were prepared of [ADMAdda^5^]MCs and [DMAdda^5^]MCs, which were analyzed using multiple approaches. The cross-reactivities of the [DMAdda^5^]- and [ADMAdda^5^]MC standards were similar to that of MC-LR when analyzed with a protein phosphatase 2A (PP2A) inhibition assay, but were <0.25% when analyzed with an Adda enzyme-linked immunosorbent assay (ELISA). Oxidative cleavage experiments identified compounds that could be used in the analysis of total MCs/NODs in a similar fashion to the 2*R*-methyl-3*S*-methoxy-4-phenylbutanoic acid (MMPB) technique. Products from oxidative cleavage of both the 4,5- and 6,7-ene of Adda, DMAdda and ADMAdda were observed, and three oxidation products, one from each Adda variant, were chosen for analysis and applied to three field samples and a *Nostoc* culture. Results from the oxidative cleavage method for total Adda, DMAdda, and ADMAdda were similar to those from the Adda-ELISA, PP2A inhibition, and LC-MS/MS analyses, except for the *Nostoc* culture where the Adda-ELISA greatly underestimated microcystin levels. This oxidative cleavage method can be used for routine analysis of field samples and to assess the presence of the rarely reported, but toxic, DMAdda/ADMAdda-containing MCs and NODs.

## Highlights

• Toxic ADMAdda/DMAdda microcystins (MCs) exhibit low cross-reactivity with Adda ELISA.
• A method for oxidative cleavage was expanded to test for ADMAdda/DMAdda MCs.
• Two sites of oxidative cleavage of the Adda/DMAdda/ADMAdda were observed.
• Oxidation products were identified and analytically tested to quantitate ‘total MCs’.

## Graphical abstract

**Figure:**
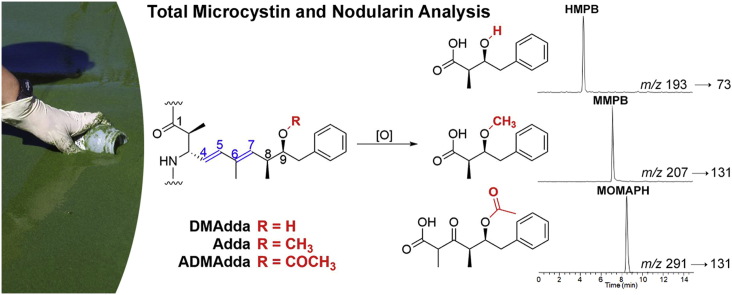

## 1 Introduction

**Fig. 1:**
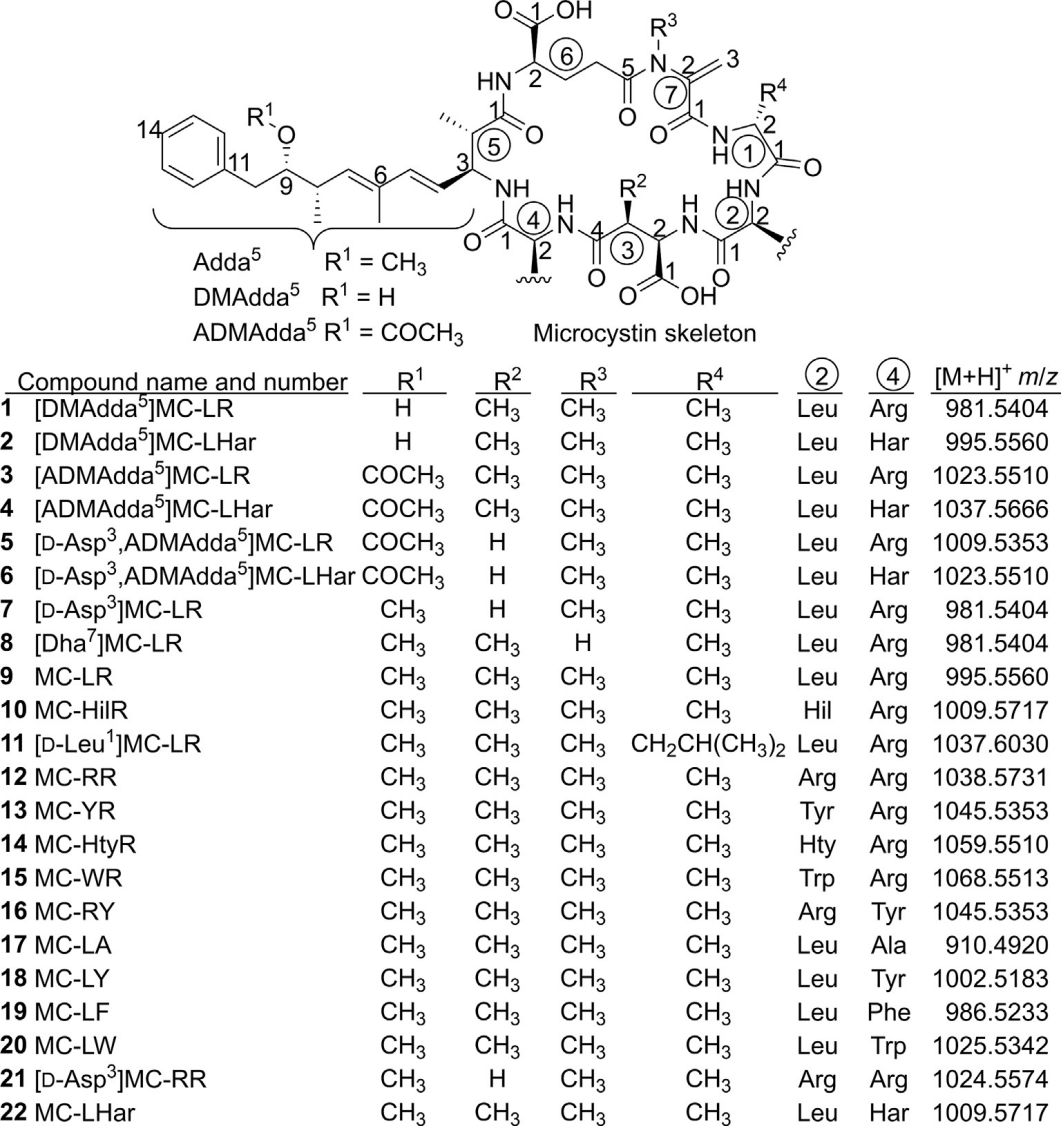
Structures of MCs and the exact masses for their mono-protonated ions are indicated. [DMAdda^5^]MCs (**1**, **2**) and [ADMAdda^5^]MCs (**3**, **4**, **5**, **6**) were isolated in this study. Amino acid residue-numbers are shown in the circles, while atom numbering for each residue is shown in plain text starting from the carboxyl carbon.

Microcystins (MCs) and nodularins (NODs) are cyanobacterially-produced hepatotoxins, with most sharing the Adda (3*S*-amino-9*S*-methoxy-2*S*,6,8*S*-trimethyl-10-phenyldeca-4*E*,6*E*-dienoic acid) moiety [1] at position-5 in the heptapeptide MCs and at position-3 in the pentapeptide NODs. Although Adda is conserved in the majority of MC and NOD variants, modifications have been observed. The most common of these occur at C-9 (Fig. 1) [2], with substitution of the methoxy group with a hydroxy or acetyloxy group [[3], [4], [5]]. These substitutions are designated 9-*O*-desmethylAdda (DMAdda) and 9-*O*-acetylDMAdda (ADMAdda), respectively. Of the over 250 MC and 10 NOD variants described, approximately 20% have substitutions within the Adda moiety [2,6].

While MCs containing these variants are not often reported, the presence of [ADMAdda^5^]MCs in the benthos [7,8] and in symbionts [9,10] has been described and attributed to the cyanobacterial genus *Nostoc*. [ADMAdda^5^]MCs have even been reported in benthic mats from the arctic [8]. Planktonic species of cyanobacteria reported to produce MCs with modified Adda include [ADMAdda^5^]MCs by *Planktothrix* [11], [DMAdda^5^]MCs by *Microcystis* [4], and [DMAdda^3^]NODs by *Nodularia* [12,13]. The biosynthesis of [DMAdda^5^]- and [ADMAdda^5^]MCs has a genetic basis, where portions of the microcystin synthetase gene cluster encode tailoring enzymes responsible for *O*-methylation (McyJ) [14,15] and *O*-acetylation (McyL) of Adda [16]. Therefore, strains lacking McyJ and possessing McyL, such as *Nostoc* sp. strain 152, nearly exclusively produce [ADMAdda^5^]MCs [16]. However, it is unknown how many other MC-producing cyanobacteria lack the gene encoding McyJ and possess the gene encoding for McyL, such as the *Planktothrix* strain reported to produce these unusual variants [11]. MCs with such modifications to their Adda moieties have been shown to have similar hepatotoxicity to MC-LR [5,17], making it important to be able to screen for their presence.

One frequently employed method for the analysis of MCs and NODs is enzyme linked immunosorbent assay (ELISA). There are multiple ELISAs developed for the analysis of MCs/NODs, each exhibiting differential cross-reactivity to MC and NOD congeners depending on the antibody development approach used. For instance, ELISAs with antibodies raised against MC-LR have exhibited low-to-no cross-reactivity to non-arginine MCs [18], and to [ADMAdda^5^]MCs even when arginine is present [11]. An improvement to congener cross-reactivity was achieved through the development of an ELISA with antibodies raised against Adda-haptens [19]. The Adda-ELISA is commercially available and utilized in countries such as the USA to screen for MCs/NODs in ambient source and drinking water [20]. Results are directly actionable by utilities in some states, resulting in drinking water treatment plant and recreational beach closures [21]. The Adda-ELISA was chosen for monitoring as it is expected to react to MCs/NODs with approximately equal sensitivity, regardless of the remaining amino acid composition. However, although modifications to the Adda moiety could potentially alter the cross-reactivity, this has not been tested experimentally. One broadly specific ELISA developed using a multi-hapten approach, was shown to cross-react with crude extracts containing ADMAdda- and DMAdda-containing MCs, but this assay is not currently commercially available and the cross-reactivity was not measured quantitatively [22].

**Fig. 2:**
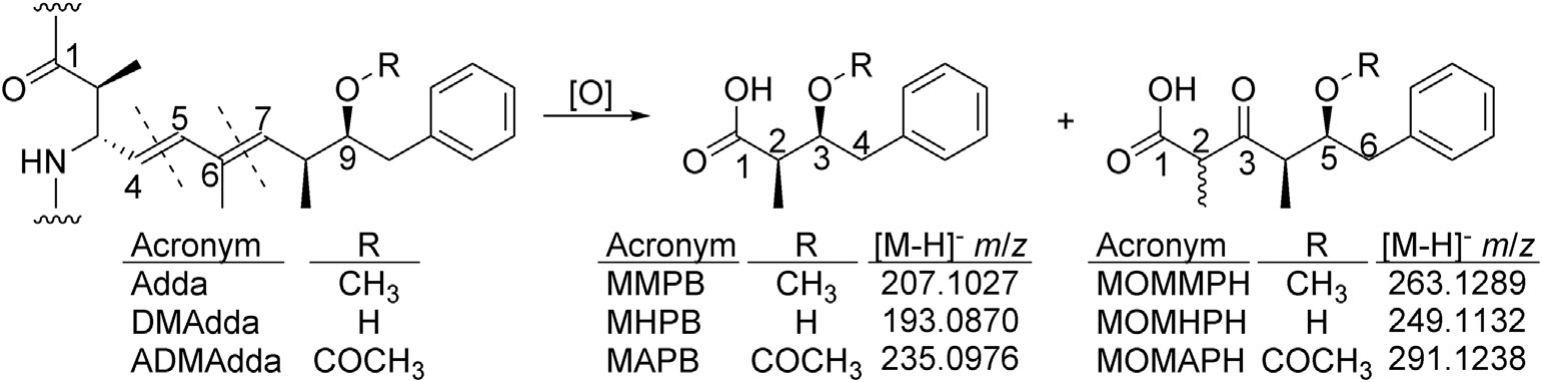
Oxidative cleavage of the Adda, DMAdda and ADMAdda moieties of MCs, showing the proposed structures of the cleavage products at the 4,5- and the 6,7-olefinic bonds (dashed lines). The compounds formed through oxidative cleavage of the 4,5-ene may undergo keto–enol tautomerisation leading to formation of stereoisomers. Note that atom-numbering for all compounds begins at the carboxyl carbon atoms, and is therefore different for the three groups of compounds.

The identification of ADMAdda and DMAdda variants is currently limited to congener-specific methods (e.g. LC-MS/MS). However, standards for instrument calibration are not available and, unless targeted, some congeners might remain undetected. In order to facilitate analysis of these modified-Adda-variants, the approach utilized for total Adda determination via oxidative cleavage of Adda to MMPB (2*R*-methyl-3*S*-methoxy-4-phenylbutanoic acid) could be applied. The originally-reported use of oxidation to cleave the Adda to measure total MCs was developed based on methodology for the analysis of unsaturated fatty acids [23,24]. The method preserves acyl ester bonds, while allowing for the quantitative determination of the oxidized products. Adda possesses olefinic bonds at C-4 and C-6, and oxidative cleavage of the 6,7-olefinic bond results in the formation of MMPB (Fig. 2). The MMPB approach has been used to quantitatively measure total Adda-containing MCs and NODs in water, benthic periphyton and animal tissues [[25], [26], [27], [28]]. However, to date, there have been no reports of using oxidative cleavage for analysis of MCs containing modified Adda moieties.

In this work, four [ADMAdda^5^]MC variants were extracted, purified and reference standards produced. Hydrolysis of [ADMAdda^5^]MCs produced standards of two [DMAdda^5^]MCs which were characterized by NMR spectroscopy and LC-MS. The standards were analyzed using a commercial PP2A inhibition assay and an Adda-ELISA to determine cross-reactivities. The MMPB method for oxidative cleavage and analysis of Adda-containing MCs/NODs was augmented to include ADMAdda- and DMAdda-containing MCs. This approach was applied to three field-collected samples and a culture of a *Nostoc* sp. to illustrate the potential of the method for monitoring for these rarely tested variants.

## 2 Methods

### 2.1 Materials and reagents

Deionized water (18 MΩ-cm) was generated with a PureLab Ultra (Evoqua Water Technologies, Jacksonville, FL, USA) or was HPLC grade (Thermo Fisher Scientific, Waltham, MA, USA). Reagents from Thermo Fisher Scientific included ACS grade KH_2_PO_4_, K_2_HPO_4_, (NH_4_)_2_CO_3_, NH_4_HCO_2_, trifluoroacetic acid (TFA; 98%), formic acid (≥98%), and HPLC grade (or better) ammonium acetate, acetic acid, methanol, hexanes and acetonitrile. Additional reagents from Millipore Sigma (St. Louis, MO, USA) included ACS grade Na_2_CO_3_, KMnO_4_, NaIO_4_ and NaHSO_3_. Solid-phase extraction (SPE) cartridges were Strata-X (60, 100, and 200 mg) and 200 mg Strata-X-AW (Phenomenex, Torrance, CA, USA), which were pre-conditioned using one column-volume of methanol and equilibrated with water (Strata-X) or 10 mM phosphate buffer at pH 7 (Strata-X-AW). A TurboVap-LV evaporator (Biotage, Charlotte, NC, USA) was used to evaporate solvents at 60 °C under a stream of N_2_. Centrifugation was conducted at 1500×*g*. Syringe filtration was conducted using 0.22 μm polyvinylidene fluoride (Millex-GV Filter, Millipore, Burlington, MA, USA).

Standards for calibration included: certified reference materials (CRMs) of NOD-R, MC-LR, MC-RR, and [Dha^7^]MC-LR from the National Research Council Canada (NRC) (Halifax, NS, Canada); a reference material (RM) of MC-RY [29] produced by the Norwegian Veterinary Institute (NVI) (Oslo, Norway) and the NRC; RMs of MC-WR, [d-Asp^3^]MC-RR, [d-Asp^3^]MC-LR, MC-HtyR, MC-LF, MC-LW, MC-HilR (purchased from Enzo Life Sciences, Farmingdale, NY, USA) and; RMs of MC-YR, MC-LA, MC-LY, and [d-Leu^1^]MC-LR produced by GreenWater Laboratories (Palatka, FL, USA). Internal standards included *d*_5_-MC-LF and *d*_7_-MC-LR from Eurofins Abraxis (Warminster, PA, USA).

### 2.2 Preparation of ADMAdda/DMAdda standards

#### 2.2.1 Purification of [ADMAdda^5^]MCs (**3**–**6**)

Lyophilized *Nostoc* sp. strain 152 (500 mg) from the University of Helsinki [5] was suspended in 75% MeOH containing 100 mM acetic acid (10 mL) and bath-sonicated for 25 min. The suspension was centrifuged, the supernatant retained, the pellet vortex-mixed with fresh extractant (5 mL), and centrifuged. The pooled supernatants were evaporated to near dryness, diluted (water; 10 mL), and applied to a Strata-X SPE column (200 mg). The column was washed (5% MeOH; 2 mL), eluted (90% CH_3_CN; 5 mL), and the eluate evaporated. The residue was dissolved (100 mM phosphate buffer, pH 7; 10 mL), washed with hexane (10 mL), and the aqueous layer applied to a weak anion exchange SPE (Strata-X-AW). The SPE was washed (5 mL of 25 mM ammonium acetate, followed by 20 mL MeOH), the MCs eluted (10 mL; 5% formic acid in MeOH), and the eluate evaporated.

The residue was dissolved (20% CH_3_CN; 2 mL) and purified by semi-preparative high-performance liquid chromatography (HPLC) using a Thermo Separations Product P4000 Pump, with a UV 2000 Detector set to 238 nm and its output converted to a digital signal using an SN 4000 Controller. Details of the linear gradient conditions for all semi-preparative HPLC methods are shown in Table S1. Initial separation (Method 1 in Table S1) was achieved using a Luna C18 column (5 μm, 150 × 10 mm, Phenomenex) and mobile phases A (0.01% TFA) and B (MeOH) at 2 mL min^−1^ Two major chromatographic peaks were collected (Fig. S1) eluting at 18.38 (**3** and **4**) and 19.47 (**5** and **6**) min. A portion of the first peak (18.38 min) was set aside for base hydrolysis (section 2.2.2), and the remainder was further separated by semi-preparative HPLC using the same column (Method 2 in Table S1) to give partial separation of **3** and **4** (Fig. S2). Final purification was achieved by semi-preparative HPLC on a NovaPak C18 column (4 μm, 4.6 × 250 mm, Waters Corporation, Milford, MA, USA) (Method 4 in Table S1, Fig. S3) to give [ADMAdda^5^]MC-LR (**3**) and [ADMAdda^5^]MC-LR (**4**) with >95% purity. The peak containing **5** and **6** from the initial semi-preparative HPLC step was further purified (Method 3, Table S1, Fig. S2) using the NovaPak column to afford [d-Asp^3^,ADMAdda^5^]MC-LR (**5**) and [d-Asp^3^,ADMAdda^5^]MC-LHar (**6**) with ≥95% purity. Final fractions containing purified **3**–**6** were evaporated and the residue dissolved in water (1 mL) for characterization.

#### 2.2.2 Purification of [DMAdda^5^]MCs (**1** and **2**)

An initial semi-preparative HPLC fraction (Fig. S1; peak at 18.38 min) from *Nostoc* sp. strain 152 contained a mixture of [ADMAdda^5^]MC-LR (**3**) and [ADMAdda^5^]MC-LHar (**4**) (ca 1 mg each; section 2.2.1) was treated with Na_2_CO_3_ (300 mM; 2 mL) for 3 d at ambient temperature. The products were purified as in section 2.2.1 by semi-preparative HPLC with the NovaPak column (Method 5, Table S1). Major UV-absorbing peaks (Fig. S4) were collected to afford [DMAdda^5^]MC-LR (**1**) and [DMAdda^5^]MC-LHar (**2**), the solvent was evaporated, and aliquots dissolved (water; 1 mL) for analysis.

#### 2.2.3 Purity and quantitation of [DMAdda^5^]MCs and [ADMAdda^5^]MCs

A Thermo Scientific Surveyor HPLC system coupled to a Surveyor photodiode array (PDA) detector and an LTQ XL Linear Ion Trap Mass Spectrometer were employed as previously described [27,30]. Briefly, analytical separations were achieved using a Kinetex C18 column (2.6 μm, 100 Å, 150 × 2.1 mm; Phenomenex) with mobile phases of water (A) and 95% CH_3_CN (B), both containing 2 mM formic acid and 3.6 mM ammonium formate. The gradient (0.2 mL min^−1^) was: A held at 70% for 10 min, 70–65% A over 8 min, held 65% A for 2 min, 65–30% A over 4 min, 30–70% A over 2 min, and held at 70% A for 4 min. Purity was assessed using HPLC–PDA (200–600 nm) of each standard at ca 10–20 μg mL^−1^. Quantitation was based on HPLC–UV (λ = 238 nm) peak areas relative to a CRM of MC-LR (**9**) as MCs purified in this work share identical UV chromophores to MC-LR. Identities were assigned through the comparison of LC–UV–MS^n^ data (retention time, spectra) to previous work [7]. MS/MS scans were conducted using 20% collision energy (CE) in positive ionization mode of [DMAdda^5^]MC-LR (**1**) at *m/z* 981.5, [DMAdda^5^]MC-LHar (**2**) at *m/z* 995.5, [d-Asp^3^,ADMAdda^5^]MC-LR (**5**) at *m/z* 1009.5, [ADMAdda^5^]MC-LR (**3**) and [d-Asp^3^,ADMAdda^5^]MC-LHar (**6**) at *m/z* 1023.5, and [ADMAdda^5^]MC-LHar (**4**) at *m/z* 1037.6. Aliquots of **1** and **2** (approximately 200 μg each) were dispensed into vials and the solvent evaporated for NMR spectroscopy. Remaining solutions were portioned into 10 μg aliquots, the solvent evaporated, and stored at −20 °C. A set of working stock solutions at 1.0 μg mL^−1^ in 10 mM phosphate buffer (pH 7) were maintained (−20 °C) for experiments.

#### 2.2.4 Nuclear magnetic resonance (NMR) spectroscopy of [DMAdda^5^]MCs (**1** and **2**)

^1^H and ^13^C NMR spectra were acquired from CD_3_OH solutions at 300 K using a Bruker AVIIIHD-800 spectrometer (Bruker BioSpin, Fallanden, Switzerland) equipped with a 5 mm TCI cryoprobe (^1^H, ^13^C, ^15^N) with automatic tuning and matching and Z-gradient accessories. ^1^H and 2D-COSY, TOCSY, DIPSI2 and ROESY spectra were obtained using in-house variants of standard Topspin pulse programmes with excitation sculpted (ES), continuous wave (CW), or combined ES and CW presaturation of the large CD_3_O*H* resonance at ca. 4.8 ppm applied on the F1 channel, and CW presaturation of residual C*H*D_2_OH resonance applied on the F2 channel. 1D-SELTOCSY and 1D-SELROESY NMR spectra were obtained with on-resonance F1 channel excitation of target signals and F2 channel CW presaturation of C*H*D_2_OH lines. Standard HSQC, HMBC, SHSQC and SHMBC spectra were acquired with CW presaturation of the CD_3_O*H* signal. ^1^H and ^13^C chemical shifts are reported relative to C*H*D_2_OH at 3.31 ppm and *C*D_3_OH at 49.3 ppm, respectively. Coupling constants are reported to ±0.1–0.2 Hz for CH_x_ signals or 0.3 Hz for NH signals.

### 2.3 Field sample extraction, targeted LC-MS/MS, and high-resolution MS analyses

Grab-samples of blooms collected from the West Coast (Lake Billy Chinook, OR; June 27, 2016), Midwest (private lake, IL; September 20, 2017), and East Coast (Poplar Island, Chesapeake Bay, MD; August 27, 2012; detailed analysis reported elsewhere [27]) of the USA were screened for cyanobacterial dominance. Wet mounts were scanned using a Nikon TE200 inverted microscope equipped with phase-contrast optics at up to 400 × . Samples (200–500 mL) were lyophilized, dried cells extracted, and fractionated by SPE as described above for the *Nostoc* sp. strain 152. The resultant eluates were evaporated, the residues reconstituted in 10% CH_3_CN (approx. 800 mg biomass mL^−1^), and further diluted (water) for analysis.

Intact MCs were quantitated as diluted aliquots in water (biomass concentrations of 0.01–1 mg mL^−1^) with internal standards added (*d*_7_-MC-LR and *d*_5_-MC-LF), and analyzed using targeted LC-MS/MS (section 2.6.2.2) for NOD-R and 21 MCs (Table S2). Standards used to calibrate the method included the six isolated in this work (**1**–**6**) and those listed in section 2.1 and Table S2. Transitions used to monitor the [ADMAdda^5^]MCs (**3**–**6**), [DMAdda^5^]MCs (**1** and **2**), and other MCs (**7**–**11**) sharing the same precursor ions are shown in Table 1. The remaining MRM transitions were as previously reported [31] (Table S2). Chromatographic procedures were as described in section 2.2.4. Xcalibur v 2.2 (Thermo Fisher Scientific, Waltham, MA, USA) was utilized for data processing via the internal standard method previously described [31].

**Table 1:** Pseudomolecular ion *m*/*z*, collision energy (CE), identity (ID), retention time (RT), and product ions (*m*/*z* with % relative intensity RI) for [ADMAdda^5^]MCs and [DMAdda^5^]MCs investigated in this study using targeted LC-MS/MS. Data from standards of [Adda^5^]MCs (**7** and **8**) sharing the same molecular weight as [DMAdda^5^]MC-LR (**1**) are also shown for reference as well as for MC-LR (**9**), MC-HilR (**10**) and [d-Leu^1^]MC-LR (**11**). Quantification ions are in bold text.

[M+H]^+^*m*/*z*	CID CE%	ID	Congener	RT (min)	Product ions *m*/*z* (RI)	ID	Congener	RT (min)	Product ions *m*/*z* (RI)
981.5	14%	**1**	[DMAdda^5^]MC-LR	3.92	539.5 (2%)	**7**	[d-Asp^3^]MC-LR	11.56	539.5 (25%)
					553.4 (25%)				553.4 (0%)
					**585.4** (100%)				585.4 (1%)
					599.3 (0%)				**599.3** (100%)
					852.6 (41%)				852.6 (25%)
					953.6 (54%)				953.6 (53%)
					963.6 (86%)				963.6 (70%)
						**8**	[Dha^7^]MC-LR	12.60	539.5 (28%)
									553.4 (0%)
									585.4 (0%)
									**599.3** (100%)
									852.6 (36%)
									953.6 (37%)
									963.6 (58%)
995.5	25%	**2**	[DMAdda^5^]MC-LHar	4.33	375.2 (27%)	**9**	MC-LR	11.23	375.2 (4%)
					553.5 (0%)				553.5 (28%)
					**599.4** (100%)				**599.4** (100%)
					866.5 (24%)				866.5 (41%)
					875.6 (27%)				875.6 (0%)
					967.6 (35%)				967.6 (62%)
					977.6 (57%)				977.6 (90%)
1023.6	18%	**3**	[ADMAdda^5^]MC-LR	12.64	553.4 (21%)	**6**	[d-Asp^3^,ADMAdda^5^]MC-LHar	15.15	553.4 (12%)
					**627.4** (100%)				627.4 (0%)
					641.4 (0%)				**641.4** (100%)
					738.5 (26%)				738.5 (15%)
					894.6 (35%)				894.6 (10%)
					963.6 (39%)				963.6 (31%)
					995.6 (75%)				995.6 (51%)
					1005.6 (70%)				1005.6 (44%)
1009.5	20%	**5**	[d-Asp^3^,ADMAdda^5^]MC-LR	13.25	567.4 (3%)	**10**	MC-HilR	15.13	567.4 (29%)
					599.5 (14%)				**599.5** (100%)
					**627.4** (100%)				627.4 (0%)
					981.5 (71%)				981.5 (57%)
					992.6 (67%)				992.6 (54%)
1037.6	25%	**4**	[ADMAdda^5^]MC-LHar	15.04	599.5 (0%)	**11**	[d-Leu^1^]MC-LR	16.50	**599.5** (93%)
					613.4 (19%)				613.4 (0%)
					**641.4** (100%)				641.4 (0%)
					908.6 (24%)				908.6 (42%)
					977.5 (28%)				977.5 (0%)
					1009.5 (46%)				1009.5 (84%)
					1019.6 (59%)				1019.6 (100%)

The extract from the mid-west *Microcystis* bloom was analyzed by LC-HRMS/MS as previously described [32] except that the stepped collision energy used to acquire HRMS/MS spectra was 30 and 50 eV, with the same hardware and mobile phases as in section 2.6.4.

### 2.4 Adda-ELISA

Extracts of field samples were analyzed using an Adda-ELISA (Eurofins Abraxis, Warminster, PA, USA) loaded in duplicate as previously described [28]. Serial-dilutions were conducted using water (biomass concentrations 0.0001–0.1 mg mL^−1^) to achieve absorbances within range of the calibration curve (0.15–4.0 ng mL^−1^). To assess cross-reactivities, 4-parameter logistic curves were constructed from dilutions of a CRM of MC-LR (0.2, 0.6, 1, 2.5, 4 ng mL^−1^) and compared to curves, generated independently, of the [DMAdda^5^]MCs and [ADMAdda^5^]MCs (50, 150, 250, 625 and 1000 ng mL^−1^). A SpectraMax M2 microplate reader coupled to a computer running SoftMax Pro 7 Software (Molecular Devices, Sunnyvale, CA) was utilized to obtain absorbances. GraphPad Prism 7.4 (San Diego, CA, USA) was used to calculate IC_50_ values.

### 2.5 PP2A inhibition assay

The field sample extracts used for ELISA analysis were also analyzed using a protein phosphatase 2A (PP2A) kit (Eurofins Abraxis) at the same sample concentrations, except for the *Nostoc* sp. strain 152 extract, which was diluted to fit the calibration curve. A CRM of MC-LR (**9**) and each isolated variant (**1**–**6**) were diluted in water (0.25, 0.5, 1, 2.5 ng mL^−1^) and analyzed in duplicate following the manufacturer’s directions (Fig. S5). The same plate reader used in ELISA analysis was utilized to obtain absorbances at 405 nm. SigmaPlot 12.5 (Systat Software Inc., San Jose, CA, USA) was used to calculate IC_50_ values.

### 2.6 Oxidative cleavage

#### 2.6.1 Oxidation procedure

Solutions of MC-LR (**9**), [DMAdda^5^]MC-LR (**1**) and [ADMAdda^5^]MC-LR (**3**) (10 μg mL^−1^) were oxidized for characterization of their oxidation products. Standard curves (in duplicate) were also prepared by oxidation of **1**, **3** and **9** at concentrations 1, 5, 10, 50, and 100 ng mL^−1^. The remaining variants isolated in this study (**2** and **4**–**6**) were assessed with the oxidative cleavage method as 4-point curves (5, 10, 50, and 100 ng mL^−1^). Each field sample extract was diluted in water (biomass concentrations 0.2–1 mg mL^−1^) to achieve analyte responses within the range of the standard curves. Each extract was oxidized in triplicate with spikes (n = 2). Spikes were prepared pre-oxidation using standards of MC-LR, [DMAdda^5^]MC-LR and [ADMAdda^5^]MC-LR at sufficient levels to double peak areas for quantitation (standard addition).

Solutions of 1 M K_2_CO_3_, 0.25 M KMnO_4_ and 0.25 M NaIO_4_ were prepared in water. The oxidant was premixed just prior to addition, with each reaction containing 100 μL K_2_CO_3_, 200 μL KMnO_4_, 200 μL NaIO_4_, and the sample (diluted to 500 μL with water), for a final reaction volume of 1 mL. Final reagent concentrations were 50 mM KMnO_4_, 50 mM NaIO_4_ and 100 mM K_2_CO_3_. After 1 h, 40% (w/v) NaHSO_3_ (75–125 μL) was added until solutions were clear. Solutions were applied to Strata-X SPE columns (60 mg), the column was washed (3 × 1 mL water), eluted (1 mL, 90% ACN), the eluate evaporated to dryness, the residue dissolved (1 mL, water), and syringe-filtered prior to analysis.

A time-course for oxidative cleavage of [DMAdda^5^]MC-LR (**1**), [ADMAdda^5^]MC-LR (**3**) and MC-LR (**9**) was conducted (in triplicate) to monitor the formation of targeted oxidation products at ambient temperature. A solution (500 μL) containing 250 ng of each standard in water was oxidized (addition of 500 μL oxidant, as above), and sub-sampled (150 μL, with addition of 50 μL 40% (w/v) NaHSO_3_) after 5, 15, 30, 60 and 120 min. The solutions were fractionated by SPE, the residue from the eluate dissolved in water (375 μL), and filtered, as described above.

#### 2.6.2 LC-MS analyses of oxidation products

##### 2.6.2.1 Ion trap LC-MS/MS and -MS/MS/MS

An LTQ XL Linear Ion Trap coupled with Surveyor MS Pump Plus (Thermo Scientific, Waltham, MS, USA) was used with sheath and auxiliary gas flow at 40 and 10 arbitrary units, respectively, capillary temperature 275 °C, isolation width 1.0, with source voltage at 3.5 kV (negative ionization) and 5 kV (positive ionization). Separations were achieved using a Kinetex F5 LC column (2.6 μm, 150 × 2.1 mm, Phenomenex) and mobile phases (A) water and (B) 95% CH_3_CN, both containing 2 mM formic acid and 3.6 mM ammonium formate. The gradient (0.2 mL min^−1^) was A 75–30% over 6 min, 30–75% A over 3 min, and held at 75% A for 2 min, with 20 μL full-loop injections.

Oxidized standards (10 μg mL^−1^) of MC-LR (**9**), [ADMAdda^5^]MC-LR (**3**) and [DMAdda^5^]MC-LR (**1**) were scanned in negative ionization mode. A list of target molecular ions was generated based on full-scan mass spectra. MS/MS spectra (negative ionization) were obtained using 20% CE for *m*/*z* 207 (MMPB), *m*/*z* 193 (2*R*-methyl-3*S*-hydroxy-4-phenylbutanoic acid (MHPB)) and *m*/*z* 291 (2-methyl-3-oxo-4*R*-methyl-5*S*-acetyloxy-6-phenylhexanoic acid (MOMAPH)). MS/MS/MS spectra were obtained for additional characterization of *m*/*z* 291 (MOMAPH) using 35% CE of the dominant product ion in the MS/MS spectrum (*m*/*z* 231). Positive ionization was also used for MS/MS characterization of *m*/*z* 293 (MOMAPH).

##### 2.6.2.2 Triple Quadrupole LC-MS/MS

A TSQ Quantum Access MAX Triple Quadrupole MS system equipped with a Heated Electrospray Ionization (HESI-II) Probe and Surveyor MS Pump Plus (Thermo Scientific) was employed with sheath and auxiliary gas flow at 45 and 15 arbitrary units, respectively; capillary temperature 275 °C; vaporizer temperature 300 °C (HESI-II); isolation width 1.0, and; spray voltage at 3.5 kV (negative ionization) or 4 kV (positive ionization). A Kinetex F5 LC column was used with mobile phases A (0.05% acetic acid in 5% methanol) and B (0.05% acetic acid in 95% methanol). The gradient (0.2 mL min^−1^) was 45–10% A over 10 min, 10–45% A over 2 min, and held 45% A for 2 min, with 20 μL full-loop injections. Negative ionization MS (*m*/*z* 50–300) and MS/MS spectra (10% CE) were obtained for *m*/*z* 235 (2*R*-methyl-3*S*-acetyloxy-4-phenylbutanoic acid (MAPB)), *m*/*z* 193 (MHPB), *m*/*z* 207 (MMPB), *m*/*z* 291 (MOMAPH), *m*/*z* 249 (2-methyl-3-oxo-4*R*-methyl-5*S*-hydroxy-6-phenylhexanoic acid (MOMHPH)) and *m*/*z* 263 (2-methyl-3-oxo-4*R*-methyl-5*S*-methoxy-6-phenylhexanoic acid (MOMMPH)). Positive ionization MS/MS spectra were also obtained for *m*/*z* 293 (MOMAPH) using 15% CE and compared to spectra from the ion trap experiments.

Routine analysis of samples included transitions for MMPB *m*/*z* 207 → **131**, and 175 (12% CE); MOMAPH *m*/*z* 291 → 231, **131**, 119, and 60 (15% CE), and; MHPB *m*/*z* 193 → 131, 119, and **73** (15% CE), with quantification ions in bold. Standard curves prepared from MC-LR (**9**), [DMAdda^5^]MC-LR (**1**) and [ADMAdda^5^]MC-LR (**3**) (0, 1, 5, 10, 50, and 100 ng mL^−1^) were analyzed and used to determine method detection limits (S/N = 3).

#### 2.6.3 Isolation of MOMAPH

Lyophilized *Nostoc* sp. strain 152 (75 mg) was vortex-mixed in 2.5 mL of oxidant (50 mM KMnO_4_, 50 mM NaIO_4_, 100 mM K_2_CO_3_), allowed to react for 1 h, and the reaction stopped by dropwise addition of 40% (w/v) NaHSO_3_ until the solution turned cloudy white. The sample was centrifuged and the supernatant retained. The pellet was resuspended (water; 1 mL), centrifuged, and the two supernatants pooled. The solution was applied to a Strata-X SPE column (200 mg), and the column was washed (water; 3 × 1 mL), eluted (90% CH_3_CN; 5 mL), and the eluate evaporated to dryness. The residue was dissolved (10 mM phosphate buffer, pH 7; 2 mL) and injected onto a NovaPak C18 (4 μm, 4.6 × 250 mm; Waters) column eluted isocratically with 35% CH_3_CN containing 2 mM formic acid and 3.6 mM ammonium formate (1 mL min^−1^) while monitoring the absorbance at 254 nm using the HPLC equipment described in Section 2.2.1. Fractions (1 mL each) were collected and analyzed by LC-MS/MS, which identified the compound as a single UV-absorbing peak eluting at ca 12 min. Fractions containing the compound were combined and the solvent evaporated.

#### 2.6.4 LC-high resolution MS of MOMAPH

LC–HRMS was conducted with a Q Exactive HF Orbitrap mass spectrometer equipped with a HESI-II probe (ThermoFisher Scientific, Waltham, MA, USA), an Agilent 1200 G1312B binary pump, a G1367C autosampler, and G1316B column oven (Agilent, Santa Clara, CA, USA). Analyses were performed with a 3.5 μm Symmetry Shield C18 column (100 × 2.1 mm; Waters, Milford, MA, USA) held at 40 °C with mobile phases A and B of H_2_O and CH_3_CN, respectively, each of which contained formic acid (0.1% v/v). A linear gradient (0.3 mL min^−1^) was used from 20 to 90% B over 18 min, then to 100% B over 0.1 min, followed by a hold at 100% B (2.9 min), then returned to 20% B over 0.1 min with a hold at 20% B (3.9 min) to equilibrate the column. Injection volume was 5 μL. The mass spectrometer was calibrated from *m*/*z* 74–1622 and *m*/*z* 69–1780 in positive and negative ionization modes, respectively, the spray voltage was 3.7 kV, the capillary temperature was 350 °C, and the sheath and auxiliary gas flow rates were 25 and 8 units, respectively, with MS data acquired from 2 to 20 min. Mass spectral data were collected using full scan mode with alternating positive and negative scans with data collected from *m*/*z* 150–500 using the 60,000 resolution setting, an AGC target of 1 × 10^6^ and a max IT of 120 ms. Putative MOMAPH was further probed in a targeted manner in negative ionization mode using the PRM scan mode at *m*/*z* 291.1 with a ±0.5 *m*/*z* precursor isolation window, the 30,000 resolution setting, an AGC target of 1 × 10^6^ and a max IT of 100 ms, with a stepped collision energy of 15, 20 and 25 eV.

## 3 Results and discussion

### 3.1 Intact [DMAdda^5^]MCs and [ADMAdda^5^]MCs

#### 3.1.1 LC-MS/MS

Four [ADMAdda^5^]MCs (**3**–**6**) were purified from *Nostoc* sp. strain 152, two of which (**3** and **4**) were also hydrolysed to [DMAdda^5^]MCs (**1** and **2**) in this study (Fig. 1, Table 1). The purification of the [ADMAdda^5^]MCs and [DMAdda^5^]MCs required multiple semi-preparative HPLC steps to achieve final products with ≥95% purity. Compounds **1**–**6** showed LC-MS/MS retention times and spectra (Figs. S6–S7) consistent with their proposed identities (Fig. 1), which were previously extracted from *Nostoc* sp. strain 152 and confirmed by NMR spectroscopy (**1** and **3**–**6**) [5,7] or tentatively identified by LC–MS/MS (**2**) [33].

**Fig. 3:**
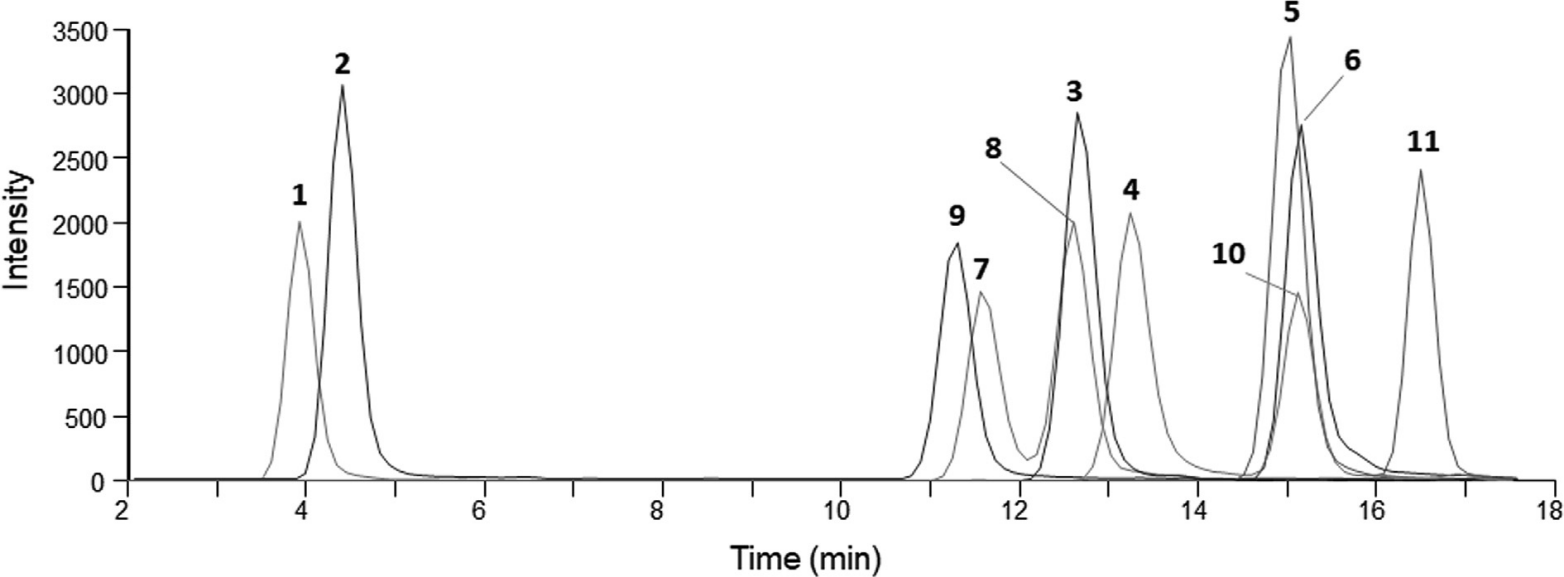
LC–MS/MS chromatogram illustrating the targeted intact [DMAdda^5^]MCs (**1**, **2**) and [ADMAdda^5^]MCs (**3**–**6**) purified in this work (50 ng mL^−1^), MC-LR (**9**), and other MCs (**7**, **8**, **10**, **11**) sharing pseudomolecular ions with **1**, **4** and **5**, using the quantification ions in Table 1.

Chromatographically, [DMAdda^5^]MCs **1** and **2** eluted far earlier than the Adda^5^-containing MC-LR (**9**), with [ADMAdda^5^]MCs **3** and **4** eluting just after **9**, followed by [d-Asp^3^,ADMAdda^5^]MC variants **5** and **6** (Fig. 3). The early elution of [DMAdda^5^]MCs presents a benefit as well as possible pitfall. While their chromatographic behaviour provides additional qualification for compound identification when reference standards are not available, care should be taken to ensure that the detectors (*e.g*. MS, UV) are acquiring data and that peaks of interest do not co-elute with non-retained matrix. MS/MS fragmentation patterns also allow for some differentiation, as relative fragment ion intensities varied between Adda-, DMAdda- and ADMAdda-containing MCs (Table 1). Diagnostic product ions for Arg^4^-containing ADMAdda- and DMAdda-MCs included *m*/*z* 627 and *m*/*z* 585, respectively, from [Arg^4^-ADMAdda/DMAdda^5^-Glu^6^ + H]^+^. When Har^4^ was present, diagnostic ions included *m*/*z* 641 for ADMAdda^5^-containing MCs (**4**, **6**), but the corresponding dominant fragment ion with *m*/*z* 599 for [DMAdda^5^]MC-LHar (**2**) was not unique as it was shared with Arg^4^-Adda^5^-containing MCs. The origin of the *m*/*z* 599 product ion from Arg^4^-containing ADMAdda-MCs has been reported as being [Arg^4^-ADMAdda^5^-Glu^6^ – CO + H]^+^ [9], which presents with the same *m*/*z* with [Arg^4^-Adda^5^-Glu^6^ + H]^+^. LC-MS/MS generated curves exhibited good linearity (All R^2^ ≥ 0.995) from 1 to 100 ng mL^−1^ for isolated standards (**1**–**6**) (Fig. S8). Stock solutions of each variant were monitored over the course of the study and were determined stable for at least one year stored between experiments at −20 °C.

#### 3.1.2 NMR spectroscopic analysis of [DMAdda^5^]MCs

The identities of the semisynthetic [DMAdda^5^]MCs **1** and **2** were confirmed through NMR spectroscopy (Table 2) because no authentic standards were available for these compounds, and no published NMR data was available for **2**. Detailed analyses of ^1^H, DEPT, DEPTQ, COSY, DIPSI2, HSQC and HMBC NMR spectra recorded from CD_3_OH with ES, CW or combined ES and CW presaturation of the large H_2_O/HOD line at ca. 4.8 ppm and CW presaturation of residual C*H*D_2_OH lines. This, supported by higher resolution SELTOCSY, SHSQC and SHMBC spectra, established that **1** was the 9-*O*-desmethylAdda analogue of MC-LR and that **2** was an analogue of **1** containing Har instead of Arg at position-4. The ^1^H and ^13^C NMR chemical shifts, and ^1^H^1^H coupling constants of **1** and **2**, where resolved, are reported in Table 2. These assignments can be compared with those reported for MC-LR (**9**) [3] and [ADMAdda^5^]MC-LHar (**4**) [3], and with ^1^H NMR data for MC-LHar (**22**) [34] and [DMAdda^5^]MC-LR (**1**) [4], in CD_3_OD.

**Table 2:** ^1^H and^13^C NMR assignments for [DMAdda^5^]MC-LR (**1**) and [DMAdda^5^]MC-LHar (**2**) in CD_3_OH^*a*^.

Residue	Atom	Type	[DMAdda^5^]MC-LR (1)			[DMAdda^5^]MC-LHar (2)		
			δ^13^C	δ^1^H	mult. *J* (Hz)	δ^13^C	δ^1^H	mult. *J* (Hz)
d-Ala^1^	1	C	175.6			175.5		
	2	CH	50.5	4.57	M	50.3	4.60	m
	2-NH			7.97	brd 7.7		7.93	brd 8.0
	3	CH_3_	17.5	1.36	brd 7.4	17.5	1.35	brd 7.4
Leu^2^	1	C	175.5			175.5		
	2	CH	56.0	4.28	ddd 10.8, 6.8, 3.6	55.6	4.28	ddd 10.8, 6.9, 3.6
	2-NH			8.30	brd 6.8		8.28	brd 6.9
	3	CH_2_	40.1	1.57	M	40.9	1.56	m
				2.04	M		2.08	m
	4	CH	26.1	1.78	M	26.1	1.78	m
	4-Me	CH_3_	23.9	0.89	d 6.7	23.9	0.90	d 6.7
	5	CH_3_	21.4	0.87	d 6.6	21.4	0.87	d 6.6
d-Masp^3^	1	C	177.0			176.8		
	2	CH	58.3	4.39	dd 9.5, 3.9	58.4	4.38	dd 9.4, 3.9
	2-NH			7.69	brd 9.5		7.80	brd 9.4
	3	CH	43.1	3.12	m	42.9	3.14	
	3-Me	CH_3_	15.6	1.03	d 7.0	15.7	1.03	d 7.1
	4	C	178.9			179.0		
Arg^4^	1	C	172.1			172.5		
	2-NH			8.55	brd 8.3		8.54	brd 8.6
	2	CH	53.0	4.33	m	53.1	4.35	m
	3	CH_2_	29.5	1.54	m	31.2	1.52	m
				2.02	m		2.02	m
Har^4^	3a	CH_2_				24.2	1.32	m
							1.37	m
	4	CH_2_	26.7	1.54	m	29.38^*c*^	1.54	m
	5	CH_2_	42.30^*b*^	3.14	m	42.4	3.13	m
	6	C	159.0			159.0		
Adda^5^	1	C	177.0			176.9		
	2	CH	45.2	3.11	m	45.3	3.10	m
	2-Me	CH_3_	16.1	1.05	d 6.9	16.1	1.05	d 6.9
	3	CH	57.2	4.57	m	57.1	4.58	m
	3-NH			8.16	brd 8.7		8.10	brd, 8.8
	4	CH	127.8	5.55	dd 15.5, 9.0	127.3	5.54	dd 15.5, 8.9
	5	CH	138.9	6.25	d 15.5	138.7	6.24	d 15.5
	6	C	134.2			134.1		
	6-Me	CH_3_	13.2	1.69	s	13.2	1.70	s
	7	CH	137.3	5.45	d 9.8	137.3	5.44	d 9.8
	8	CH	40.0	2.53	ddq 9.8, 6.7, 6.7	39.9	2.53	ddq 9.8, 6.7, 6.7
	8-Me	CH_3_	16.7	1.02	d 6.7	16.6	1.02	d 6.7
	9	CH	78.2	3.60	m	78.2	3.60	m
	10	CH_2_	42.96^*b*^	2.59	dd 13.9, 8.6	42.9	2.59	dd 14.0, 8.5
				2.82	dd 13.9, 4.2		2.82	dd 14.0, 4.2
	11	C	141.1			141.1		
	12/16	CH	130.6	7.19	d 7.5	130.6	7.19	d 7.5
	13/15	CH	129.4	7.24	t 7.5	129.4	7.24	t 7.5
	14	CH	127.2	7.15	t 7.3	127.2	7.15	t 7.2
d-Glu^6^	1	C	179.6			179.6		
	2	CH	56.9	4.10	∼q 7.5^*c*^	58.8	4.11	∼q 7.5^*c*^
	2-NH			8.33	brd 7.0		8.31	brd 7.0
	3	CH_2_	29.2	1.91	m	29.42^*c*^	1.91	m
				2.12	m		2.12	m
	4	CH_2_	33.8	2.57	m	33.8	2.55	m
				2.68	ddd 17.2, 12.5, 5.1		2.65	ddd 17.2, 12.3, 5.0
	5	C	177.4			177.5		
Mdha^7^	1	C	166.6			166.5		
	2	C	146.7			146.8		
	2-NMe	CH_3_	38.6	3.35	s	38.6	3.33	s
	3	CH_2_	114.2	5.41	s	114.2	5.41	s
				5.85	s		5.86	s

^*a*^mult., multiplicity; s, singlet, d, doublet; t, triplet; q, quartet; br, broad.^*b,c*^Pairs of ^13^C shifts resolved in SHSQC spectra.^*d*^Approximate quartet arising from 3 × ∼7.5 Hz d couplings.

The presence of a 9-OH group in the 9-*O*-desmethylAdda unit of **1**, as opposed to a 9-OCH_3_ group as in MC-LR, was revealed by the absence of ^1^H [4] and ^13^C NMR signals attributable to the presence of an Adda 9-OCH_3_ group, and by the occurrence of the Adda C-9 signal of **1** at 78.2 ppm and its H-9 signal at 3.60 ppm, respectively, rather than at 88.3 ppm and 3.27 ppm, respectively, as reported by Namikoshi et al. [3] for MC-LR (**9**).

The ^2^*J* and ^3^*J* couplings of the H-10_a_ (2.59 ppm) and H-10_b_ (2.82 ppm) signals of the desmethylAdda residue of **1** (dd, *J* = 13.9, 8.6 Hz, and dd, *J* = 13.9, 4.2 Hz, respectively), corresponded closely to those reported (to ± 0.5 Hz) for the equivalent protons of MC-LR [3]. These observations are consistent with the relative configuration of C-9 of the desmethylAdda of **1**, and the stereochemical disposition of H-9 relative to the H-10_a_ and H-10_b_ methylene protons, being the same as those of the equivalent protons of MC-LR (**9**). Similarly, the ^1^H NMR chemical shifts and coupling constants observed for the H-3 (4.57 ppm, m), H-4 (5.55 ppm, dd, 15.5, 9.0 Hz) and H-5 (6.25 ppm, d, 15.5 Hz) signals of the 9-*O*-desmethylAdda residue of **1** were essentially identical to those reported for the corresponding protons of MC-LR (**9**) [3]. Furthermore, all ^1^H chemical shift and coupling constant assignments for the Adda^5^ and Mdha^7^ moieties of **1** in CD_3_OH were also nearly identical to those reported by Namikoshi et al. [4] for **1** in CD_3_OD. The d-Glu^6^ H-4_b_ of **1**, which occurred at 2.68 ppm as a ddd (Table 2), included a large 17.2 Hz coupling attributable to a ^2^*J* coupling between d-Glu^6^ H-4_a_ and H-4_b_, which are located adjacent to a carbonyl group. d-Glu^6^ H-4_b_ also showed 12.5 Hz and 5.1 Hz ^3^*J* couplings to the neighbouring H-3_a_ and H-3_b_.

Correlations observed in COSY and in DIPSI2 experiments performed with mixing times of 80 and 160 ms verified that the foregoing proton signals assignments, and also those of protons associated with the other amino acid residue units of **1**, were as reported in Table 2. Correlations observed in the ROESY NMR spectrum, and a series of higher resolution SELROESY spectra, of **1** verified that the diene portion of the DMAdda^5^ residue was *trans*-substituted and had not been epimerized to a cisoid analogue [34]. In particular, H-4 (5.55 ppm) showed ROESY and SELROESY correlations to DMAdda’s NH (8.16 ppm), H-2 (3.11 ppm), 6-Me (1.69 ppm) and 2-Me (1.05 ppm) resonances, while H-7 (5.45 ppm) showed strong correlations to H-5 (6.25 ppm), H-9 (3.60 ppm), H-10_a/b_ (2.59/2.82 ppm), 6-Me (1.69 ppm) and 8-Me (1.02 ppm). ROESY and SELROESY data also showed the preferred solution conformation in the vicinity of the d-Glu^6^, Mdha^7^, d-Ala^1^, Leu^2^ and d-Masp^3^ residues of **1** to be similar to that reported by Trogen et al. [35] for **9**. For example, the d-Glu^6^-NH signal (8.33 ppm) of **1** exhibited a strong ROESY correlation to the DMAdda^5^ H-2 (3.11 ppm) signal and lower intensity correlations to the d-Glu^6^ H-2 (4.10 ppm) and d-Glu^6^ H-3_a_ and H-3_b_ (1.91 and 2.12 ppm) signals, while the Leu^2^-NH (8.30 ppm) exhibited strong to moderate ROESY correlations to d-Masp^3^-NH (7.69 ppm), d-Ala^1^ H-2 (4.57 ppm), Leu^2^ H-2 (4.28 ppm), Leu^2^ H-3b (2.04 ppm) and Leu^2^ H-4 (1.78 ppm).

Other than for the ^1^H and ^13^C NMR signals arising from the Har residue of **2**, there was a close correspondence between the ^1^H and ^13^C assignments of **2** with those established for **1** (Table 2). The H-2 signal of Har (4.35 ppm) exhibited a COSY correlation to the pair of non-equivalent H-3 protons of the Har residue at 1.52 and 2.02 ppm, respectively, while longer range correlations observed in DIPSI2 and in higher resolution 1D-SELTOCSYspectra performed with mixing times of 80 and 160 ms identified the resonances attributable to the H-3_a/b_ (1.32 and 1.37 ppm) and H-4 (1.54 ppm) methylene protons and the H-5 methine proton (3.13 ppm) of the Har residue of **2**. The ^1^H and ^13^C shifts of **2** were correlated in an HSQC spectrum, and in the case of the Har C-4 signal at 29.38 ppm, a higher resolution SHSQC spectrum differentiated it from the d-Glu C-3 signal at 29.42 ppm.

ROESY correlations analogous to those observed for **1** were also observed for **2**, indicating that, notwithstanding the presence of a Har residue in **2** compared to an Arg residue in **1**, the preferred solution confirmation of the MC ring system of **2** was similar to that of **1** and comparable to those previously reported [35] for **9** and **12**. This conclusion is also consistent with the finding that the ^1^H and ^13^C shifts of the amino acid residues present in **1** and **2**, other than parts of their DMAdda^5^ and Har^4^ units, were very similar to those previously reported for MC-LR (**9**) [3]. NMR supporting data can be accessed in the SI file (Figs. S9–S31).

### 3.2 Adda-ELISA and PP2A inhibition assay

**Fig. 4:**
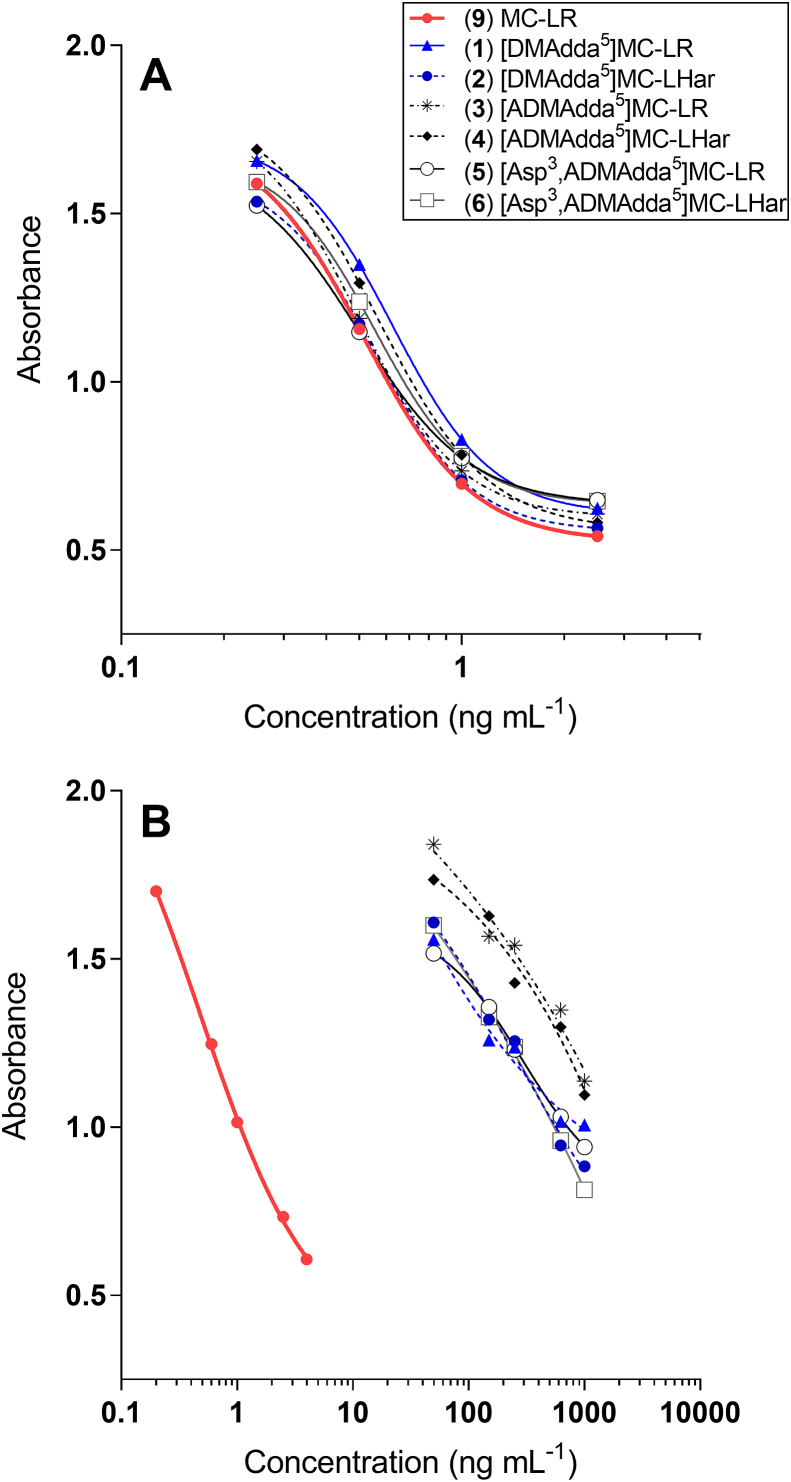
PP2A inhibition (A) and Adda-ELISA (B) from analysis of MC-LR (**9**), [DMAdda^5^]MCs (**1** and **2**) and [ADMAdda]MCs (**3**–**6**). Curves are from fitting the data to a 4-parameter logistic model.

The Adda-ELISA did not react to the purified [ADMAdda^5^]MCs (**1** and **2**) or [DMAdda^5^]MCs (**3**–**6**) at concentrations of 1 or 10 ng mL^−1^. Therefore, higher concentrations were tested (50–1000 ng mL^−1^), with IC_50_ values determined to be > 200 ng mL^−1^ as compared 0.49 ng mL^−1^ for MC-LR (**9**) (Table S3; Fig. 4 and S32), giving cross-reactivities of under 0.25% relative to MC-LR. This is unsurprising given the assay design concept, as the Adda-ELISA was developed to recognize the unmodified Adda epitope [19]. Although some reports suggest that the Adda-ELISA responds to MC congeners containing modified Adda moieties [8], this work demonstrates that the cross-reactivity is very low. Low cross-reactivity with [ADMAdda^5^]MCs was also reported with anti-MC-LR polyclonal antibodies [11], indicating the need for alternative approaches to MC ELISA antibody development if MC congeners containing modified Adda moieties are to be quantified by immunoassay methods.

The PP2A inhibition assay indicated that all of the ADMAdda- and DMAdda-containing MCs isolated in this work (**1**–**6)** had similar inhibitory potencies (IC_50_ 0.37–0.52 ng mL^−1^) to MC-LR (**9**) (0.42 ng mL^−1^) (Fig. 4, Table S4). Other work has also indicated the toxic potential of MCs containing ADMAdda/DMAdda to be similar to those of Adda-containing MCs. For instance, [ADMAdda^5^]MCs isolated from a *Planktothrix* sp., tentatively identified as [d-Asp^3^,ADMAdda^5^]MC-HtyR and [d-Asp^3^,ADMAdda^5^]MC-LR, exhibited similar PP2A inhibition to that of MC-LR [11]. Purified MCs extracted from *Nostoc* sp. strain 152, including the [ADMAdda^5^]MCs purified in this work (**3**–**6**), retained their hepatotoxicity when administered intraperitoneally (i.p.) to mice [5]. The LD_50_ (i.p.; mice) was found to be similar to that of MC-LR (**9**) for [ADMAdda^5^]MC-LR (**3**) and [ADMAdda^5^]MC-LHar (**4**), at 60 μg kg^−1^ body weight (bw), with [d-Asp^3^,ADMAdda^5^]MC-LR (**5**) being slightly less toxic (LD_50_ of 160 μg kg^−1^ bw) [5]. The LD_50_ (i.p., mice) for [DMAdda^5^]MC-LR (**1**) was also determined to be 97 μg kg^−1^ bw, only slightly less toxic than for MC-LR (**9**) [36]. The toxicity of [DMAdda^5^]MC-LHar (**2**) has not been previously determined, but PP2A inhibition data reported here suggests a similar toxic potential to that of MC-LR.

### 3.3 Oxidative cleavage experiments

**Fig. 5:**
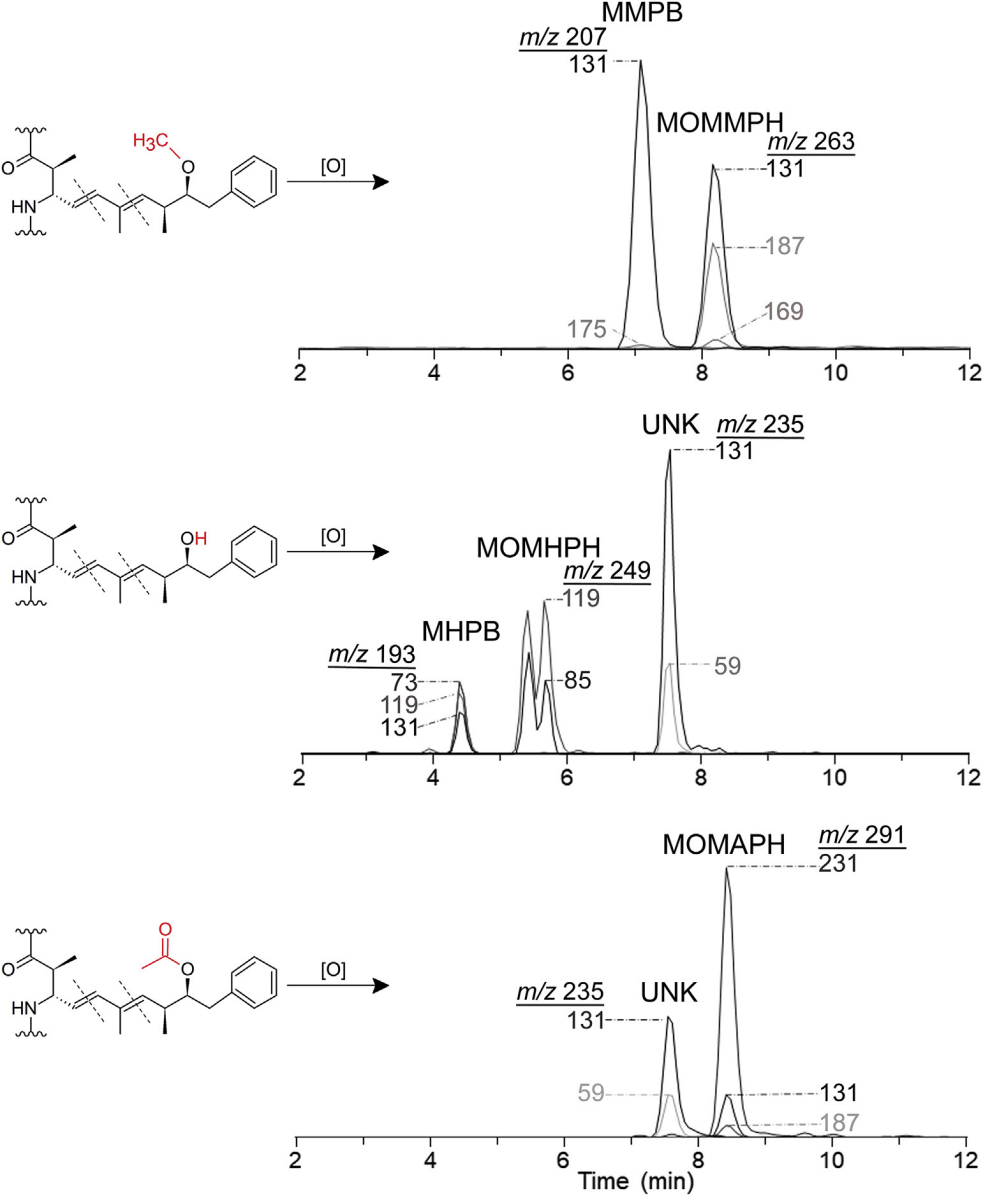
Triple quadrupole LC-MS/MS (negative ionization) chromatograms of targeted oxidation products formed from: MC-LR (**9**; top); [DMAdda^5^]MC-LR (**1**; middle), and; [ADMAdda^5^]MC-LR (**3**, bottom). Includes observed product ions, many of which were shared between structures, such as *m*/*z* 131. The unknown oxidation product (UNK) observed from both DMAdda and ADMAdda (at 7.55 min with *m*/*z* 235) was not unique and therefore excluded from routine analyses.

The MCs purified in this work were used to develop an oxidative cleavage procedure to test for total MCs and NODs based on the MMPB approach for Adda-containing MCs [24,28]. Oxidative cleavage of [ADMAdda^5^]MC-LR (**3**) and [DMAdda^5^]MC-LR (**1**) (Fig. 1) produced compounds (Fig. 2) analogous to MMPB (from MC-LR (**9**)) that were initially observed in total ion LC–MS spectra (*m*/*z* 180–300) (Fig. S33). Oxidation of **9** produced prominent peaks with *m*/*z* 207 (MMPB) and 263 (MOMMPH). Peaks from oxidized [DMAdda^5^]MC-LR were observed with *m*/*z* 193 (MHPB), 235, and 249 (MOMHPH). Finally, oxidation products from [ADMAdda^5^]MC-LR resulted in LC–MS peaks at *m*/*z* 235, 291 (MOMAPH) and 231. LC–MS/MS experiments were used to establish tentative structural identities and to verify that oxidation products were conserved across congeners containing the same type of Adda variant. Similar to the chromatographic behavior of intact MCs (elution of DMAdda-, followed by Adda-, and finally ADMAdda-containing congeners), the oxidized products followed the same order of retention, with the smaller molecules (MHPB, MMPB, MAPB) eluting approximately 1 min prior to their larger counterparts (MOMHPH, MOMMPH, MOMAPH) (Fig. 5 and S34).

During the oxidative cleavage of the ADMAdda in **3**, the expected smaller compound MAPB, formed through cleavage of the 6,7-ene, was initially targeted. However, although a peak with a pseudomolecular ion corresponding to MAPB (*m*/*z* 235 in negative mode) was detected after oxidation, a peak with the same retention time and product ion spectrum was also observed after oxidation of [DMAdda^5^]MC-LR (**1**) (Fig. 5 and S35). Oxidation of the Adda-containing MC-LR (**9**) did not result in the formation of this compound, so *m*/*z* 235 was therefore considered to be unique to ADMAdda and DMAdda, but not Adda. Since the *m/z* 235 peak was not unique to ADMAdda, further investigations using MAPB as a diagnostic compound were abandoned. Rather, the compound formed through cleavage of 4,5-ene, and exhibiting [M−H]^−^ at *m*/*z* 291, was assessed as a unique conserved product from oxidative cleavage of ADMAdda-containing MCs.

**Fig. 6:**
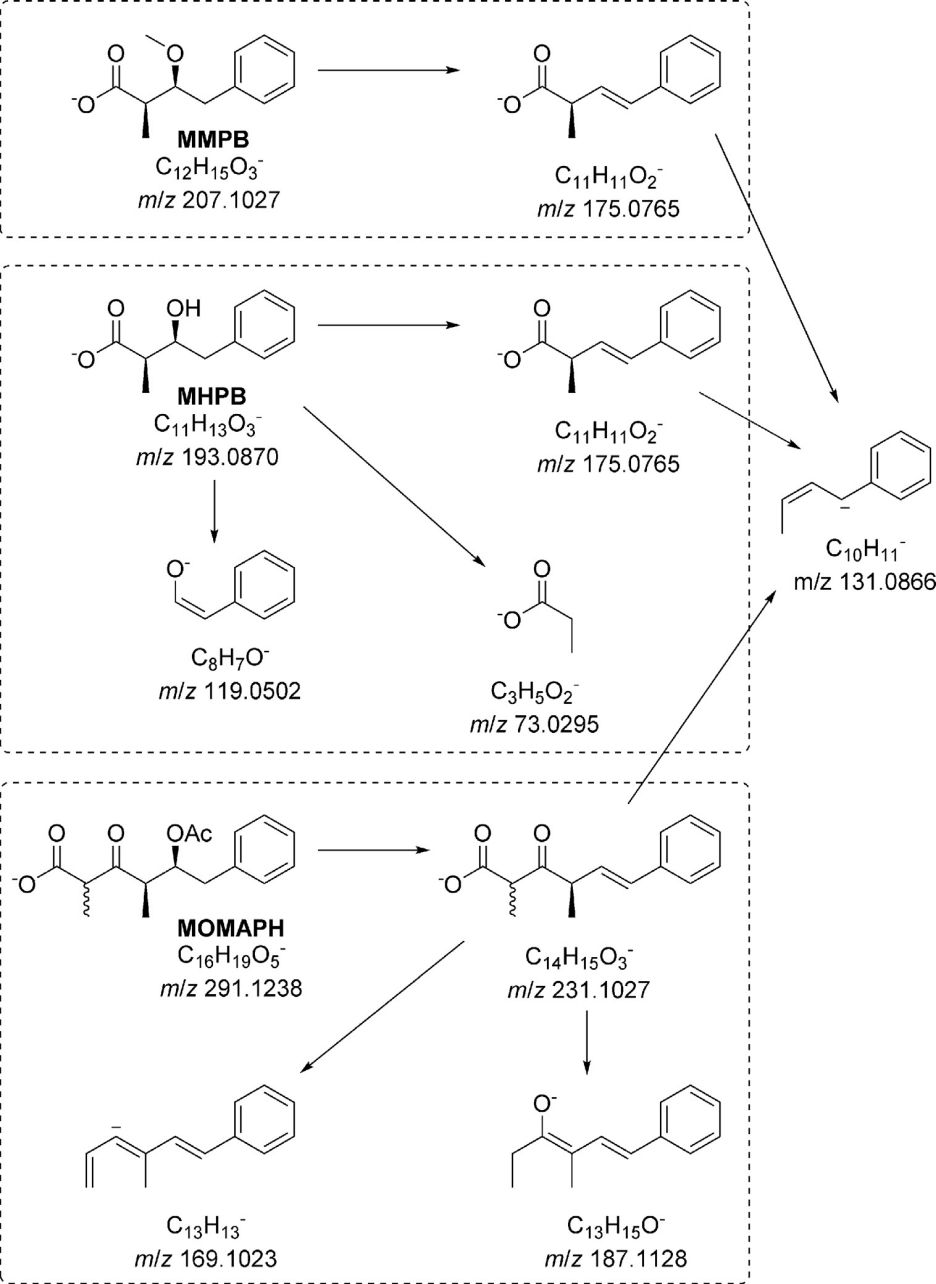
Proposed fragmentation of MMPB, MHPB and MOMAPH in negative ionization mode. Note the production of a common product ion at *m*/*z* 131.1, which can be seen in the LC–HRMS/MS spectra of MOMAPH in Fig. 7 and in the LC–MS/MS chromatograms in Fig. 5.

**Fig. 7:**
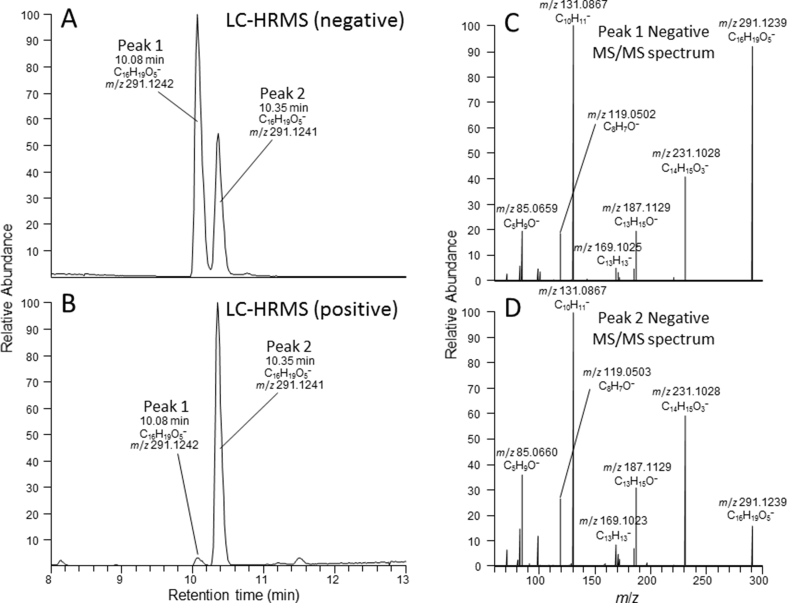
LC–HRMS chromatograms (A, negative mode, extracted *m*/*z* 290–292; B, positive mode, extracted *m*/*z* 290–312) of the isolated compound (MOMAPH) formed through the oxidative cleavage of ADMAdda. Panels C and D show the negative mode product-ion spectra of peaks 1 and 2, respectively. The elemental compositions shown on the product-ion spectra were all within 0–1.2 ppm, and were the only viable compositions within 5 ppm, of the measured *m*/*z* values.

The target used in analysis of oxidized ADMAdda, isolated and characterized in this work, was MOMAPH (possibly together with its corresponding enol), which was confirmed using both low- and high-resolution mass spectrometry. LC-MS/MS analyses in negative ionization of the peak with *m*/*z* 291 (MOMAPH) showed a facile neutral loss of acetic acid (60 Da) to give a product ion at *m*/*z* 231. Low resolution MS/MS spectra of MOMAPH showed the most intense product ion at *m*/*z* 231, with a weaker product ion at *m*/*z* 131 (Figs. S36 and S37). MS/MS fragmentation of the product ion at *m*/*z* 231 ([M−H–CH_3_CO_2_H]^−^) gave MS^3^ product ions at *m*/*z* 131 (100%), 187 (26%) and 169 (3%) (Fig. S38). Positive ionization MS/MS spectra of [M+H]^+^ of MOMAPH at *m*/*z* 293 resulted in data-rich spectra (Figs. S39 and S40), which were analyzed together with the negative ionization MS/MS spectra (Fig. 6) in the structure elucidation. Purification of the oxidized product (Fig. S41) followed by LC–HRMS/MS analysis showed it to be composed of two isomeric forms, with *m*/*z* 291.1242 in negative and *m*/*z* 293.1380 in positive ionization modes, although the later-eluting isomer formed a prominent ammonium adduct ion in positive ionization mode (Fig. 7). LC–HRMS was consistent with a neutral elemental composition of C_16_H_20_O_5_ for both peaks, and LC–HRMS/MS data (Fig. 7) was also consistent with the proposed product ion identities (Fig. 6).

**Fig. 8:**
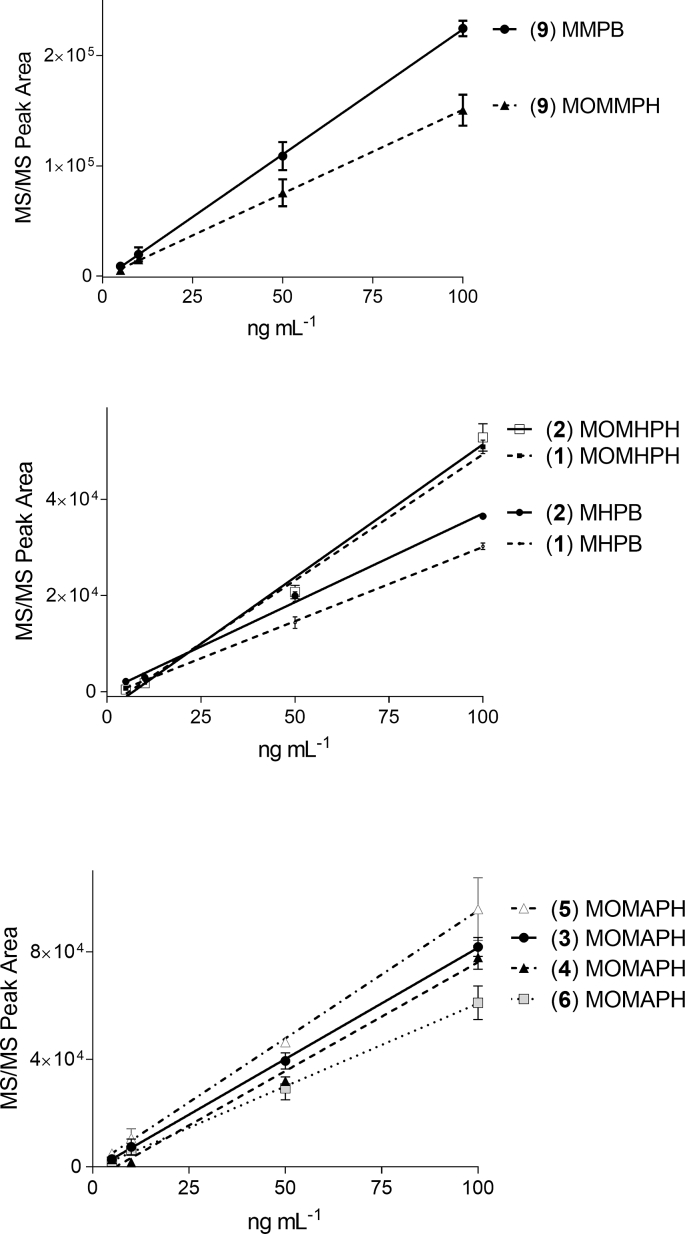
Triple quadrupole LC–MS/MS peak areas for unique oxidative cleavage products versus the concentration of the intact MC that was oxidized to produce them, for: top, MC-LR (**9**); middle, [DMAdda^5^]MCs (**1** and **2**), and; bottom, [ADMAdda^5^]MCs (**3**–**6**). Each curve is plotted with the mean (n = 2) and error bars (standard deviation).

[ADMAdda^5^]MCs purified in this work (**3**–**6**) were used to prepare 5-point standard curves ranging from 1 to 100 ng mL^−1^ and oxidized, and the resultant oxidation product, MOMAPH, was analyzed via LC-MS/MS (Fig. 8). The standards produced similar response curves exhibiting linear coefficients of determination (R^2^) ≥ 0.992. The differences observed in MOMAPH formation may be attributed to variability introduced during quantification of the original standard (via HPLC–UV relative to MC-LR), variability in oxidation efficiency specific to analyte chemistry, or the competition of oxidant with the two closely located alkenes. Differences in molecular weight of the intact congeners varied less than 3% and could not have significantly contributed to observed differences in MOMAPH production.

LC–MS/MS experiments conducted on oxidatively cleaved [DMAdda^5^]MCs confirmed the formation of MHPB (*m*/*z* 193; negative ionization) via cleavage of the 6,7-ene (Fig. 2). Product-ion spectra from the linear ion trap and triple-quadrupole MS were similar, with product ions at *m*/*z* 73 (C_3_H_5_O_2_^−^), 119 (C_8_H_7_O^−^) and 131 (C_10_H_11_^−^) observed at varying relative intensities (Figs. S42 and S43). Oxidation products formed through cleavage of 4,5-ene of the DMAdda moiety were observed as two chromatographic peaks eluting approximately 1 min after MHPB (Fig. 5). These two products may represent MOMHPH and its corresponding enol, or an additional isomer produced via keto–enol tautomerism. Both isomers shared common product ions at *m*/*z* 119 (C_8_H_7_O^−^) and 85 (C_5_H_9_O^−^) (Fig. S44). The peak areas of both isomers were combined (integration of both peaks) for the time-course assessment and calibration curves, but ultimately, MHPB was used for analysis of field samples. Standard curves (Fig. 8) derived from the oxidative cleavage of both [DMAdda^5^]MCs (**1** and **2**) show that MHPB formed less efficiently from [DMAdda^5^]MC-LR (**1**) than from [DMAdda^5^]MC-LHar (**2**). In contrast, the opposite situation was observed for the formation of MOMHPH and MHPB from **1** and **2**, indicating that differences in compound chemistry affect the cleavage reaction even with two very closely related alkenes.

The LC–MS/MS analyses of oxidized MC-LR (**1**) confirmed the presence of MMPB, with a prominent product ion at *m*/*z* 131 (C_10_H_11_^−^) from both ion trap and triple-quadrupole MS systems (Figs. S45 and S46). The triple-quadrupole MS also gave a low-intensity product ion at *m*/*z* 175 (C_11_H_11_O_2_^−^) (Fig. 5). The oxidation product of unmodified Adda, through cleavage of the 4,5-ene, was also observed with [M−H]^−^ at *m*/*z* 263. The compound likely represents MOMMPH and its enol tautomer and/or related isomers. Product ions observed (Fig. S47) included *m*/*z* 231 (C_14_H_15_O_3_^−^), 187 (C_13_H_15_O^−^), 169 (C_13_H_13_^−^), and 131 (C_10_H_11_^−^), further supporting its structural identity. The formation of additional oxidation products from Adda could explain the lower reported recoveries of MMPB post-oxidation and -extraction in other studies [37]. Although MOMMPH might provide an alternative for the determination of total Adda MCs, the use of MMPB for quantitative analysis of total Adda-containing MCs has been well established [28]. Because it is also unknown whether MOMMPH exists in nature or could be produced by oxidation of other endogenous compounds, MMPB was used for routine sample analysis. However, monitoring of MOMMPH is warranted to determine its applicability as a quantitative metric for total Adda-containing MCs in future work.

Oxidation products were not assessed for stability during long-term storage, but were stable in water or 5% methanol during short term storage (≤30 d; −20 °C). The mechanism(s) driving the cleavage of the 4,5-ene vs. the 6,7-ene were not explored in this work. Rather, conserved products were chosen and applied to the analysis of field collections. However, since the two olefinic sites of oxidative cleavage are in close proximity (Fig. 2), it is possible that the oxidation conditions used in this study played a role in the relative oxidation product concentrations (Fig. 8). The KMnO_4_ treatment of microcystins at neutral pH results in oxidation of both the 4,5-ene and 6,7-enes of the Adda to give α-hydroxyketones, which are further oxidized to produce cleavage products (e.g. carboxylic acid) [38]. Olefins oxidized with Lemieux reagent, similar to the oxidant used in this study, showed that KMnO_4_ first converted olefins to hydroxyketones, which were rapidly cleaved by NaIO_4_, and products further oxidized by KMnO_4_ [39]. The use of NaIO_4_ is thought to allow the reaction to proceed with high specificity and at a faster rate than when using KMnO_4_ alone [39]. While reaction conditions were not modified in this work to assess their effect on product formation, several parameters could easily be adjusted (e.g. pH, temperature) to affect reaction rates and yields.

#### 3.3.1 Time course

**Fig. 9:**
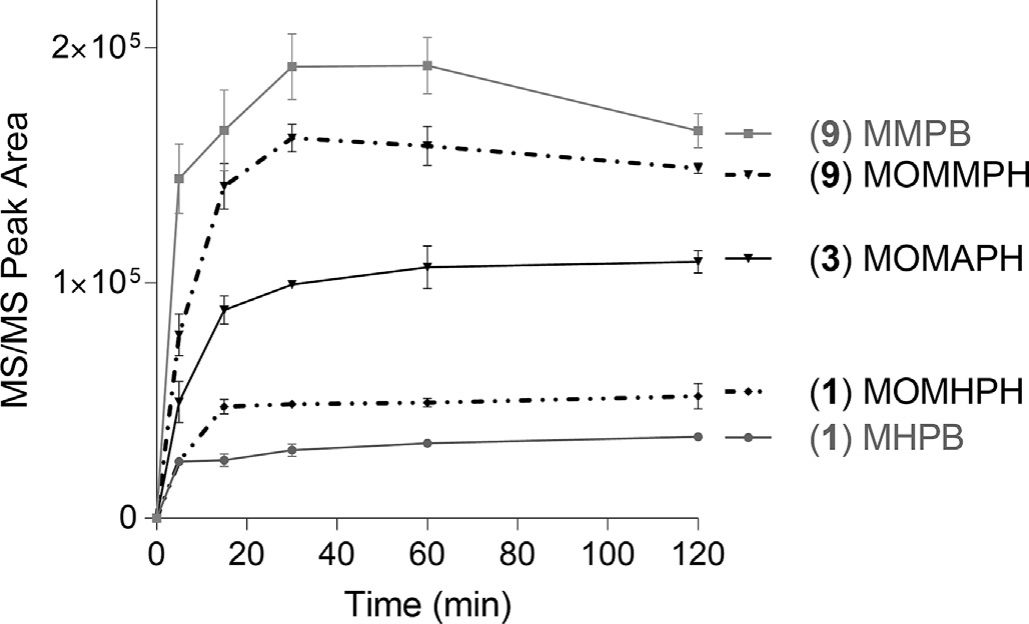
Time course for production of the targeted oxidative cleavage products (peak areas by Triple quadrupole LC–MS/MS), plotted as means (n = 3) with error bars showing the standard deviation, from: MC-LR (**9**) (MMPB, MOMMPH); [ADMAdda^5^]MC-LR (**3**) (MOMAPH), and; [DMAdda^5^]MC-LR (**1**) (MHPB, MOMHPH).

Oxidative cleavage products reached ≥90% of their maximum concentrations within 30 min (Fig. 9). The 6,7-ene cleavage products (MHPB, MMPB) formed faster (75% in 5 min) than their counterparts from cleavage of the 4,5-ene (46–48% in 5 min). However, losses of MMPB were observed between 60 and 120 min, in accord with other studies that report degradation of MMPB over time [26]. Based on these observations, oxidation reaction times were limited to 60 min.

### 3.4 Sample analyses

The *Nostoc* sp. strain 152 culture and three lyophilized grab samples collected from the ‘West Coast’, ‘Midwest’ and ‘East Coast’ of the USA were extracted and analyzed by four different techniques (Table 3). A targeted LC-MS/MS method (21 MCs and NOD-R) was compared to the Adda-ELISA, PP2A inhibition assay, and the new oxidative cleavage procedure. The main congener from the west coast *Dolichospermum*-dominated bloom was MC-LR (**9**), making up 91% of the targeted MC detections by LC–MS/MS. The Adda-ELISA, PP2A inhibition, and oxidative cleavage analyses indicated approximately 20% more MCs to be present than those detected by LC–MS/MS. The desmethylated Adda variant [DMAdda^5^]MC-LR (**1**) was confirmed, accounting for 1.3% of the total targeted MCs and 1.4% relative to MC-LR. Oxidative cleavage with analysis for MHPB indicated a similar amount of DMAdda (1.8%). The sum of MCs was 1425 μg g^−1^ by oxidative cleavage, indicating that >80% of identified MCs were accounted for by LC–MS/MS. This was supported by both the PP2A inhibition assay (1400 μg g^−1^ MC-LR equivalents) and Adda-ELISA (1500 μg g^−1^ Adda-containing MCs).

**Table 3:** Concentrations of MCs (μg g^−1^ dry weight) in crude extracts of bloom material and *Nostoc* sp. 152 culture using 4 techniques: targeted LC-MS/MS analysis, Adda-ELISA, PP2A inhibition assay, and total MCs/NODs by oxidative cleavage. Standard deviations for multiple extractions (when conducted) and the lowest achieved method detection limits (MDLs) are also shown. NOD-R and [d-Asp^3^]MC-RR (**21**) were the only targeted analytes not detected.

MDL (μg g^−1^)	ID	Metric	West Coast (*Dolichospermum*)	Midwest (*Microcystis*)	East Coast (*Microcystis*)	*Nostoc* sp. strain 152
1.0	**1**	[DMAdda^5^]MC-LR	14	2.0		7.3
0.5	**2**	[DMAdda^5^]MC-LHar				5.9
0.5	**12**	MC-RR	1.5	35		
1.0	**13**	MC-YR		31		
1.0	**14**	MC-HtyR	4.3			
0.5	**9**	MC-LR	1050	70	30	
1.0	**8**	[Dha^7^]MC-LR	50	2.7		
0.5	**3**	[ADMAdda^5^]MC-LR				317
0.5	**7**	[d-Asp^3^]MC-LR	13	1.5		
2.0	**5**	[d-Asp^3^,ADMAdda^5^]MC-LR				76
0.5	**4**	[ADMAdda^5^]MC-LHar				236
1.0	**10**	MC-HilR	7.4	5.3		
1.0	**6**	[d-Asp^3^,ADMAdda^5^]MC-LHar				50
1.0	**15**	MC-WR		6.5		
1.0	**11**	[d-Leu^1^]MC-LR			356	
0.5	**16**	MC-RY		1.5		
0.5	**17**	MC-LA	6.7	1420		
0.5	**18**	MC-LY		8.7		
0.5	**19**	MC-LF		1.9		
0.5	**20**	MC-LW		1.9		
		**SUM targeted MCs**	**1148**	**1588**	**386**	**692**
		*% Adda*	*98.7%*	*99.9%*	*100%*	*0.0%*
0.2		**Adda-ELISA**	**1500** ± 50	**1700** ± 370	**350** ± 50	**2.2** ± 0.9
0.3		**PP2A inhibition**	**1400** ± 8	**1500** ± 0	**400** ± 9	**1000** ± 22
10		**Total [DMAdda]**	25 ± 9	140 ± 48	10 ± 1	21 ± 3
5		**Total [Adda]**	1400 ± 110	1700 ± 270	610 ± 50	<5
10		**Total [ADMAdda]**	<10	<10	<10	588 ± 80
		**SUM Oxidized**	**1425**	**1840**	**620**	**609**
		*% Adda*	*98%*	*92%*	*98%*	*0%*

**Fig. 10:**
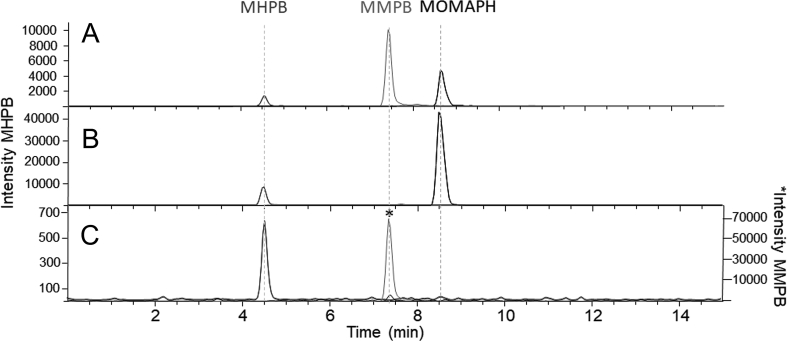
Triple quadrupole LC-MS/MS chromatograms showing the method for analysis of total MCs and NODs through oxidative cleavage followed by analysis for MHPB, MMPB and MOMAPH. A, an oxidized mixed standard of MC-LR (**9**), [ADMAdda^5^]MC-LR (**3**), and [DMAdda^5^]MC-LR (**1**), each at 10 ng mL^−1^; B, *Nosto*c sp. strain 152 culture containing [ADMAdda^5^]MCs and [DMAdda^5^]MCs, and; C, a field sample collected from a Midwest *Microcystis* bloom containing predominantly [Adda^5^]MCs. Note the secondary scale (denoted by an asterisk) for MMPB response, which is 2-orders of magnitude higher than for MHPB in panel C.

The targeted LC–MS/MS analysis of the Midwest *Microcystis* bloom showed it was dominated by MC-LA (**17**) (1420 μg g^−1^), making up 89% of the total targeted MCs. Only a small amount of [DMAdda^5^]MC-LR (**1**) was detected (2 μg g^−1^), which was 2.8% that of its methylated counterpart, MC-LR (**9**). However, oxidative cleavage indicated 140 μg g^−1^ DMAdda (7.6% of total oxidized MCs) to be present (Table 3, Fig. 10). LC–HRMS/MS analysis indicated that [DMAdda^5^]MC-LA was present (Fig. S48), but this was not targeted for quantification in the LC–MS/MS analysis due to a lack of a suitable reference material. The sum of MCs by oxidative cleavage was 1840 μg g^−1^, with 86% of these MCs accounted for in the targeted LC–MS/MS. Total MC/NOD concentrations by oxidative cleavage were similar to those obtained by PP2A inhibition (1500 μg g^−1^ MC-LR equivalents) and Adda-ELISA (1700 μg g^−1^ Adda-containing MCs).

The targeted LC–MS/MS analysis of MCs in the bloom collected from the Poplar reservoir in the Chesapeake Bay revealed the predominance of [d-Leu^1^]MC-LR together with lower levels of MC-LR, as reported previously [27]. Only two variants were present above method detection limits, but previous work showed contributions of at least 25 additional minor variants of MC that were not targeted in this work due to a lack of available calibration standards. Oxidative cleavage of the bloom material revealed the presence of DMAdda (10 μg g^−1^), which was likely primarily from contributions of [d-Leu^1^,DMAdda^5^]MC-LR previously reported in the sample [27] but not targeted in this study. If the 3% contribution of DMAdda relative to Adda followed the same pattern, the level of [DMAdda^5^]MC-LR would have been 0.9 μg g^−1^, which is below the method detection limit (1 μg g^−1^). The Adda-ELISA (350 μg g^−1^) and PP2A inhibition (400 μg g^−1^) analyses indicated that most of the MCs were accounted for in the targeted LC–MS/MS analysis (sum 386 μg g^−1^), with the total by oxidative cleavage (620 μg g^−1^) possibly representing some decomposed MCs (unrecognized by the ELISA) (stored 7 y).

MOMAPH (from oxidation of ADMAdda) was not detected in any of the three oxidized planktonic bloom extracts, but was quantitatively measured in the *Nostoc* sp. strain 152 culture using the oxidative cleavage procedure (Table 3, Fig. 10). The sum of [ADMAdda^5^]MCs by LC–MS/MS was 679 μg g^−1^, 15% higher than the estimate using the oxidative cleavage method (588 μg g^−1^ total ADMAdda-MCs). The concentration of DMAdda by oxidative cleavage was 21 μg g^−1^, almost double the total [DMAdda^5^]MCs targeted by LC–MS/MS (13 μg g^−1^), although the basic conditions of the oxidation procedure (pH > 10) may have caused partial hydrolysis of the acetate group, which might account for this. Kinetic data from Ballot et al. (2014) [40] indicate that in 1 h at 30 °C in pH 9.7 carbonate buffer, ca 2.3% of the ADMAdda-acetate groups would be hydrolysed, which would have generated an additional 13 μg g⁻^1^ of DMAdda-equivalents. The [DMAdda^5^]MC-LR (**1**) and [DMAdda^5^]MC-LHar (**2**) levels represented 2.3% and 2.5% of their ADMAdda-counterparts, respectively. Total DMAdda was slightly higher at 3.4% of total ADMAdda as determined by oxidative cleavage, but can be accounted for by hydrolytic cleavage of the acetate groups of the [ADMAdda^5^]MCs and their oxidative cleavage products.

As observed with the purified standards of [DMAdda^5^]MCs and [ADMAdda^5^]MCs, the Adda-ELISA showed very low cross-reactivity with crude extracts *Nostoc* sp. strain 152. The MCs measured with the other three methods were >250 times the level measured by the Adda-ELISA. While the Adda-ELISA was not representative of the toxic MC content, the PP2A inhibition assay detected 1000 μg g^−1^ MC-LR equivalents, which was higher than the sum of MCs by oxidative cleavage (609 μg g^−1^) and the sum of MCs targeted by LC–MS/MS (692 μg g^−1^).

The results of samples analyzed in this work and others [16,40,41] support that low levels (≤10%) of [DMAdda^5^]MCs are frequently present when the profile is dominated by Adda-containing MCs. This suggests that DMAdda may be present due to incomplete *O*-methylation during MC biosynthesis [40,41], or perhaps due to demethylation during degradation of [Adda^5^]MCs. Due to a lack of commercially available standards and their early elution, the presence of DMAdda-containing MCs is likely underreported. Similarly, [ADMAdda^5^]MCs in samples may also be underreported due to the apparent absence or low contribution of [Adda^5^]MCs by cyanobacteria that produce [ADMAdda^5^]MCs. While commonly employed analysis techniques (e.g. ELISA, targeted LC-MS/MS) might account for the Adda contribution, any [ADMAdda]MCs or [DMAdda]MCs could easily be overlooked.

## 4 Conclusion

The comprehensive analysis of microcystins (MCs) and nodularins (NODs) is challenging due to the numerous structural variations that may be present in a given sample. Although broad-specificity analytical techniques are able to account for some modifications, methods targeting the Adda moiety failed to detect ADMAdda^5^-and DMAdda^5^-containing MCs. Furthermore, the lack of commercially available standards hampers targeted analysis approaches. This is problematic, as protein phosphatase inhibition assays (this work and [11]) and mouse bioassays [5,36] indicate the toxic potential of ADMAdda^5^-and DMAdda^5^-containing MCs to be similar to that of MC-LR (**9**). Therefore, the existing oxidative cleavage and analysis for Adda-containing MCs and NODs (*i.e.* the MMPB method) was augmented to include ADMAdda and DMAdda variants to achieve a comprehensive analysis of total MCs and NODs.

During the investigation of the chemical oxidation of MCs, it was determined there were two sites for the oxidative cleavage of Adda, ADMAdda, and DMAdda, leading to multiple oxidation products that could be targeted for analysis. This observation is important, as the competitive oxidation of the two olefinic sites could impact quantitation if not carefully calibrated using pre-oxidation standard addition with representative congeners (e.g. [DMAdda^5^]MC-LR, [ADMAdda^5^]MC-LR). Utilizing standard addition, the oxidative cleavage and analysis procedure applied to field samples was not only helpful in qualifying results, but was also successful in estimating the total Adda-, DMAdda- and ADMAdda-containing MCs in the samples. Data and interpretations were confirmed using LC-MS/MS, PP2A inhibition assay, and the Adda-ELISA.

As described in this work, the oxidation and analysis of total MCs/NODs can be implemented in any laboratory with LC-MS/MS capability and does not require more than a single representative standard for each form for calibration. Future work employing this technique may assist in the determination of the rarely reported ADMAdda- and DMAdda-containing MCs and NODs. This is especially important for testing bloom forming species that possess the ability to nearly exclusively produce [ADMAdda^5^]MCs, such as *Planktothrix agardhii* strain PH-123 [11]. The method may also be useful for total MC/NOD analysis after cyanobacterial exposure events to account for free, protein-bound, and conjugated fractions in more complicated matrices (e.g. tissues).

## Funding

This work was partly supported by the 10.13039/501100005416Research Council of Norway through the Norwegian NMR Platform, NNP (226244/F50).

## CRediT authorship contribution statement

**Amanda J. Foss:** Conceptualization, Methodology, Investigation, Writing - original draft, Writing - review & editing, (PP2A, ELISA, LC-MS/MS), Writing. **Christopher O. Miles:** Investigation, (LC–HRMS/MS), Writing - original draft, Writing - review & editing. **Alistair L. Wilkins:** Investigation, (NMR), Writing - original draft, Writing - review & editing. **Frode Rise:** Investigation, (NMR), Resources. **Kristian W. Trovik:** Investigation, (NMR). **Kamil Cieslik:** Investigation, (ELISA). **Mark T. Aubel:** Resources, Supervision.

## Declaration of competing interest

The authors declare that they have no known competing financial interests or personal relationships that could have appeared to influence the work reported in this paper.
