# Supplementary material for: Analysis of total microcystins and nodularins by oxidative cleavage of their ADMAdda, DMAdda, and Adda moieties
Source: Anal Chim Acta X. 2020 Sep 2;6:100060. doi: 10.1016/j.acax.2020.100060 (PMC7772689; doi:10.1016/j.acax.2020.100060)
Supplement: Multimedia component 1 [file mmc1.pdf]

Supplementary Information for: Analysis of total microcystins and nodularins by oxidative cleavage of the ADMAdda, DMAdda, and Adda moieties

Amanda J. Foss[1*], Christopher O. Miles[2], Alistair L. Wilkins[3,4,5], Frode Rise[5], Kristian W. Trovik[5], Mark T. Aubel[1], Kamil Cieslik[1]

[1]GreenWater Laboratories/CyanoLab, 205 Zeagler Drive, Palatka, Florida 32177, USA
[2]Measurement Science and Standards, National Research Council, 1411 Oxford Street, Halifax, NS, B3H 3Z1, Canada
[3]Norwegian Veterinary Institute, P. O. Box 750 Sentrum, N-0106 Oslo, Norway
[4]Chemistry Department, University of Waikato, Private Bag 3105, 3240 Hamilton, New Zealand
[5]Department of Chemistry, University of Oslo, P.O. Box 1033, N-0315 Oslo, Norway

Tables:

Table S1: Gradient conditions employed for semi-preparative HPLC S3
Table S2: MRM transitions monitored for intact congeners of MC and NOD-R S4
Table S3: Adda-ELISA data for MC-LR (**9**), [DMAdda[5]]MCs (**1**, **2**) and [ADMAdda[5]]MCs (**3**–**6**) S5
Table S4: PP2A Inhibition Assay data for MC-LR (**9**), [DMAdda[5]]MCs (**1**, **2**) and [ADMAdda[5]]MCs S6

Figures:

Figure S1: Method 1; Semi-preparative HPLC–UV chromatogram S7
Figure S2: Semi-preparative HPLC–UV chromatogram S8
Figure S3: Semi-preparative HPLC–UV chromatograms S9
Figure S4: Semi-preparative HPLC–UV chromatogram S10
Figure S5: Manufacturer Instructions for PP2A Inhibition AssaY. S11
Figure S6: Ion trap LC–MS/MS chromatograms and MS/MS spectra OF 1, 3, 5. .... S12
Figure S7: Ion trap LC–MS/MS chromatograms and MS/MS spectra OF 2, 4, 6 .... S13
Figure S8: Ion Trap LC–MS/MS calibration curves for intact MCs **1**–**6** S14
Figure S9: ^{1}H NMR spectrum of [DMAdda[5]]MC-LR (**1**) S15
Figure S10: Expansion (0.6–4.6 ppm) of the ^{1}H NMR spectrum of [DMAdda[5]]MC-LR (**1**) S16
Figure S11: Expansion (4.7–8.7 ppm) of the ^{1}H NMR spectrum of [DMAdda[5]]MC-LR (**1**) S17
Figure S12: DEPT135 NMR spectrum of [DMAdda[5]]MC-LR (**1**) S18
Figure S13: Expansion (10–60 ppm) of the DEPT135 NMR spectrum of [DMAdda[5]]MC-LR (**1**) S19
Figure S14: Expansion (60–160 ppm) of the DEPT135 NMR spectrum of [DMAdda[5]]MC-LR (**1**) S20
Figure S15: DEPTQ NMR spectrum of [DMAdda[5]]MC-LR (**1**) S21
Figure S16: Expansion (167–185 ppm) of the DEPTQ NMR spectrum of [DMAdda[5]]MC-LR (**1**) S22
Figure S17: COSY NMR spectrum of [DMAdda[5]]MC-LR (**1**) S23
Figure S18: DIPSI2 (80 ms) NMR spectrum of [DMAdda[5]]MC-LR (**1**) S24
Figure S19: Edited HSQC135 NMR spectrum of [DMAdda[5]]MC-LR (**1**) S25
Figure S20: HMBC NMR spectrum of [DMAdda[5]]MC-LR (**1**) S26
Figure S21: ^{1}H NMR spectrum of [DMAdda[5]]MC-LHar (**2**) S27
Figure S22: Expansion (0.5–4.7 ppm) of the ^{1}H NMR spectrum of [DMAdda[5]]MC-LHar (**2**) S28
Figure S23: Expansion (4.7–9.5 ppm) of the ^{1}H NMR spectrum of [DMAdda[5]]MC-LHar (**2**) S29
Figure S24: DEPT135Q NMR spectrum of [DMAdda[5]]MC-LHar (**2**) S30

Figure S25: Expansion (10–60 ppm) of the DEPT135Q NMR spectrum of [DMAdda[5]]MC-LHar (**2**) S31
Figure S26: Expansion (70–190 ppm) of the DEPT135Q NMR spectrum of [DMAdda[5]]MC-LHar (**2**) S32
Figure S27: COSY NMR spectrum of [DMAdda[5]]MC-LHar (**2**) S33
Figure S28: DIPSI2 (80 ms) NMR spectrum of [DMAdda[5]]MC-LHar (**2**) S34
Figure S29: ROESY NMR spectrum of [DMAdda[5]]MC-LHar (**2**) S35
Figure S30: Edited HSQC135 NMR spectrum of [DMAdda[5]]MC-LHar (**2**) S36
Figure S31: HMBC NMR spectrum of [DMAdda[5]]MC-LHar (**2**) S37
Figure S32: PP2A Inhibition assay and Adda-ELISA curves... S38
Figure S33: Ion trap full-scan mass spectra (negative ionization; LC-MS) of oxidized standards of MC-LR (**9**), [DMAdda[5]]MC-LR (**1**) and [ADMAdda[5]]MC-LR (**3**) S39
Figure S34: Triple quadrupole LC–MS/MS chromatogram of all observed oxidation products S40
Figure S35: Triple quadrupole LC–MS/MS chromatograms and spectra from the peak with *m*/*z* 235. .... S41
Figure S36: Ion trap LC–MS/MS chromatogram and spectrum of MOMAPH.. S42
Figure S37: Triple quadrupole LC–MS/MS chromatogram and spectrum of MOMAPH..................................... S43
Figure S38: Ion trap negative ionization LC–MS/MS and –MS/MS/MS spectra of MOMAPH. S44
Figure S39: Ion trap LC–MS/MS chromatogram and spectrum (positive ionization) of MOMAPH...................... S45
Figure S40: Triple-quadrupole LC–MS/MS chromatogram and spectrum (positive ionization) of MOMAPH. S46
Figure S41: Semi-preparative HPLC–UV (254 nm) chromatogram of MOMAPH. S47
Figure S42: Ion trap LC–MS/MS chromatogram and spectrum of MHPB. S48
Figure S43: Triple-quadrupole LC–MS/MS chromatogram and spectrum of MHPB. S49
Figure S44: Triple-quadrupole LC–MS/MS chromatogram and spectra of MOMHPH... S50
Figure S45: Ion trap LC–MS/MS chromatogram and spectrum of MMPB.. S51
Figure S46: Triple-quadrupole LC–MS/MS chromatogram and spectrum of MMPB. S52
Figure S47: Triple-quadrupole LC–MS/MS chromatogram and spectrum of MOMMPH..................................... S53
Figure S48: LC–HRMS/MS spectra of MC-LA (**17**) and [DMAdda[5]]MC-LA S54

Table S1: Gradient conditions employed for semi-preparative HPLC for the purification of intact ADMAdda[5]]MCs (Methods 1−4) and [DMAdda[5]]MCs (Method 5). See Figures S1–S3, S5.

Method 1 - Initial HPLC of Nostoc

Luna C18 (5 µm; 150 × 10 mm)

Time (min)	2 mL min^{-1} 0.01% TFA	100% MeOH
0	40%	60%
10	40%	60%
25	10%	90%
35	40%	60%
40	40%	60%

Method 2 - [ADMAdda[5]]MCs (**3**, **4**)

Luna C18 (5 µm; 150 × 10 mm)

Time (min)	2 mL min^{-1} 0.01% TFA	100% MeOH
0	40%	60%
5	40%	60%
15	10%	90%
25	40%	60%
30	40%	60%

Method 3 - [ADMAdda[5]]MCs (**5**, **6**)

NovaPak (4 µm; 250 × 4.6 mm)

Time (min)	1 mL min^{-1} 5 mM $(NH_4)_2CO_3$	Acetonitrile
0	80%	20%
12	80%	20%
35	50%	50%
40	80%	20%
45	80%	20%

Method 4 - [ADMAdda[5]]MCs (**3**, **4**)

NovaPak (4 µm; 250 × 4.6 mm)

Time (min)	1 mL min^{-1} 5 mM $(NH_4)_2CO_3$	Acetonitrile
0	90%	10%
10	80%	20%
25	60%	40%
30	90%	10%
35	90%	10%

Method 5 - [DMAdda[5]]MCs (**1**, **2**)

NovaPak (4 µm; 250 × 4.6 mm)

Time (min)	1 mL min^{-1} 5 mM $(NH_4)_2CO_3$	Acetonitrile
0	90%	10%
5	90%	10%
25	80%	20%
35	60%	40%
40	40%	60%
45	90%	10%
50	90%	10%

Table S2: MRM transitions monitored for intact congeners of MC and NOD-R (IS = internal standard).

Analyte	ID	Precursor Ion (*m*/*z*)		Product Ions (*m*/*z*)	CE%
[DMAdda[5]]MC-LR	**1**	$[M+H]^+$	981.5	539.3, 553.4, 585.4, 599.4, 852.6, 953.6, 963.6	14%
[DMAdda[5]]MC-LHar	**2**	$[M+H]^+$	995.5	375.2, 553.4, 599.4, 866.6, 875.6, 967.6, 977.6	18%
[D-Asp[3]]MC-RR	**21**	$[M+2H]^{2+}$	512.9	291.3, 426.3, 445.8, 503.8	28%
MC-RR	**12**	$[M+2H]^{2+}$	519.9	298.3, 440.3, 452.8, 455.3, 503.8	20%
NOD-R	**–**	$[M+H]^+$	825.5	389.4, 674.5, 691.5, 753.5, 781.5, 808.0	24%
MC-YR	**13**	$[M+H]^+$	1045.5	440.3, 599.4, 602.4, 1017.5, 1027.5	20%
MC-HtyR	**14**	$[M+H]^+$	1059.5	599.4, 634.4, 1031.6, 1041.6	16%
MC-LR	**9**	$[M+H]^+$	995.5	375.2, 553.4, 599.4, 866.6, 875.6, 967.6, 977.6	18%
[D-Asp[3]]MC-LR	**7**	$[M+H]^+$	981.5	539.3, 553.4, 585.4, 599.4, 852.6, 953.6, 963.6	14%
[Dha[7]]MC-LR	**8**	$[M+H]^+$	981.5	539.3, 553.4, 585.4, 599.4, 852.6, 953.6, 963.6	14%
[ADMAdda[5]]MC-LR	**3**	$[M+H]^+$	1023.5	553.4, 627.5, 641.4, 738.8, 894.6, 963.6, 995.6, 1005.6	18%
[D-Asp[3],ADMAdda[5]]MC-LR	**5**	$[M+H]^+$	1009.5	567.4, 599.5, 627.4, 981.5, 992.6	20%
MC-HilR	**10**	$[M+H]^+$	1009.5	567.4, 599.5, 627.4, 981.5, 992.6	20%
[ADMAdda[5]]MC-LHar	**4**	$[M+H]^+$	1037.6	599.5, 613.4, 641.5, 908.6, 977.6, 1009.5, 1019.6	21%
MC-WR	**15**	$[M+H]^+$	1068.6	599.4, 626.3, 939.5, 1040.6	21%
[D-Asp[3],ADMAdda[5]]MC-LHar	**6**	$[M+H]^+$	1023.6	553.4, 627.4, 641.4, 738.5, 894.6, 963.6, 995.6, 1005.6	18%
[D-Leu[1]]MC-LR	**11**	$[M+H]^+$	1037.6	599.5, 613.4, 641.5, 908.6, 977.6, 1009.5, 1019.6	21%
MC-RY	**16**	$[M-H]^-$	1043.5	1001.6, 1025.6	20%
MC-LA	**17**	$[M-H]^-$	908.5	780.0, 797.0, 878.0, 891.0	20%
MC-LY	**18**	$[M-H]^-$	1000.5	872.0, 889.0, 970.0, 983.0	20%
MC-LW	**20**	$[M-H]^-$	1023.6	1005.6	20%
MC-LF	**19**	$[M-H]^-$	984.6	966.6	20%
d_7-MC-LR (IS)	**–**	$[M+H]^+$	1002.5	599.54	20%
d_5-MC-LF (IS)	**–**	$[M-H]^-$	984.6	966.6	20%

Table S3: Adda-ELISA data (concentration and absorbance values (ABS)) for MC-LR (**9**), [DMAdda[5]]MCs (**1**, **2**) and [ADMAdda[5]]MCs (**3**–**6**). IC_{50} values (ng mL^{-1}) with standard errors in parentheses are included.

Analyte	IC_{50}	Conc (ng mL^{-1})	ABS1	ABS2
Water	NA	0	2.031	2.092
		0	2.073	2.153
		0	2.063	2.182
MC-LR (**9**)	0.48 (0.08)	0.2	1.697	1.705
		0.6	1.252	1.242
		1	1.018	1.012
		2.5	0.729	0.737
		4	0.602	0.612
[ADMAdda[5]]MC-LR (**3**)	> 200	50	1.852	1.829
		150	1.612	1.522
		250	1.507	1.574
		625	1.362	1.334
		1000	1.139	1.134
[ADMAdda[5]]MC-LHar (**4**)	> 200	50	1.759	1.713
		150	1.63	1.626
		250	1.429	1.429
		625	1.317	1.278
		1000	1.106	1.085
[D-Asp[3],ADMAdda[5]]MC-LR (**5**)	> 200	50	1.471	1.563
		150	1.36	1.354
		250	1.253	1.208
		625	1.038	1.024
		1000	0.957	0.925
[D-Asp[3],ADMAdda[5]]MC-LHar (**6**)	> 200	50	1.609	1.591
		150	1.381	1.273
		250	1.228	1.248
		625	0.984	0.937
		1000	0.885	0.742
[DMAdda[5]]MC-LR (**1**)	> 200	50	1.525	1.59
		150	1.29	1.227
		250	1.247	1.227
		625	1.058	0.975
		1000	1.014	0.997
[DMAdda[5]]MC-LHar (**2**)	> 200	50	1.611	1.606
		150	1.329	1.31
		250	1.248	1.263
		625	0.956	0.936
		1000	0.897	0.87

Table S4: PP2A Inhibition Assay data (concentration and absorbance values (ABS)) for MC-LR (**9**), [DMAdda^5]MCs (**1**, **2**) and [ADMAdda^5]MCs (**3**–**6**). IC_{50} values (ng mL^{-1}) with standard errors in parentheses are included.

Analyte	IC_{50}	Conc (ng mL^{-1})	ABS1	ABS2
Water	NA	0.00	1.938	1.952
		0.00	2.108	2.115
MC-LR (**9**)	0.42 (0.03)	0.25	1.577	1.602
		0.50	1.165	1.15
		1.00	0.696	0.698
		2.50	0.534	0.548
[ADMAdda^5]MC-LR (**3**)	0.42 (0.03)	0.25	1.632	1.679
		0.50	1.236	1.141
		1.00	0.741	0.729
		2.50	0.602	0.612
[ADMAdda^5]MC-LHar (**4**)	0.50 (0.04)	0.25	1.686	1.696
		0.50	1.294	1.293
		1.00	0.786	0.78
		2.50	0.575	0.588
[D-Asp^3,ADMAdda^5]MC-LR (**5**)	0.37 (0.03)	0.25	1.528	1.52
		0.50	1.152	1.145
		1.00	0.781	0.767
		2.50	0.642	0.654
[D-Asp^3,ADMAdda^5]MC-LHar (**6**)	0.43 (0.04)	0.25	1.599	1.589
		0.50	1.234	1.243
		1.00	0.797	0.761
		2.50	0.643	0.647
[DMAdda^5]MC-LR (**1**)	0.52 (0.06)	0.25	1.632	1.683
		0.50	1.345	1.352
		1.00	0.833	0.824
		2.50	0.632	0.615
[DMAdda^5]MC-LHar (**2**)	0.41 (0.04)	0.25	1.522	1.55
		0.50	1.168	1.175
		1.00	0.709	0.707
		2.50	0.569	0.562

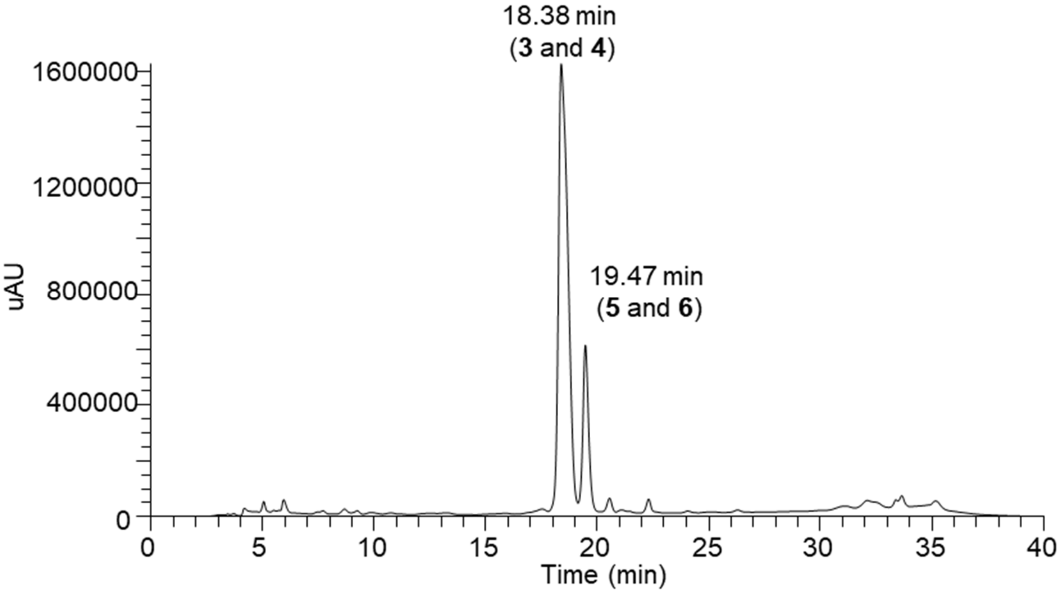

Figure S1: Method 1; Semi-preparative HPLC–UV chromatogram (238 nm) showing the initial injections of *Nostoc* 152 extracts. Peaks each correspond to the fraction collections made, which contained two variants each (18.38 min = **3**, **4**; 19.47 min = **5**, **6**) requiring additional HPLC purification.

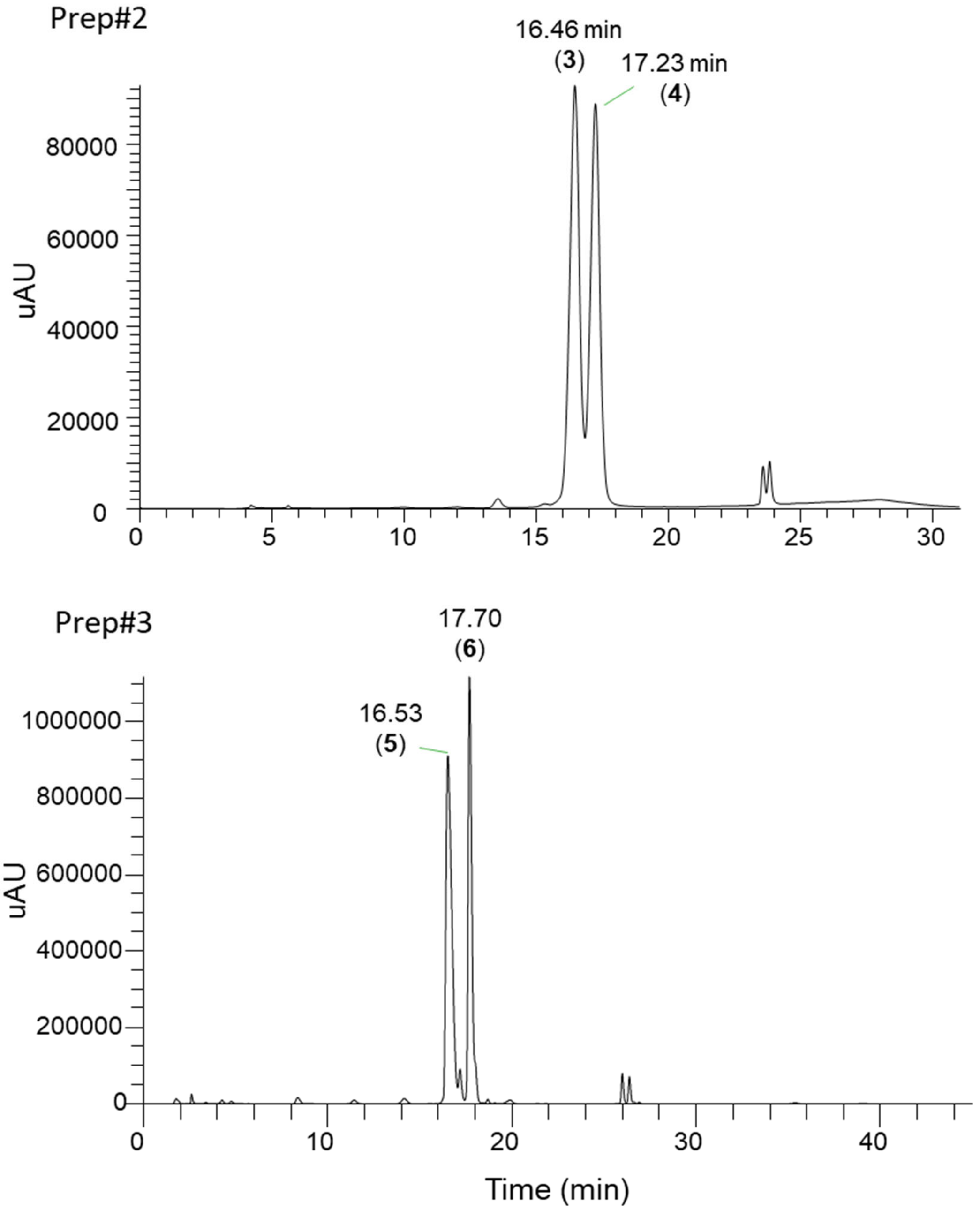

Figure S2: Semi-preparative HPLC–UV chromatogram (238 nm) used to separate: top (method 2), [ADMAdda^{5}]MC-LR (**3**) from [ADMAdda^{5}]MC-LHar (**4**), and; bottom (method 3) [D-Asp^{3},ADMAdda^{5}]MC-LR (**5**) from [D-Asp^{3},ADMAdda^{5}]MC-LHar (**6**).

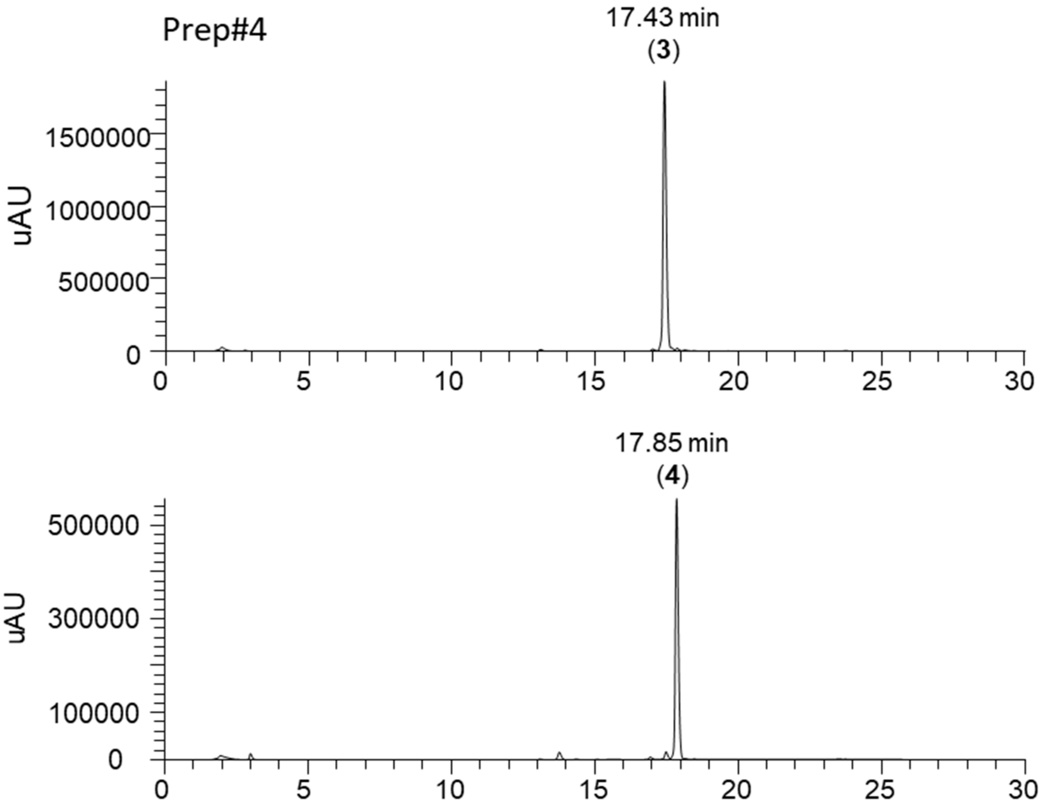

Figure S3: Semi-preparative HPLC–UV chromatograms (238 nm) (method 4) for the final purification of [ADMAdda^5]MC-LR (**3**) and [ADMAdda^5]MC-LHar (**4**).

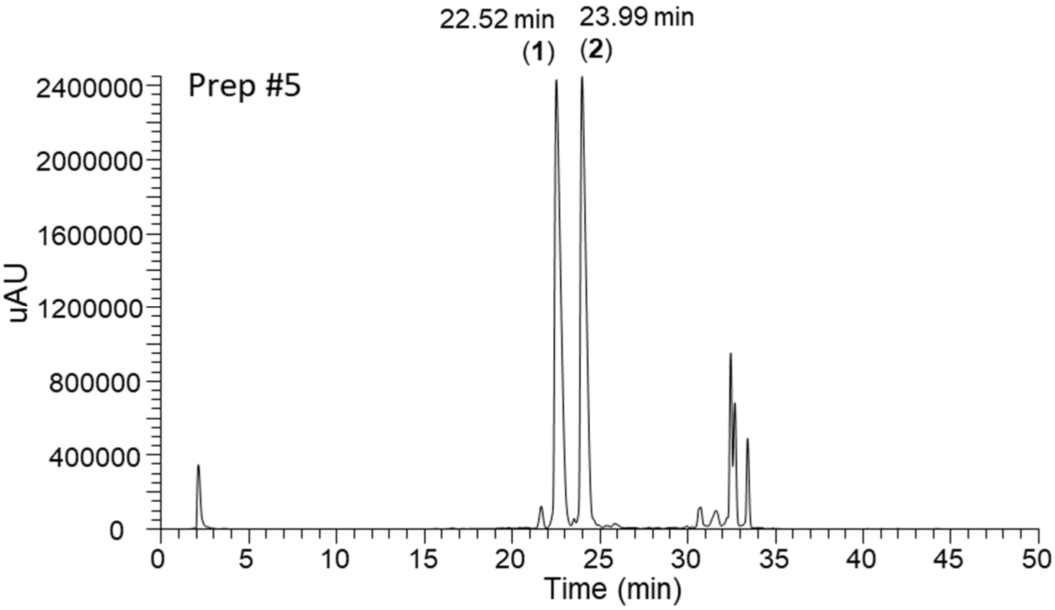

Figure S4: Semi-preparative HPLC–UV chromatogram (238 nm) (method 5) of the *Nostoc* sp. strain 152 fraction-A (Figure S1) after hydrolysis, showing separation of [DMAdda^5]MC-LR (**1**) and [DMAdda^5]MC-LHar (**2**).

E. Calculations and Graphic Representation of Results

****Note: A worksheet to calculate the results is available from Eurofins Abraxis free of charge. Please, contact us for further information.

1 Obtain a standard curve by plotting standards absorbance at 405 nm in the *y* axis and concentration of Microcystin-LR in a logarithmic *x* axis. Draw a standard curve. An example of standard curve is shown below:

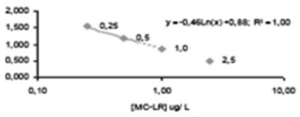

2 The concentration of microcystins in the sample is calculated by interpolating the calibration curve or using the following equation:

$$y = a\,Lnx + b \qquad x = EXP(y\text{-}b/a)$$

Where "*x*" value is concentration of microcystin-LR equivalents in the sample and the "*y*" the absorbance at 405 nm.

F. REFERENCES

1. An, J., and W.W. Carmichael. 1994. Use of a colorimetric protein phosphatase assay and enzyme linked immunoassay for the study of microcystins and nodularins. Toxicon. 1994 Dec;32(12):1495-507.
2. McElhiney, J., and Lawton, L.A. Detection of the cyanobacterial hepatotoxins microcystins. Toxicol Appl Pharmacol. 2005 Mar 15;203(3):219-30.
3. WHO (1998) Guidelines for Drinking-Water Quality. Second ed. Addendum to Vol. 1. World Health Organization, Geneva.
4. Bouaïcha N, Maatouk I, Vincent G, Levi Y. A colorimetric and fluorometric microplate assay for the detection of microcystin-LR in drinking water without preconcentration. Food Chem Toxicol. 2002 Nov;40(11):1677-83.

General Limited Warranty/Disclaimer: Eurofins Abraxis warrants the products manufactured by the Company, against defects and workmanship when used in accordance with the applicable instructions for a period not to extend beyond the product's printed expiration date. Eurofins Abraxis makes no other warranty, expressed or implied. There is no warranty of merchantability or fitness for a particular purpose.

For ordering or technical assistance contact:

Eurofins Abraxis
124 Railroad Drive
Warminster, PA 18974
Tel.: (215) 357-3911
Fax: (215) 357-5232
Email: info.ET.Warminster@eurofinsus.com
WEB: www.abraxiskits.com

Some components are manufactured by Zeu-Inmunotec, S. L.: ZEU

080819

eurofins | Abraxis

Microcystins/Nodularins PP2A, Microtiter Plate
Test for the Detection of Microcystins and Nodularins In Water

Product No. 520032

1. General Description

Microcystins/Nodularins PP2A Kit is an enzymatic test for the detection of microcystins and nodularins in water. A simple and rapid method that allows to quantify whether the toxin concentration is over the maximum allowed levels (1 µg/L, OMS 1998).

2. Safety Instructions

The standard solutions in this test kit contain small amounts of Microcystins in solution. Avoid contact of standard and stopping solutions with skin and mucous membranes. If these reagents come in contact with the skin, wash with water. Recommended: Polypropylene material should be avoided throughout sample collection, conservation and treatment, since loss of toxins has been shown to occur.

3. Storage and Stability

The Microcystins/Nodularins PP2A Kit should to be stored in the refrigerator (4–8°C) prior to use and protected from light. The solutions must be allowed to reach room temperature (20-25°C) before use. Reagents may be used until the expiration date on the box.

4. Test Principle

Microcystins/Nodularins PP2A Kit is based on the phosphatase activity inhibition by microcystins. Under normal conditions the phophatase is able to hydrolyse a specific substrate that can be detected at 405 nm. Samples containing microcystins will inhibit the enzyme activity proportionally to the amount of toxin contained in the sample. The concentration of the toxin in the sample can be calculated using a standard curve.

5. Limitations of the Microcystins ELISA, Possible Test Interference

Numerous organic and inorganic compounds commonly found in water samples have been tested and found not to interfere with this test. However, due to the high variability of compounds that might be found in water samples, test interferences caused by matrix effects can't be completely excluded.

Mistakes in handling the test can also cause errors. Possible sources for such errors can be:

Inadequate storage conditions of the test kit, wrong pipetting sequence or inaccurate volumes of the reagents, too long or too short incubation times during the immune and/or substrate reaction, extreme temperatures during the test performance (lower than 10°C or higher than 30°C). The assay procedure should be performed away from direct sun light.

As with any analytical technique (GC, HPLC, etc.) positive results requiring some action should be confirmed by an alternative method.

Working Instuctions

A. Materials Provided

1. Microtiter plate
2. 4 vials of Phosphatase
3. Standards Microcystins (4): 0.25, 0.50, 1.00, 2.50 ppb
4. 1 vial Chromogenic Substrate
5. 1 vial Phosphatase Dilution Buffer
6. 1 vial Stop Soultion

B. Test Preparation

Micro-pipetting equipment and pipette tips for pipetting the standards and the samples are necessary. We recommend using a multi-channel pipette or a stepping pipette for adding the assay buffer, substrate and stop solutions in order to equalize the incubations periods of the solutions on the entire microtiter plate. Please use only the reagents and standards from one package lot in one test, as they have been adjusted in combination.

SOLUTIONS

All reagents must be allowed to reach room temperatura (23 ± 3°C) before starting the assay.

1. Phophatase Solution: Add 3 mL of Phosphatase Dilution Buffer to one of the Phosphatase vials and mix carefully by inversion. Gently shake the solution at room temperature (23 ± 3 °C) for 60 minutes (or manually several times during that period) to ensure that the enzyme is fully hydrated. This solution must be stored under refrigeration if not used immediately after dissolution. Do not use the Phosphatase Solution for following days.

Each enzyme vial contains enough volume of phosphatase for 24 wells. If more than one vial is needed, dissolve each vial as described above, make a pool with the content of those vials and mix gently by inversion before use.

Attention: this reagent is blue and becomes brownish when dissolved. If the phosphatase turns to brownish colour before the hydratation, please discard as this reagent could be damaged.

2. Standards: the standards are ready to use. They are provided in vials containing a total volume of 1.2 mL.

C. Assay Procedure

1. Add 50 µL of each Microcystin-LR standard in duplicate (i. e.: wells A1 and A2, 0.25 µg/L; wells B1 and B2, 0.50 µg/L; wells C1 and C2, 1.00 µg/L; wells D1 and D2, 2.50 µg/L). We recommend using duplicates or triplicates.

2. Add 50 µL of each sample in duplicate into the remaining wells of the microtiter plate.

3. Add 70 µL of the Phosphatase Solution to each well.

4. Add 90 µL of Chromogenic Substrate to each well and mix gently. The substrate contains solid in suspension. Do not mix the reagent prior to use and avoid taking any solid.

5. Put the adhesive film on wells and Incubate the plate for 30 minutes at 37 °C.

6. Add 70 µL of Stop Solution to each well. Mix gently.

7. Read the absorbance of samples and standards at 405 nm. Use an empty well as blank, if necessary.

D. Additional Materials (not included with the test kit)

1. Micro-pipettes with disposable plastic tips (10-200 and 200-1000 µL)
2. Multi-channel pipette (50-250 µL) or stepper pipette with plastic tips (10-250 µL)
3. Microtiter plate reader (wave length 405 nm)
4. Timer
5. Tape or Parafilm
6. Glass vials with Teflon-lined caps
7. Distilled or deionized water
8. Vortex mixer
9. Heater at 37 +/- 2 °C

E. Sample Preparation

1. Drinking water (water treated in drinking water stations):

Sample preparation is not required. Following the procedure described in section C (**Assay Procedure**) the content of dissolved microcystins will be determined. Please, pay attention to the note mentioned below.

2. Water from reservoirs, rivers, etc

The content of dissolved microcystins, intracellular microcystins and total microcystins can be determined (see Scheme below). Please, pay attention to the note mentioned below.

2.1 <u>Dissolved microcystins:</u> Sample preparation is not required. Following the procedure described in section C, dissolved microcystins content will be determined.

*****If any interferences are suspected (i.e. high concentration of heavy metals, turbidity), please contact Eurofins Abraxis for technical assistance.*****

2.2 <u>Intracellular microcystins:</u>

a) Take 200 mL of sample and filer in vacuum through a 0.8 µm nylon membrane (i.e. Whatman Nylon Membrane Filters, ref.: 7408-004). Reserve the filtrate for further determination of total microcystins, as it is explained in paragraph 2.3.

b) Take the membrane with the residue and place in a glass flask. The membrane can be cut into pieces to improve the extraction step.

c) Add 10 mL of 80% MeOH in water with 0.1% TFA and 0.1% Tween 20. Incubate at room temperature for 30 minutes with gentle stirring and in absence of light.

d) Centrifuge at 4000 g for 5 min.

e) Take the supernatant and dilute it 20 times (dilution 1/20) with distilled water. At this point, the sample is ready to continue the assay as is shown in Section C. This way, the content of intracellular microcystins is determined. If the concentration of MC-LR equivalents exceeds 2.5 µg/L, we advice to perform the assay on a range of supernatant dilutions of 1/20, 1/200, 1/2000,...

2.3 <u>Total microcystins:</u>

Use the filtrate obtained in 2.2.a. and perform the assay described in **section C**. Total microcystins contained in the sample are calculated by adding the concentration of microcystins found in the filtrate plus the intracellular microcystins (2.2.e).

NOTE: Presence of thiosulphate (or strong oxidizing reagents) may interfere in the assay, therefore collecting samples in bottles with this chemical or adding it to the simple prior to testing should be avoided.

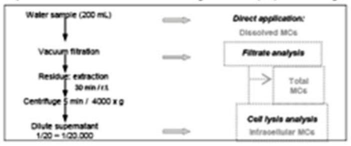

Scheme procedure for water samples from reservoirs, rivers, etc.

Figure S5: Manufacturer Instructions for PP2A Inhibition Assay (PN 520032). Sections 2.1 and C were followed.

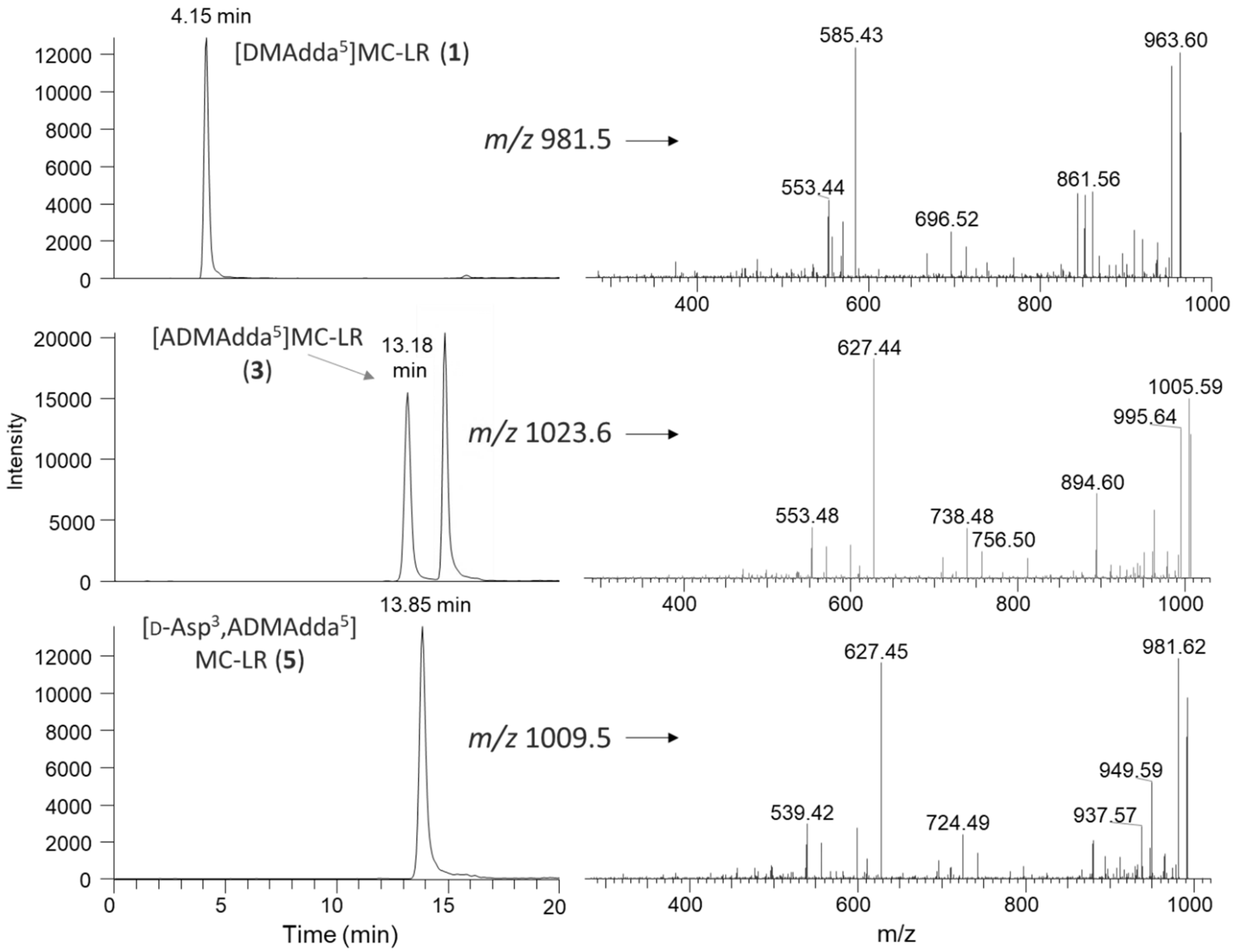

Figure S6: Ion trap LC–MS/MS chromatograms (left) and their corresponding MS/MS spectra (right) of the MC-LR variants purified from *Nostoc* sp. strain 152. From top to bottom, the congeners are [DMAdda^{5}]MC-LR (**1**), [ADMAdda^{5}]MC-LR (**3**) and [D-Asp^{3},DMAdda^{5}]MC-LR (**5**).

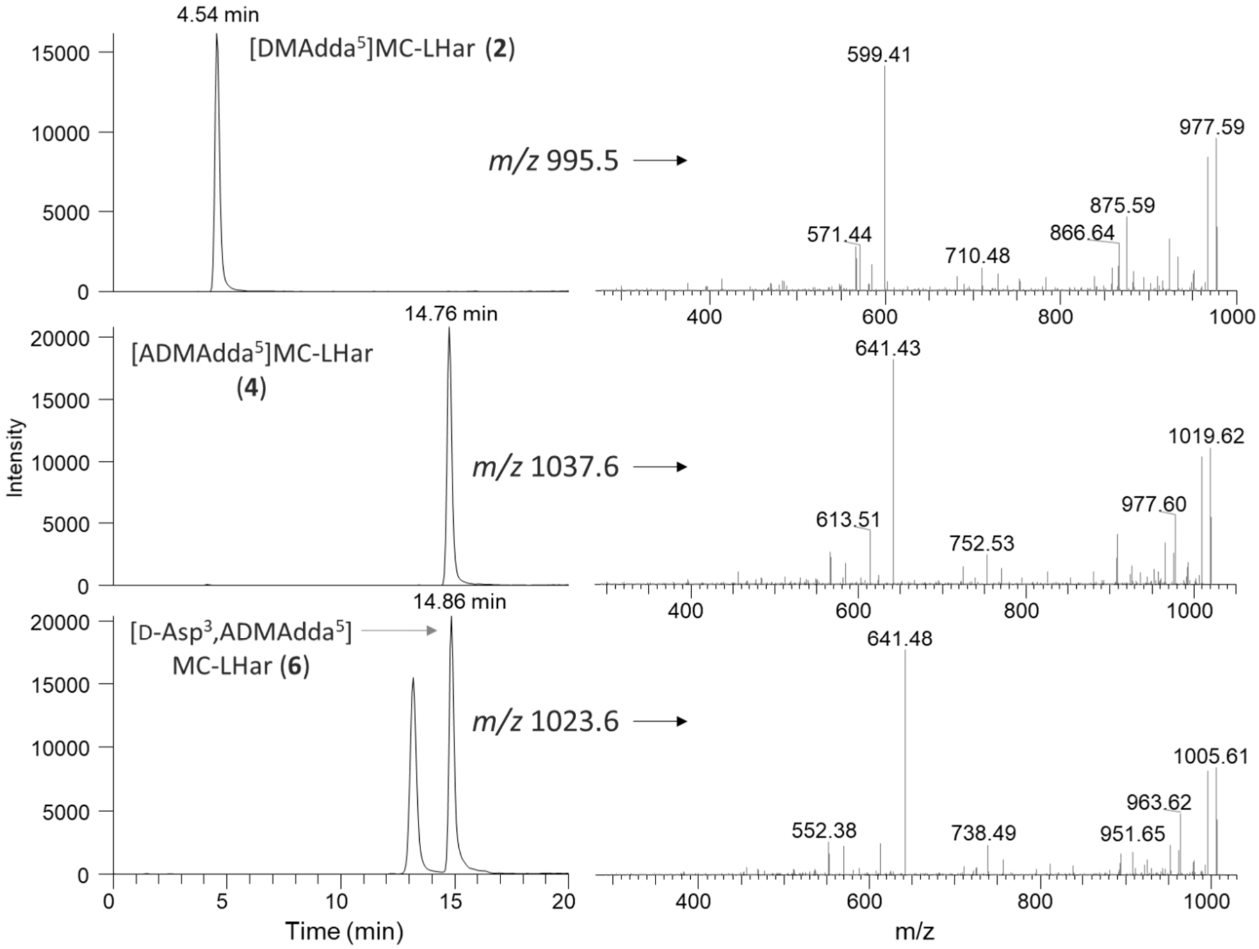

Figure S7: Ion trap LC–MS/MS chromatograms (left) and their corresponding MS/MS spectra (right) of the MC-LHar variants purified from *Nostoc* sp. strain 152. From top to bottom, the congeners are [DMAdda^5]MC-LHar (**2**), [ADMAdda^5]MC-LHar (**4**) and [D-Asp^3,DMAdda^5]MC-LHar (**6**).

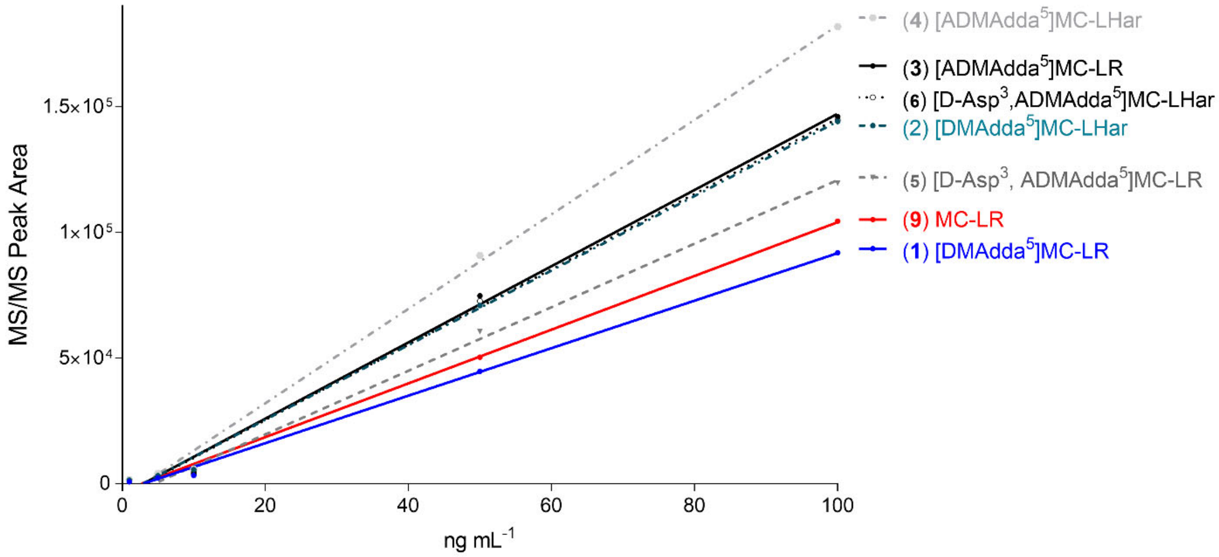

Figure S8: Ion Trap LC–MS/MS calibration curves for intact MCs (5-point; 1, 5, 10, 50 100 ng mL^{-1}) using the MRM transitions and quantitation ions (in bold) from Table 1.

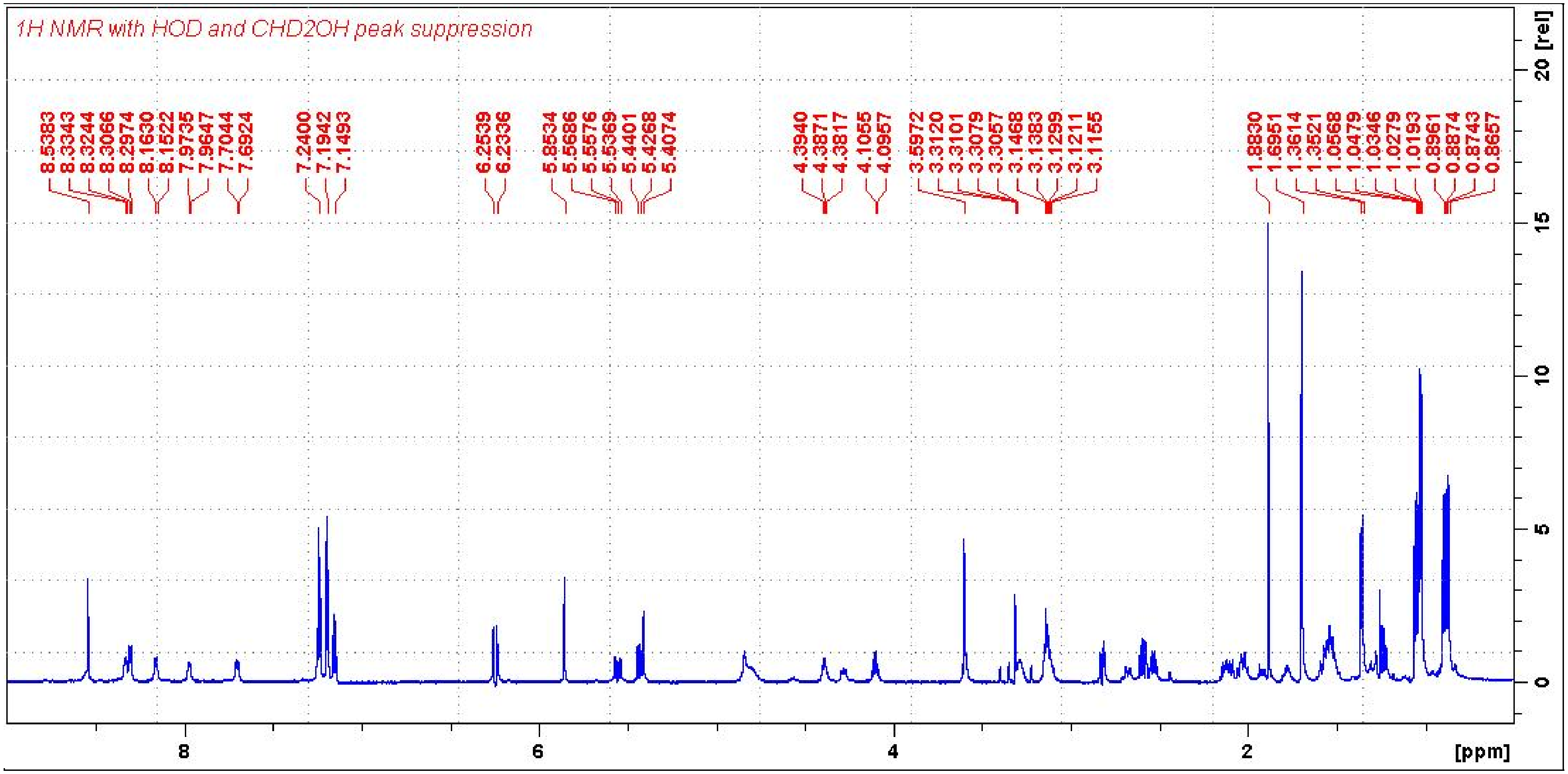

Figure S9: ^{1}H NMR spectrum of [DMAdda^5]MC-LR (**1**) in CD_3OH.

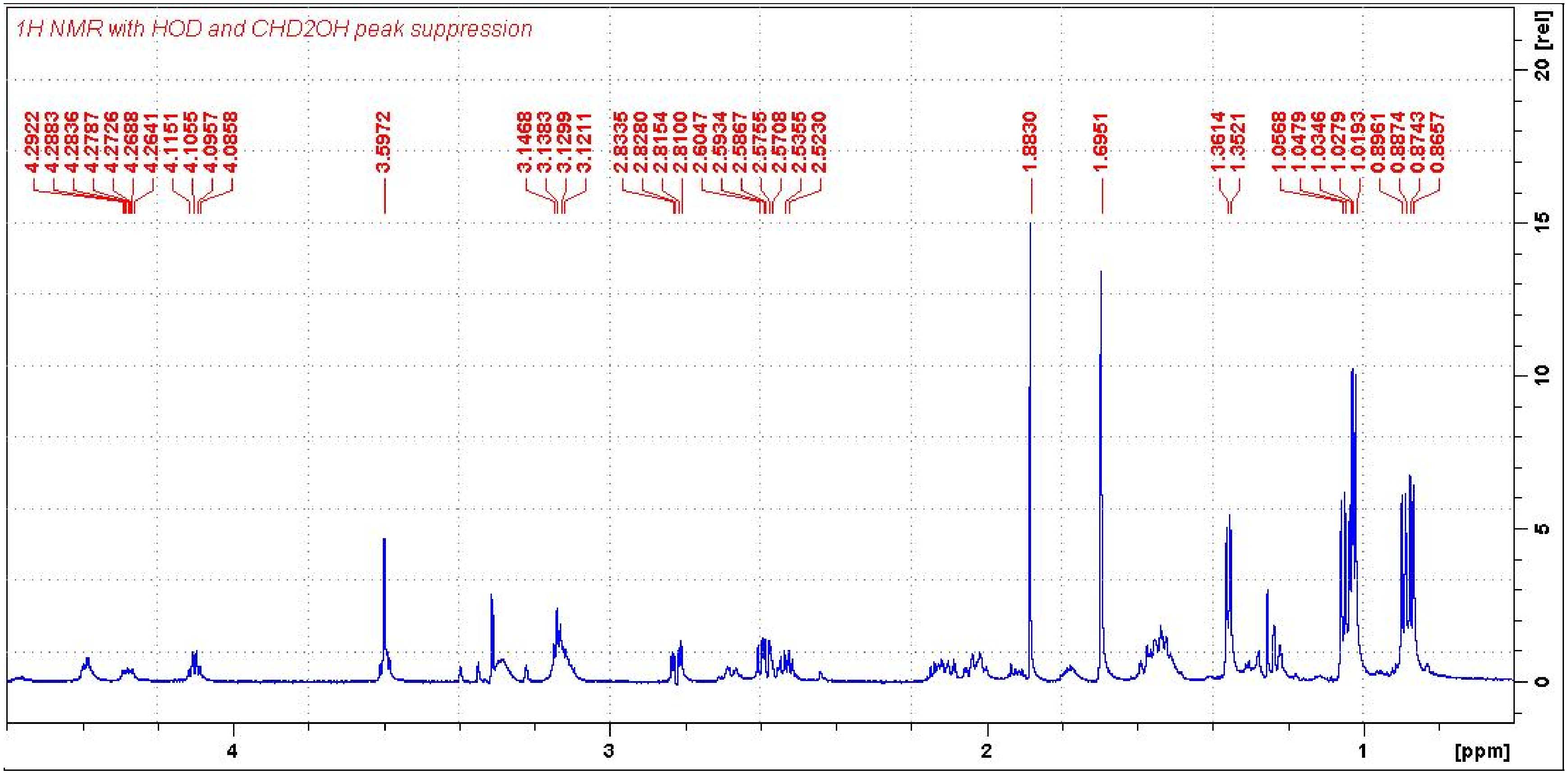

Figure S10: Expansion (0.6–4.6 ppm) of the ^1H NMR spectrum of [DMAdda^5]MC-LR (**1**) in CD_3OH.

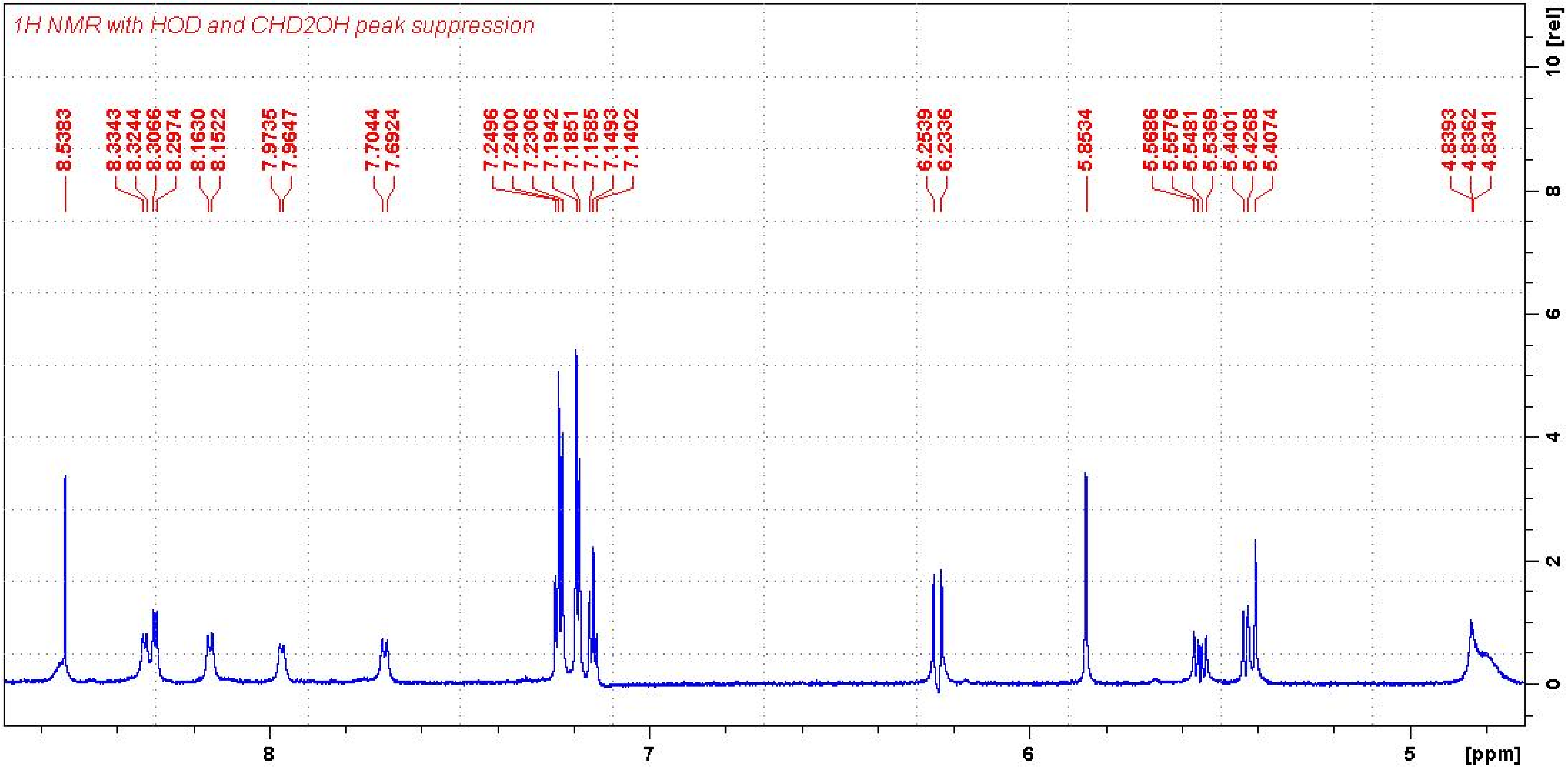

Figure S11: Expansion (4.7–8.7 ppm) of the ^{1}H NMR spectrum of [DMAdda[5]]MC-LR (**1**) in CD_3OH.

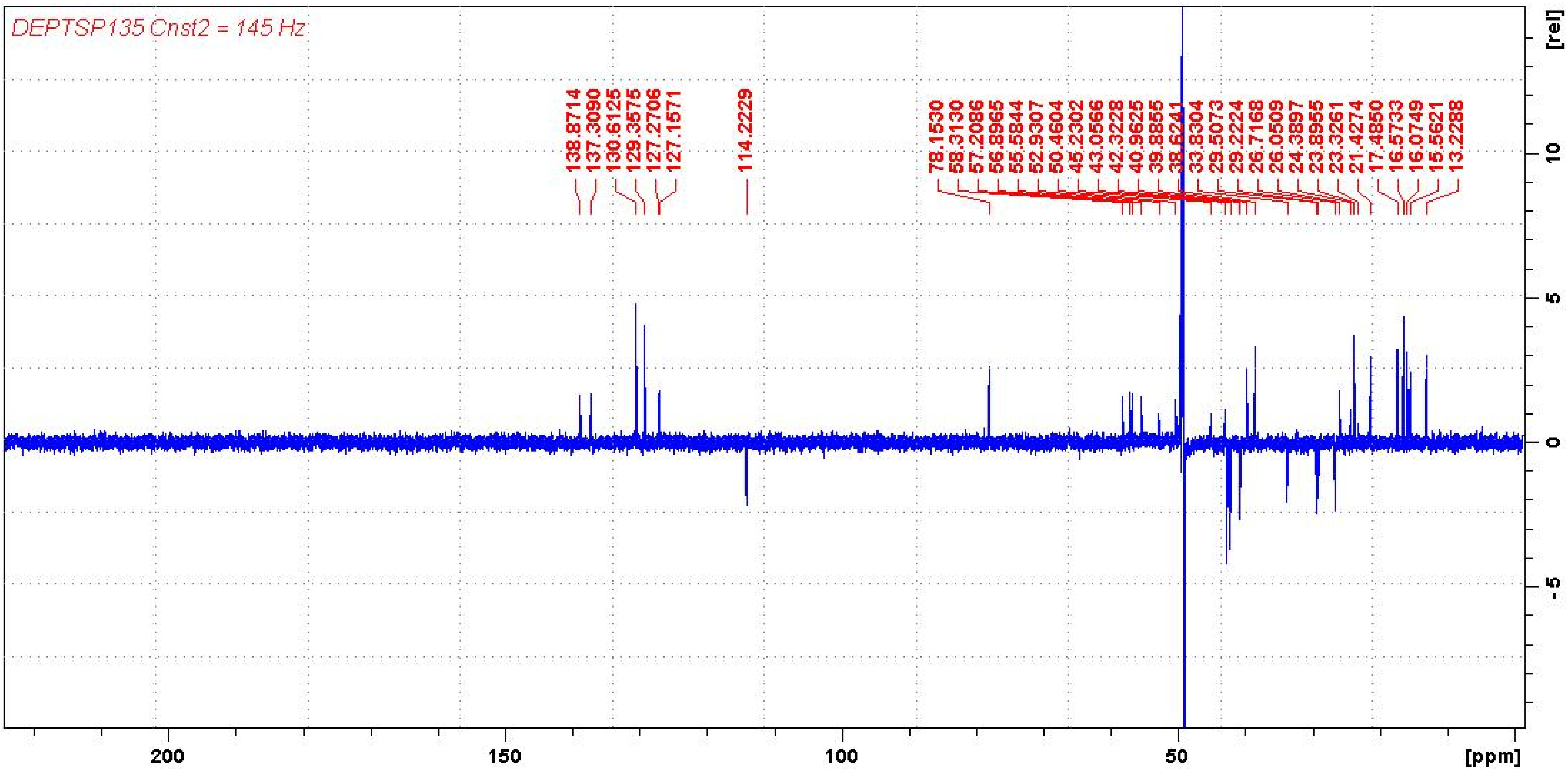

Figure S12: DEPT135 NMR spectrum of [DMAdda[5]]MC-LR (**1**) in CD_3OH.

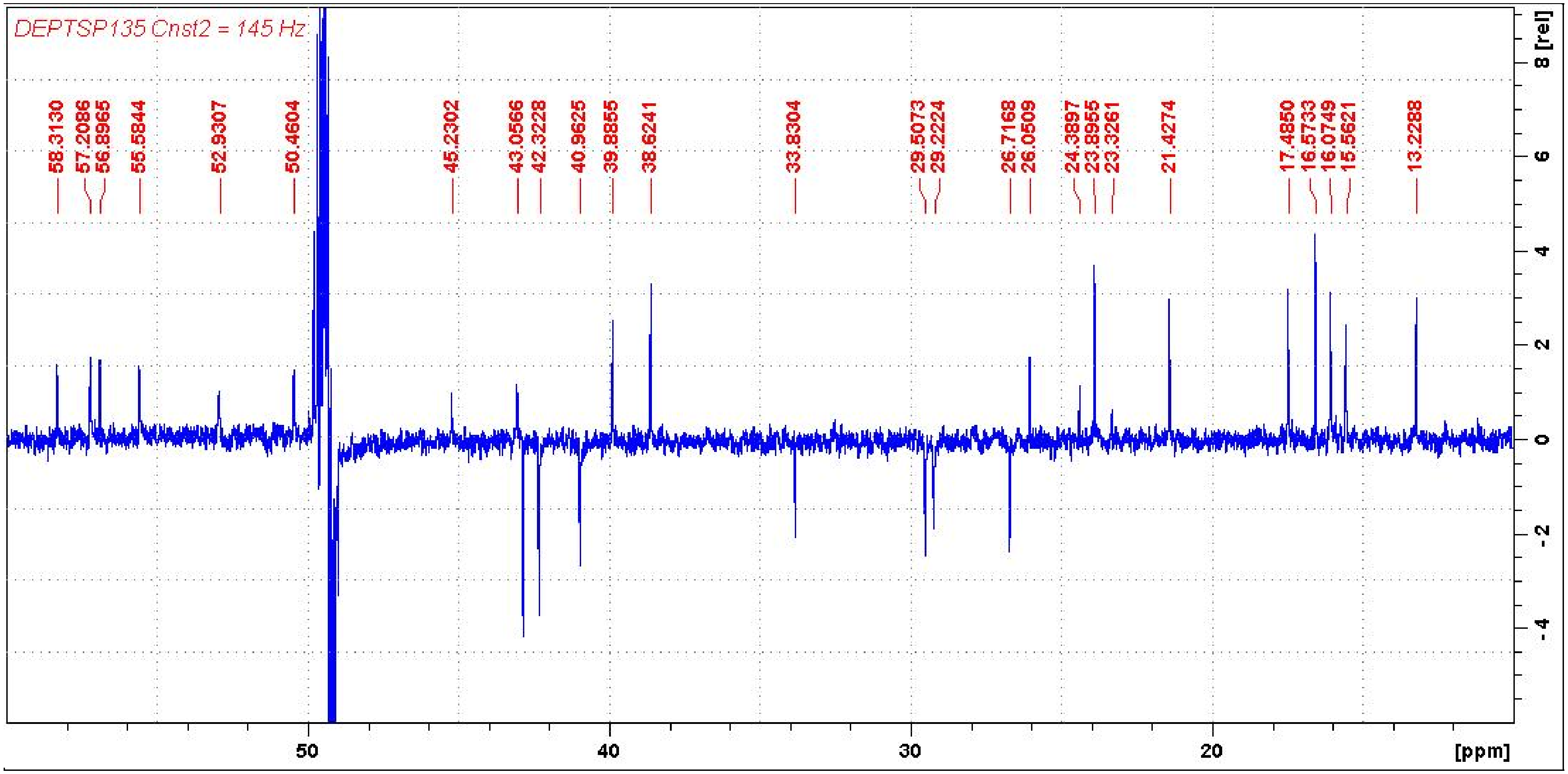

Figure S13: Expansion (10–60 ppm) of the DEPT135 NMR spectrum of [DMAdda⁵]MC-LR (**1**) in CD_3OH.

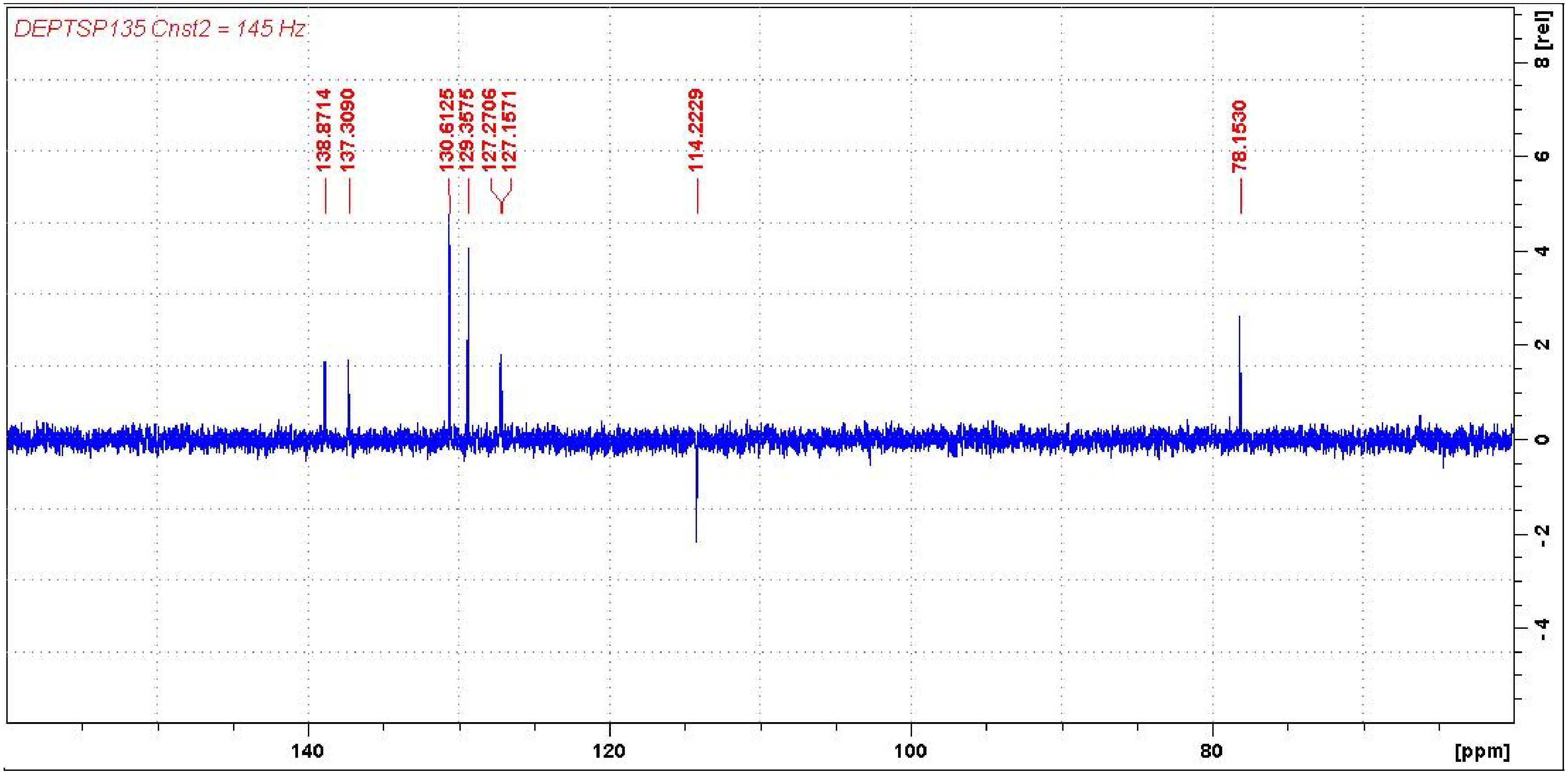

Figure S14: Expansion (60–160 ppm) of the DEPT135 NMR spectrum of [DMAdda[5]]MC-LR (**1**) in CD_3OH.

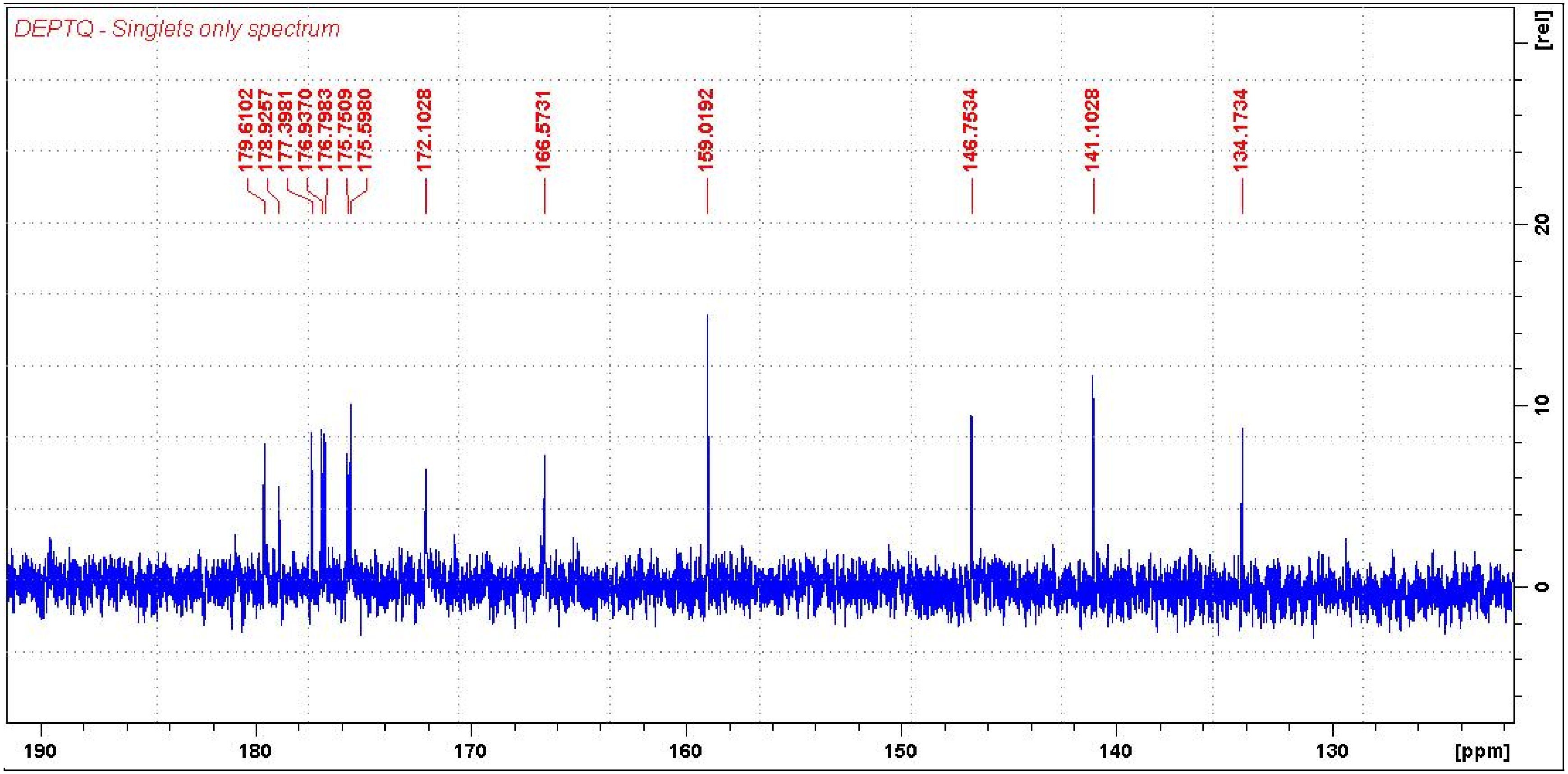

Figure S15: DEPTQ NMR spectrum of [DMAdda^5]MC-LR (**1**) in CD_3OH.

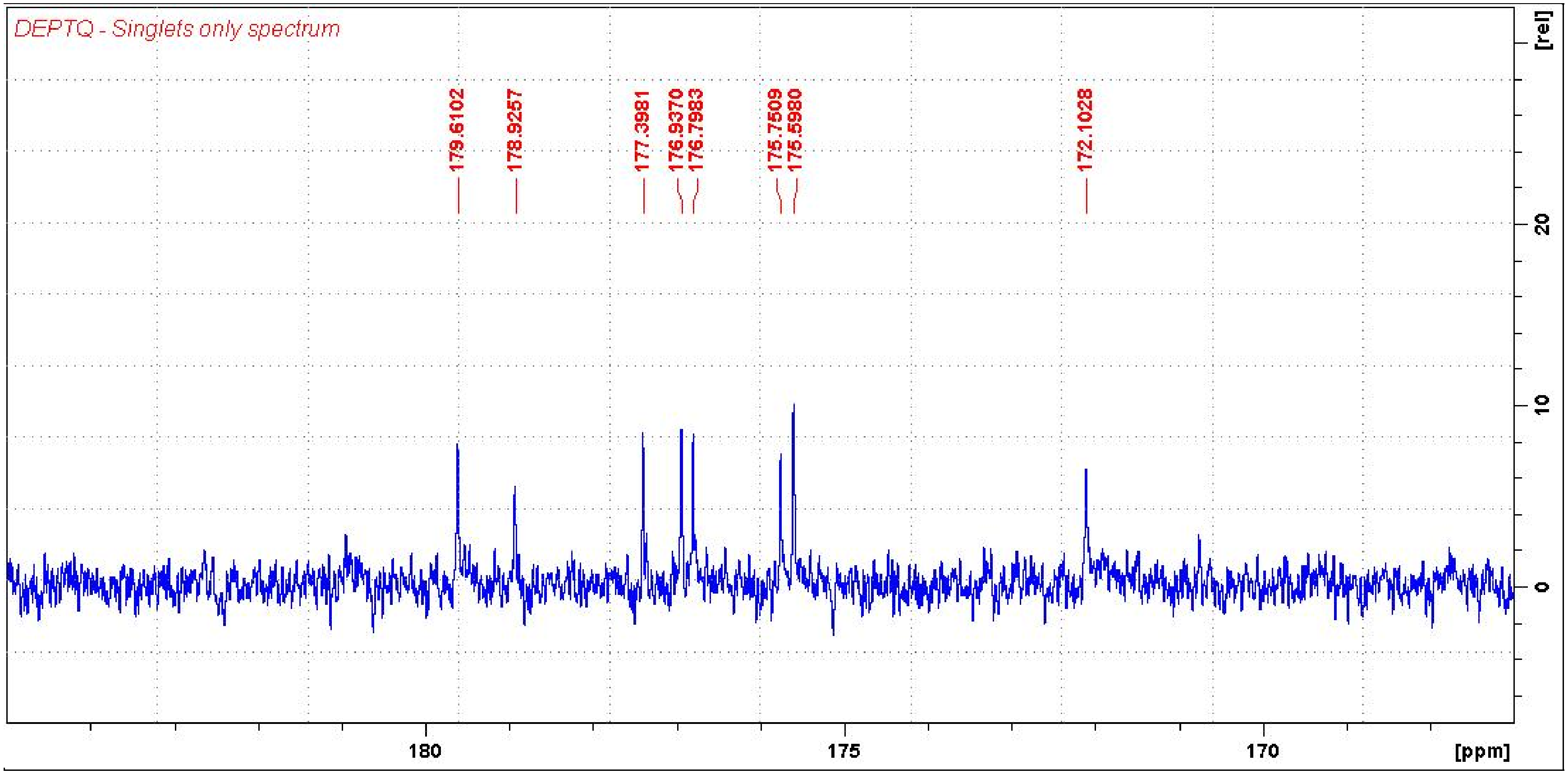

Figure S16: Expansion (167–185 ppm) of the DEPTQ NMR spectrum of [DMAdda^{5}]MC-LR (**1**) in CD_3OH.

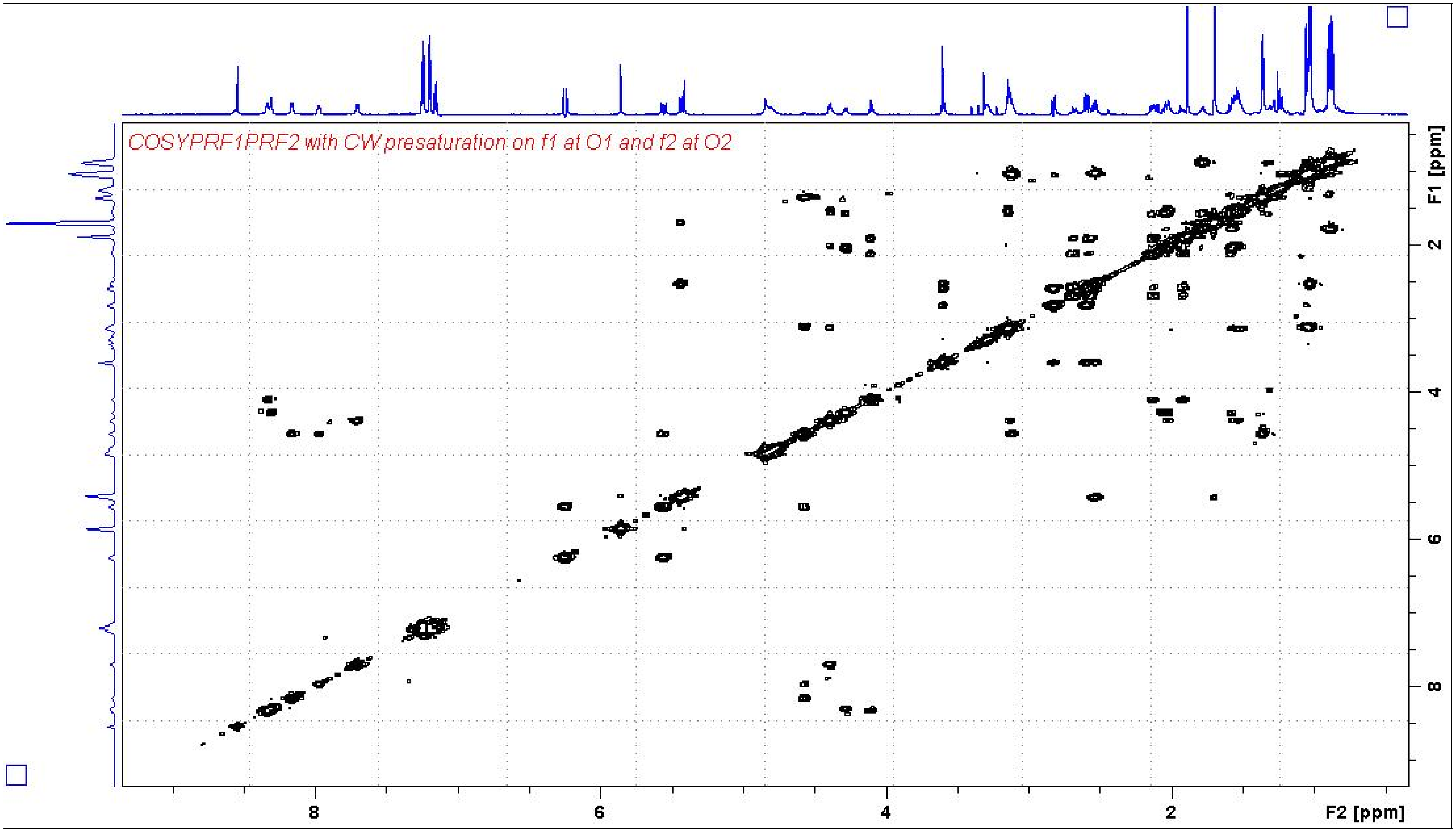

Figure S17: COSY NMR spectrum of [DMAdda[5]]MC-LR (**1**) in CD_3OH.

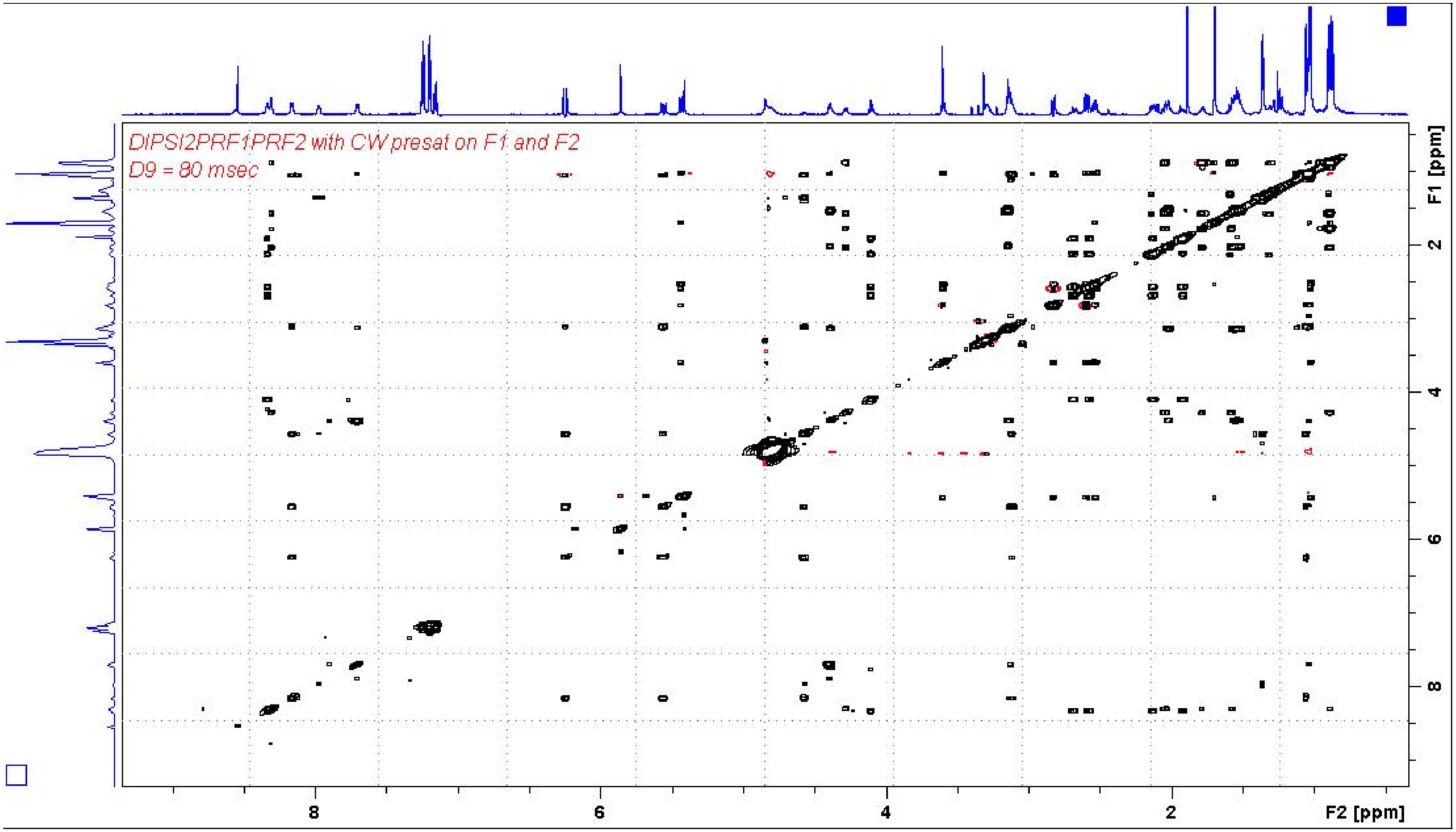

Figure S18: DIPSI2 (80 ms) NMR spectrum of [DMAdda[5]]MC-LR (**1**) in CD_3OH.

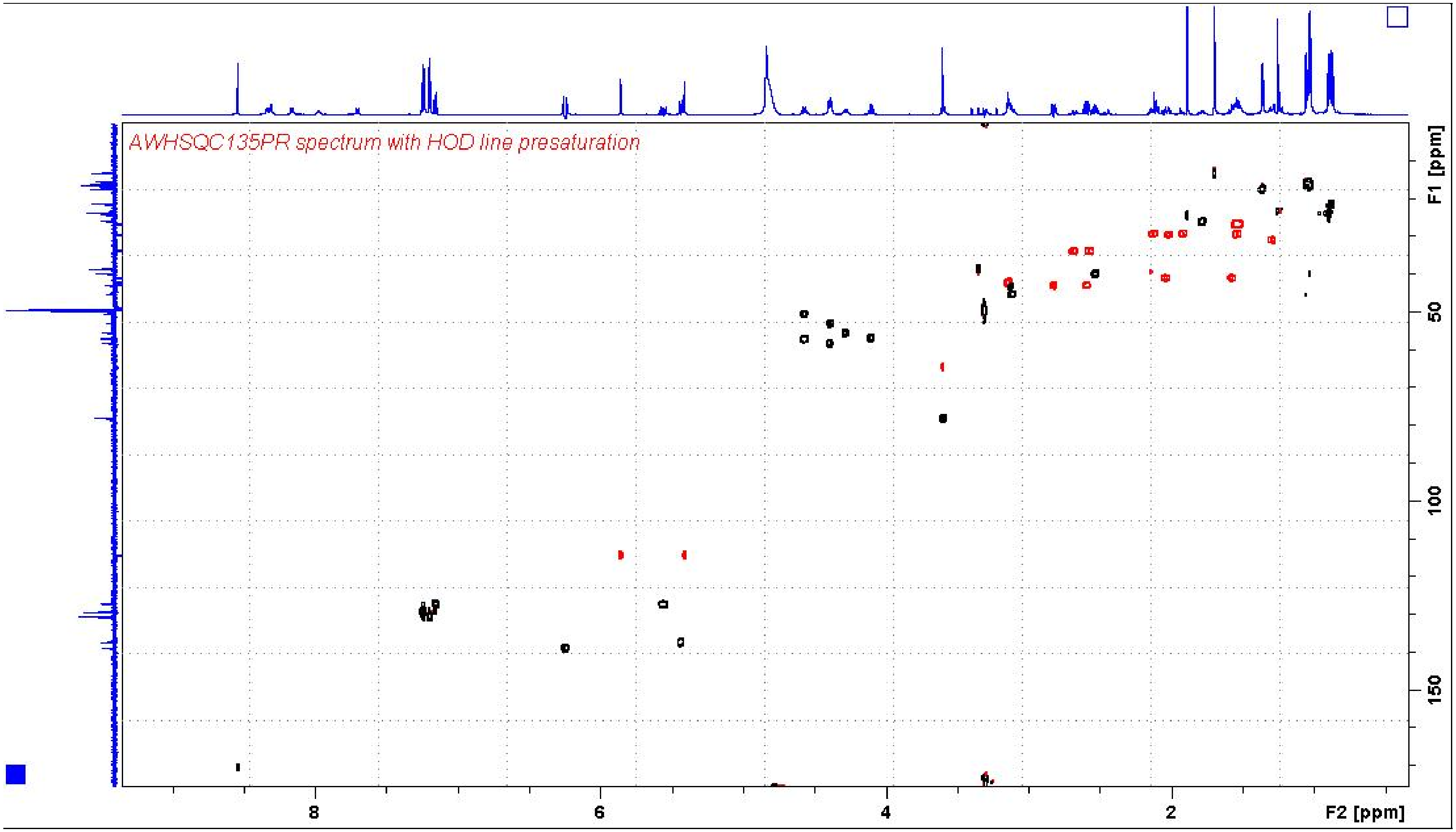

Figure S19: Edited HSQC135 NMR spectrum of [DMAdda[5]]MC-LR (**1**) in CD_3OH.

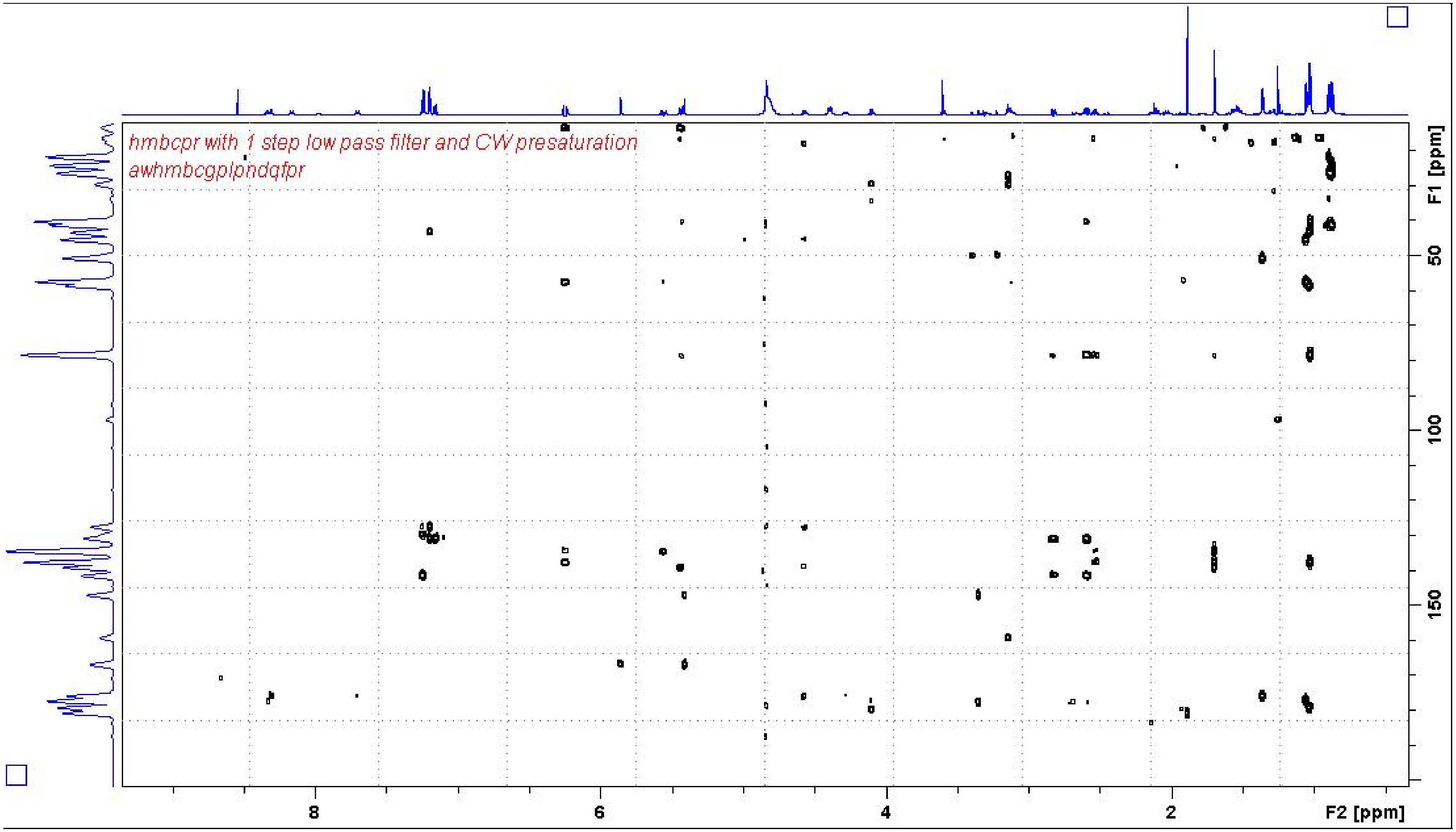

Figure S20: HMBC NMR spectrum of [DMAdda[5]]MC-LR (**1**) in CD_3OH.

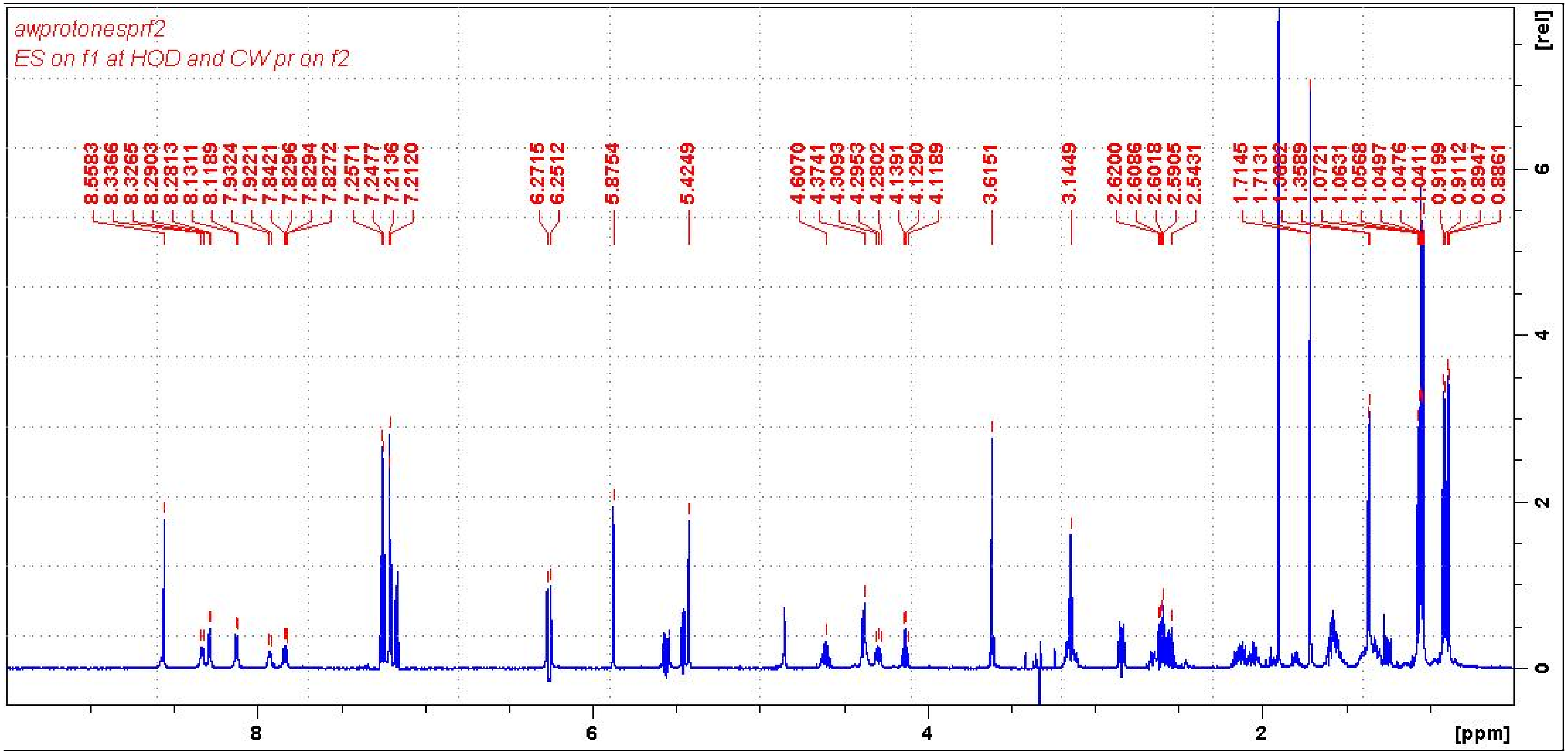

Figure S21: ^{1}H NMR spectrum of [DMAdda^{5}]MC-LHar (**2**) in CD_3OH.

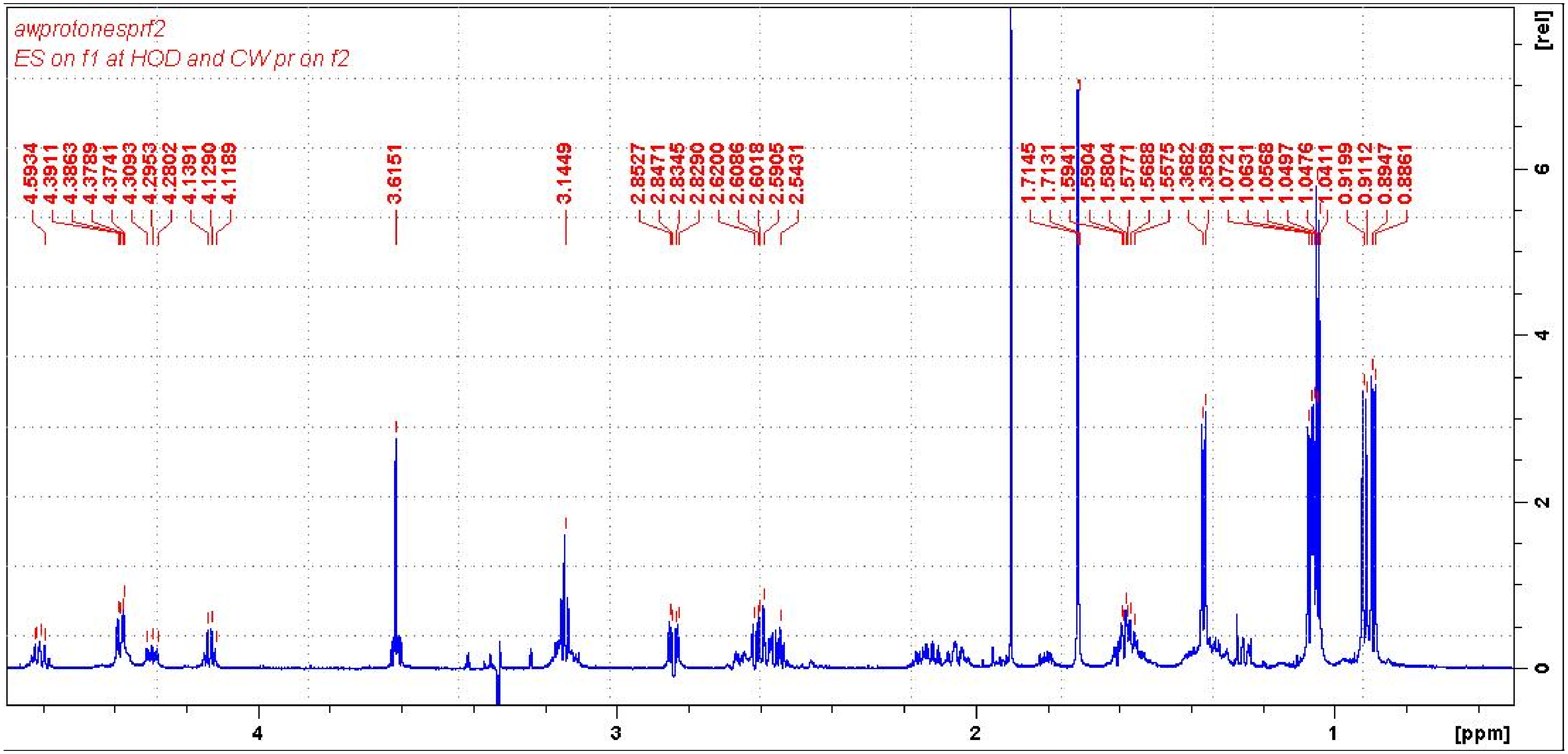

Figure S22: Expansion (0.5–4.7 ppm) of the ^{1}H NMR spectrum of [DMAdda^5]MC-LHar (**2**) in CD_3OH.

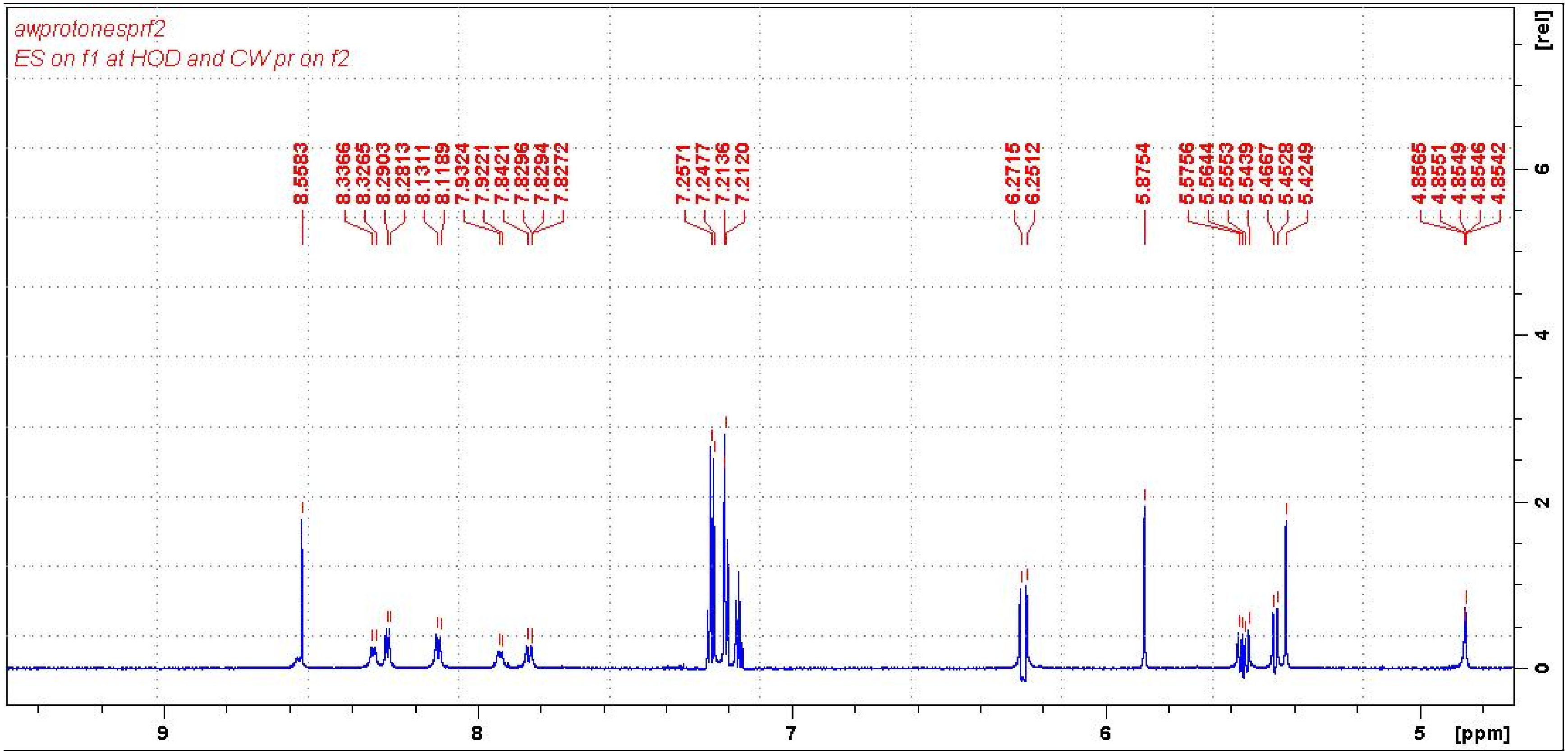

Figure S23: Expansion (4.7–9.5 ppm) of the ^{1}H NMR spectrum of [DMAdda^{5}]MC-LHar (**2**) in CD_3OH.

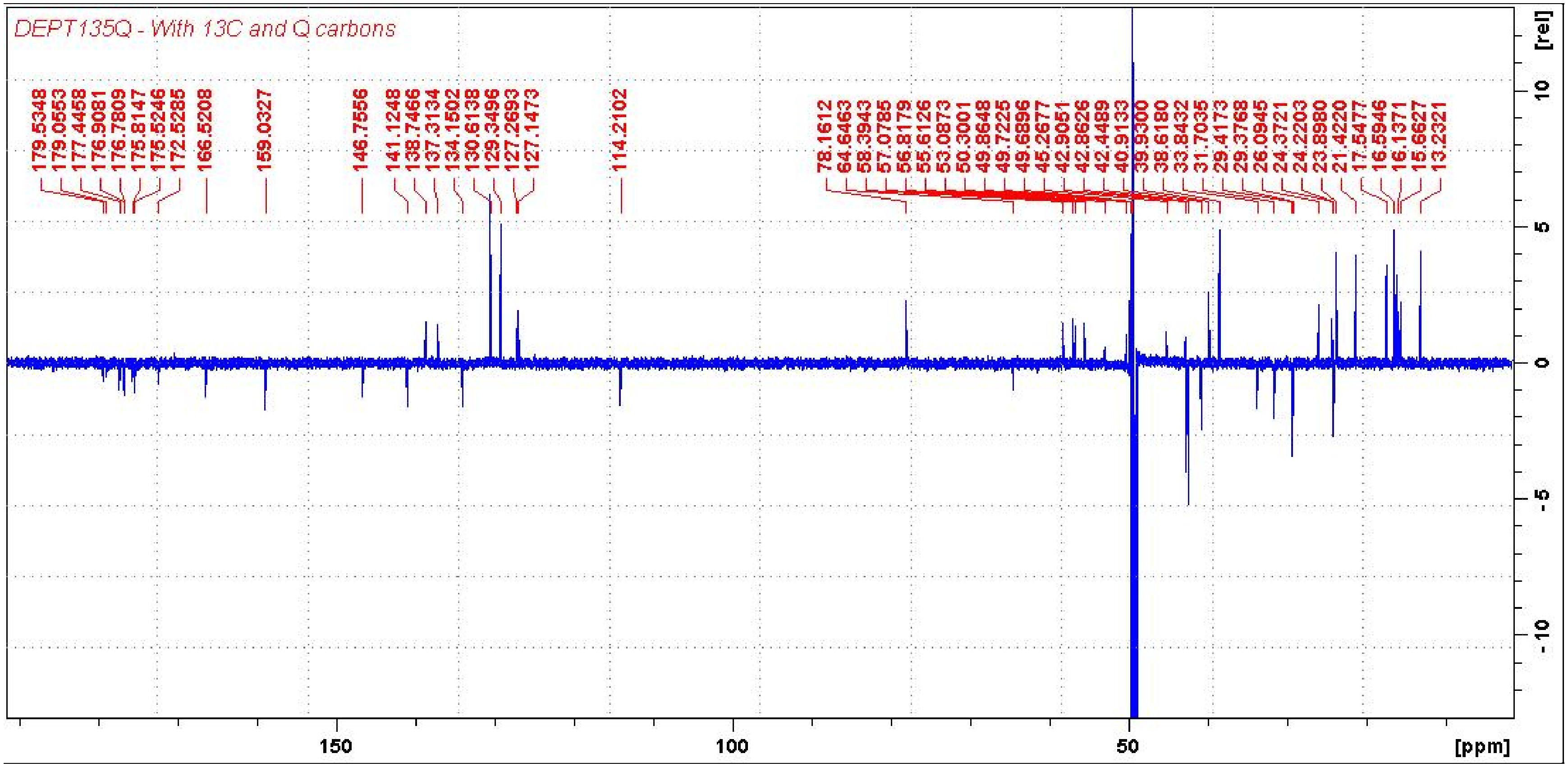

Figure S24: DEPT135Q NMR spectrum of [DMAdda[5]]MC-LHar (**2**) in CD_3OH.

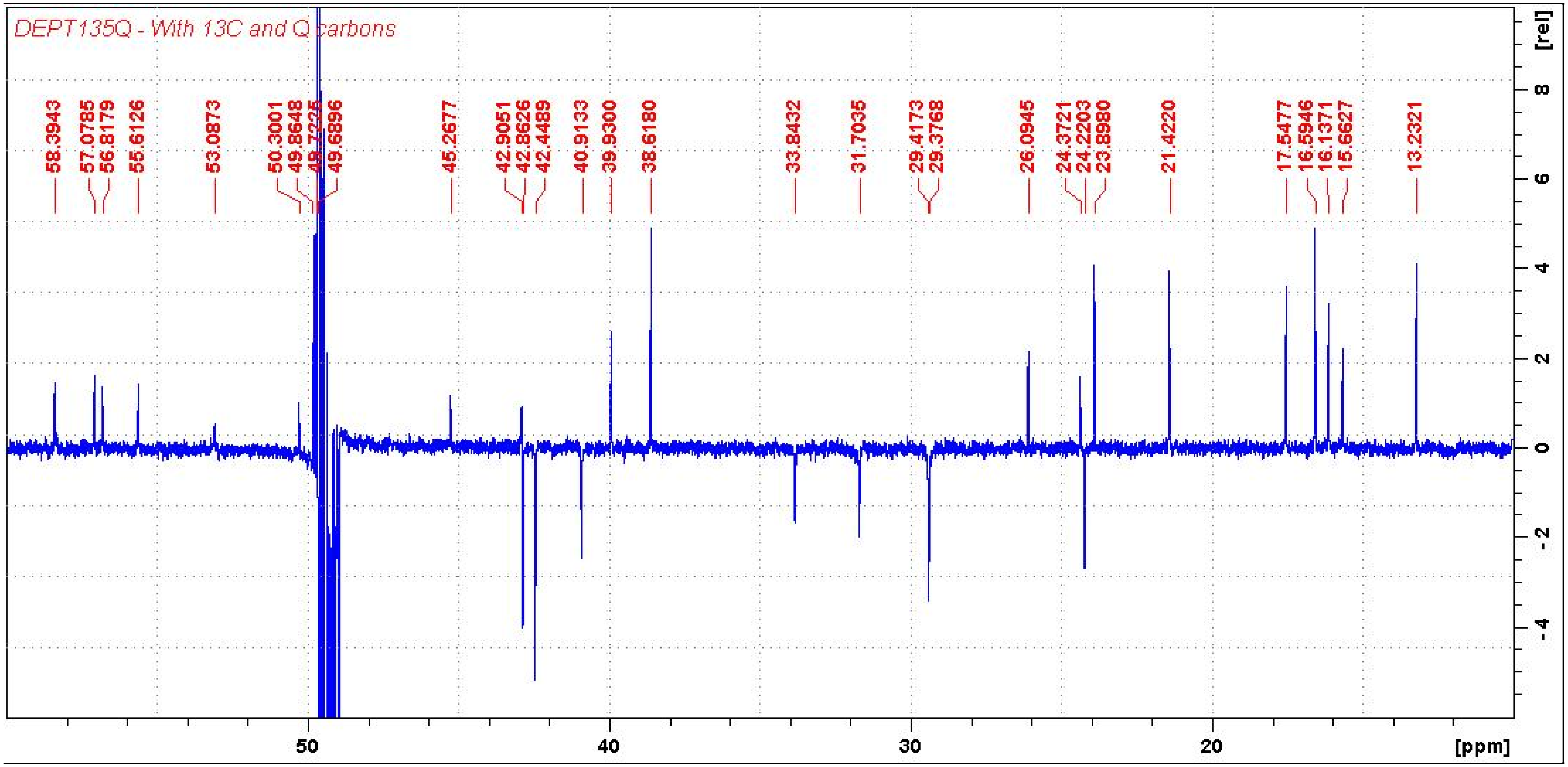

Figure S25: Expansion (10–60 ppm) of the DEPT135Q NMR spectrum of [DMAdda^{5}]MC-LHar (**2**) in CD_3OH.

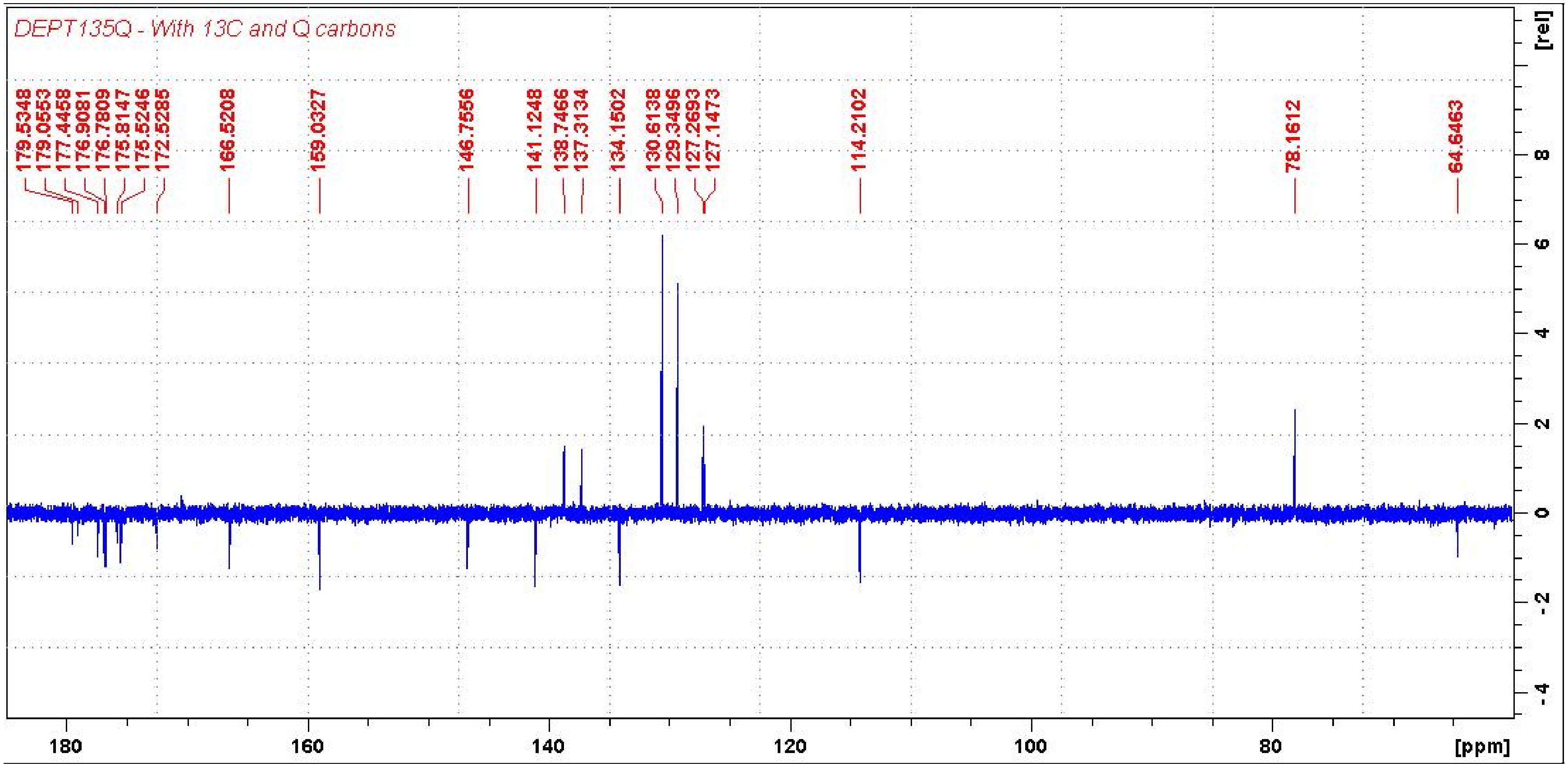

Figure S26: Expansion (70–190 ppm) of the DEPT135Q NMR spectrum of [DMAdda[5]]MC-LHar (**2**) in CD_3OH.

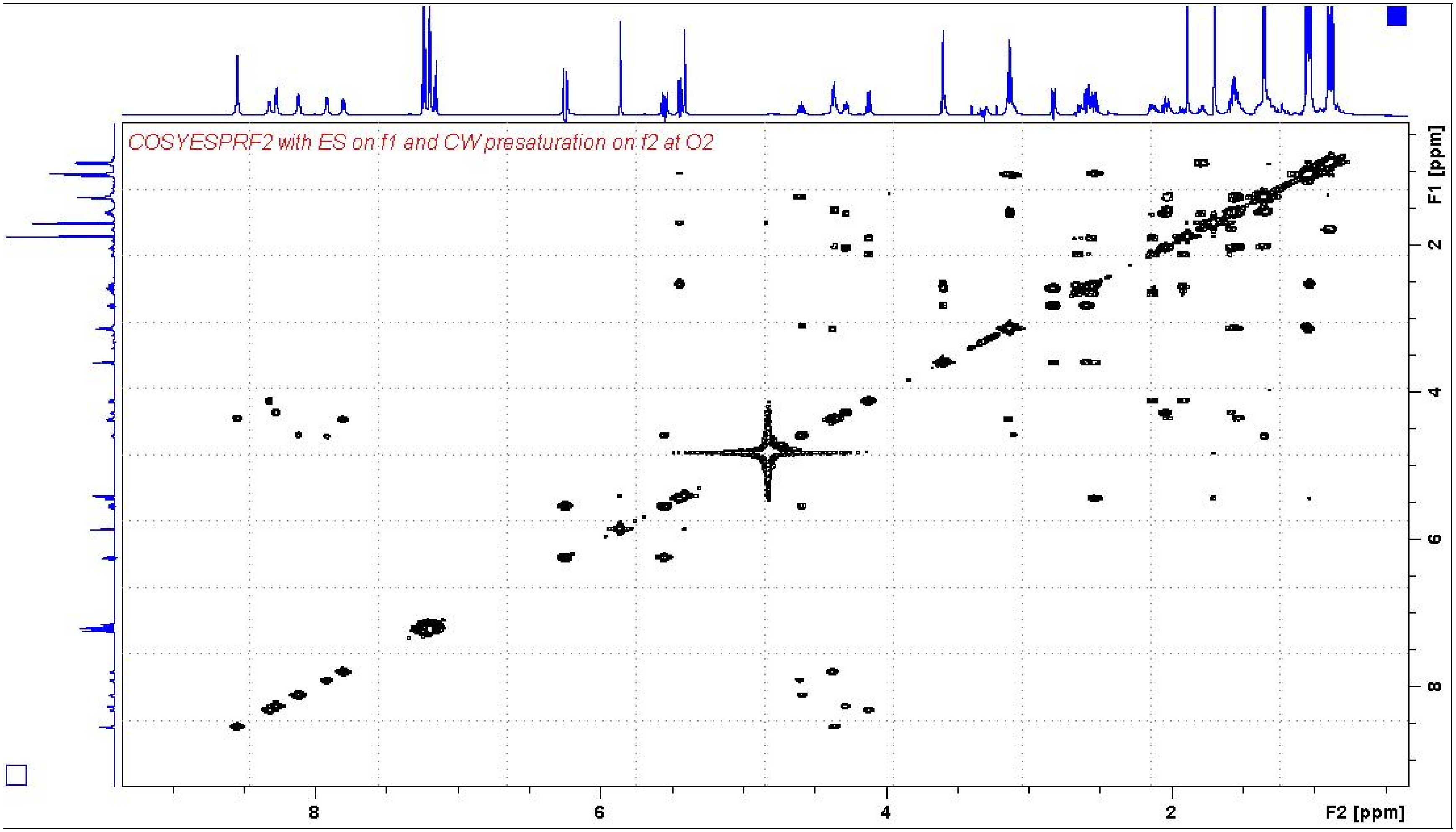

Figure S27: COSY NMR spectrum of [DMAdda[5]]MC-LHar (**2**) in CD_3OH.

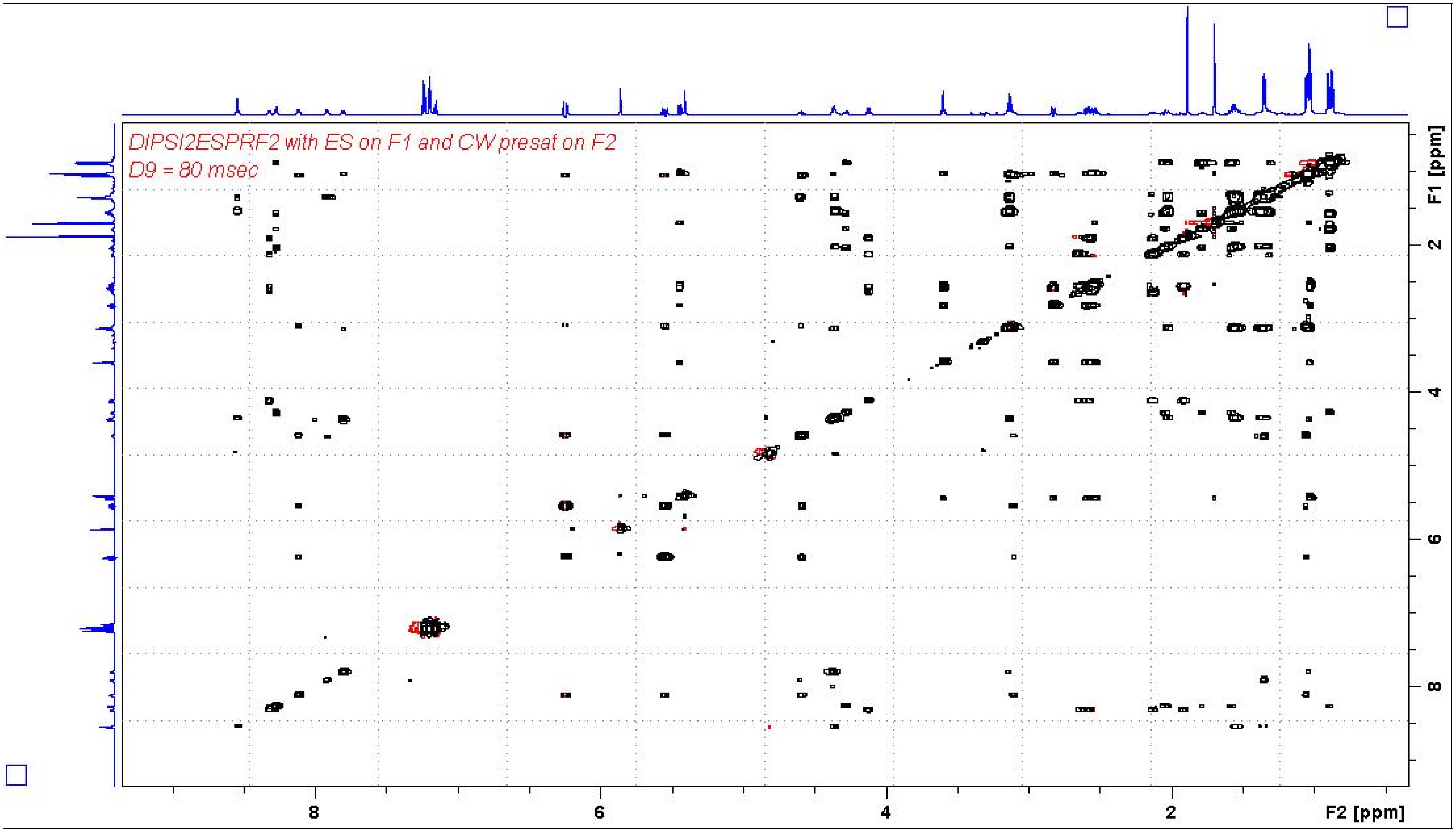

Figure S28: DIPSI2 (80 ms) NMR spectrum of [DMAdda⁵]MC-LHar (**2**) in CD_3OH.

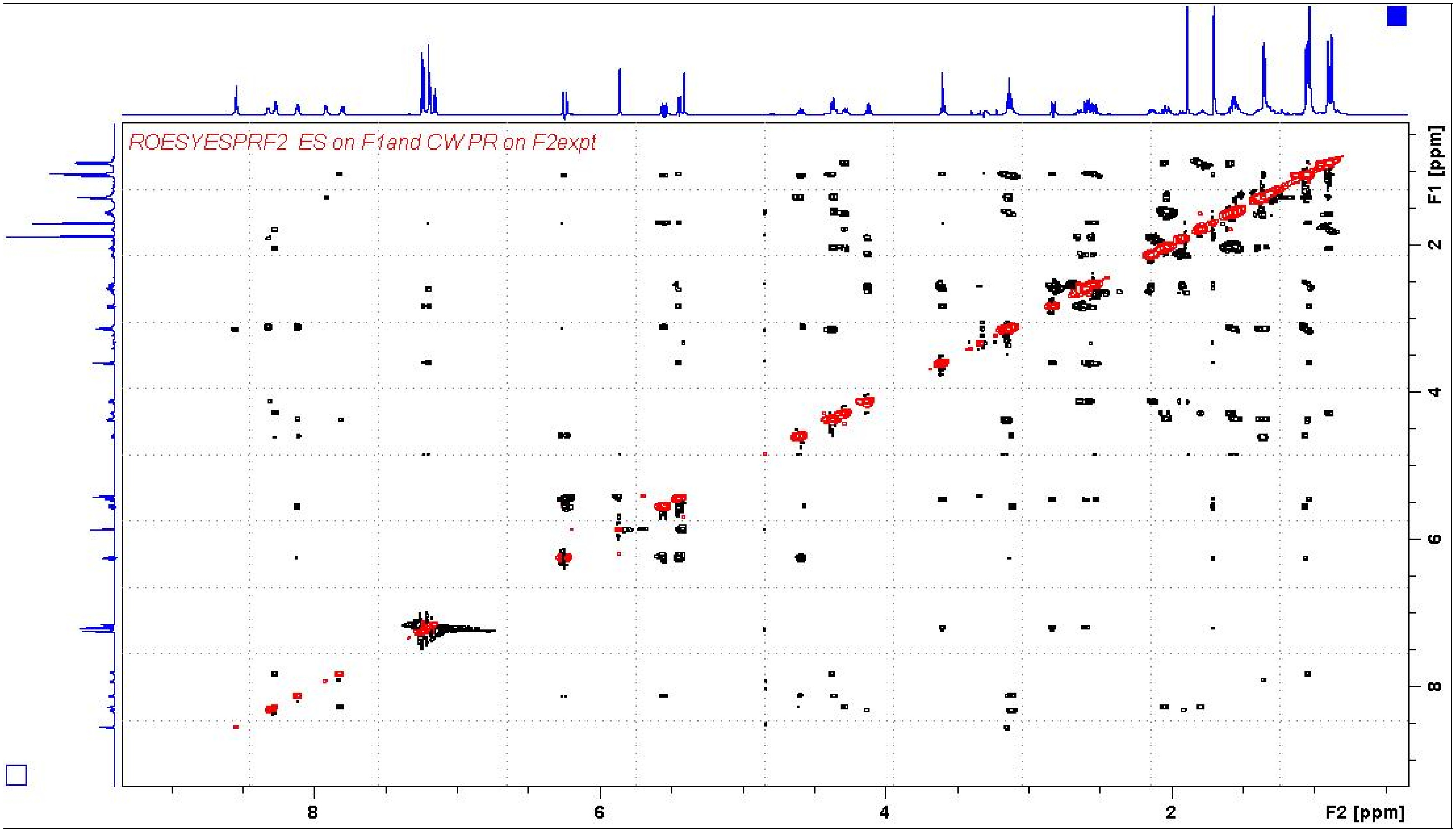

Figure S29: ROESY NMR spectrum of [DMAdda[5]]MC-LHar (**2**) in CD_3OH.

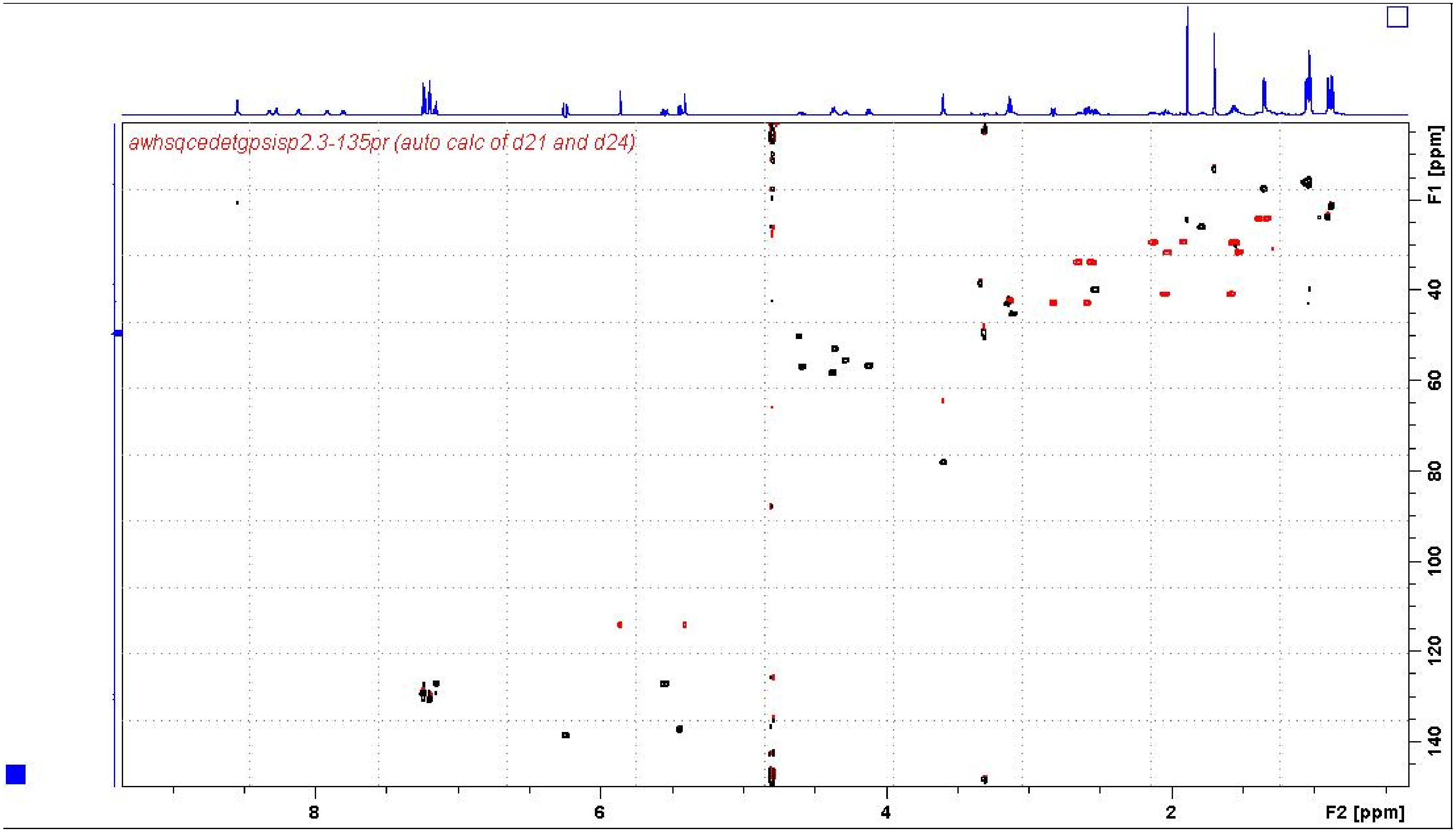

Figure S30: Edited HSQC135 NMR spectrum of [DMAdda[5]]MC-LHar (**2**) in CD_3OH.

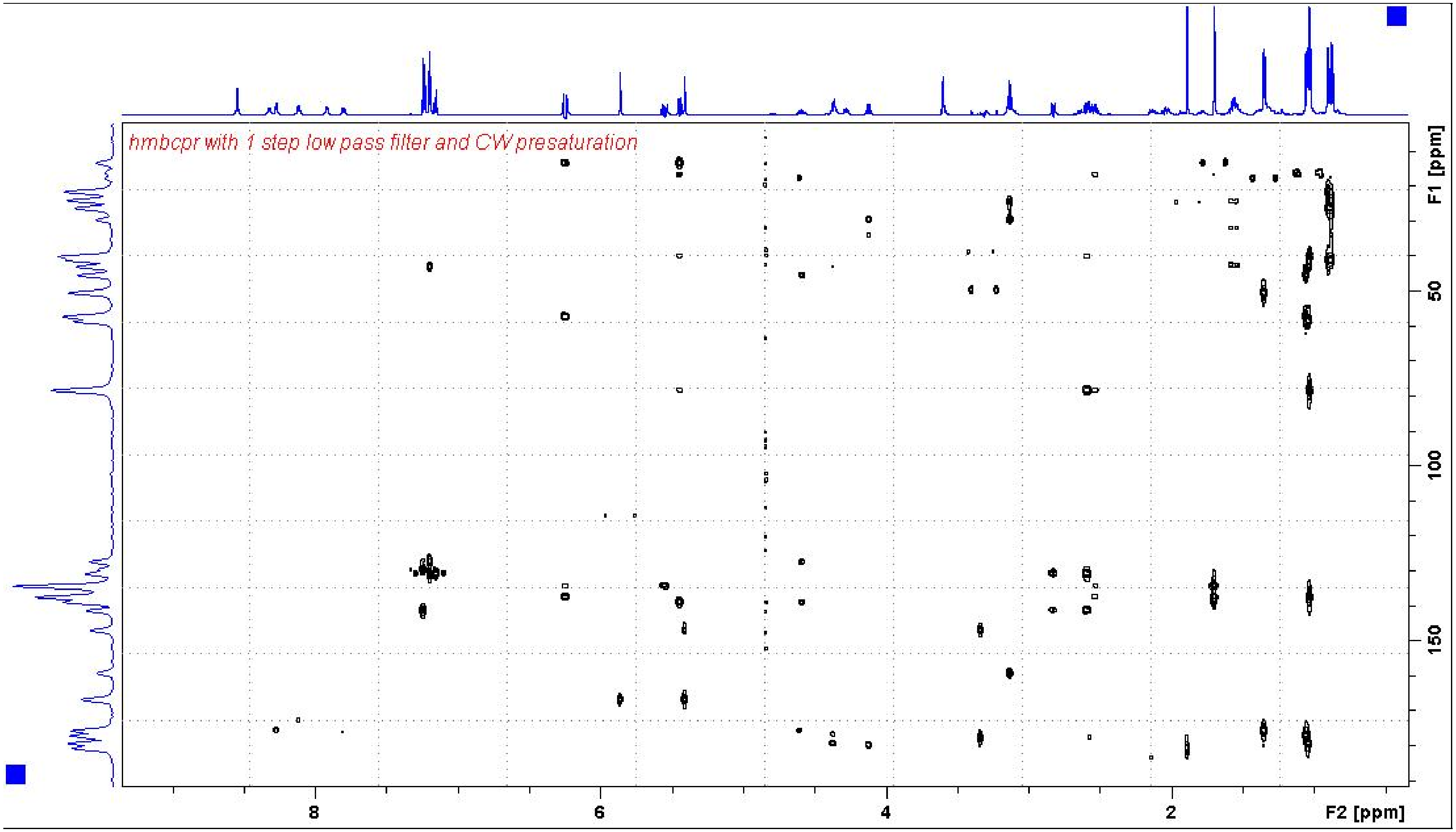

Figure S31: HMBC NMR spectrum of [DMAdda[5]]MC-LHar (**2**) in CD_3OH.

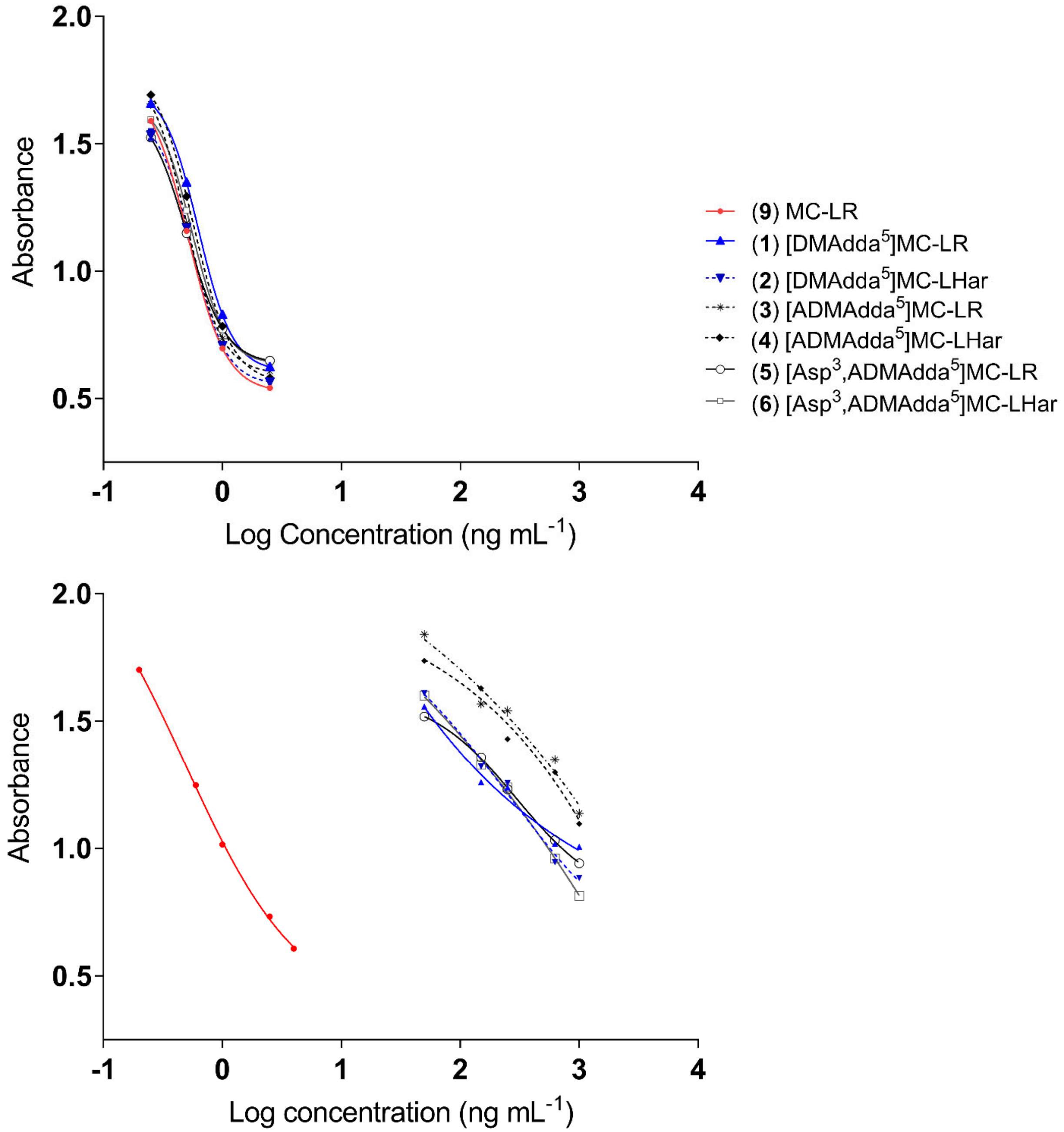

Figure S32: PP2A Inhibition assay (top) and Adda-ELISA (bottom) curves generated from MC-LR (**9**) in water as compared to the isolated ADMAdda/DMAdda MCs (**1–6**) presented on the same x-axis scale (0.1 to 1000 ng mL^{-1}). Four parameter logistic regression was used to fit each curve.

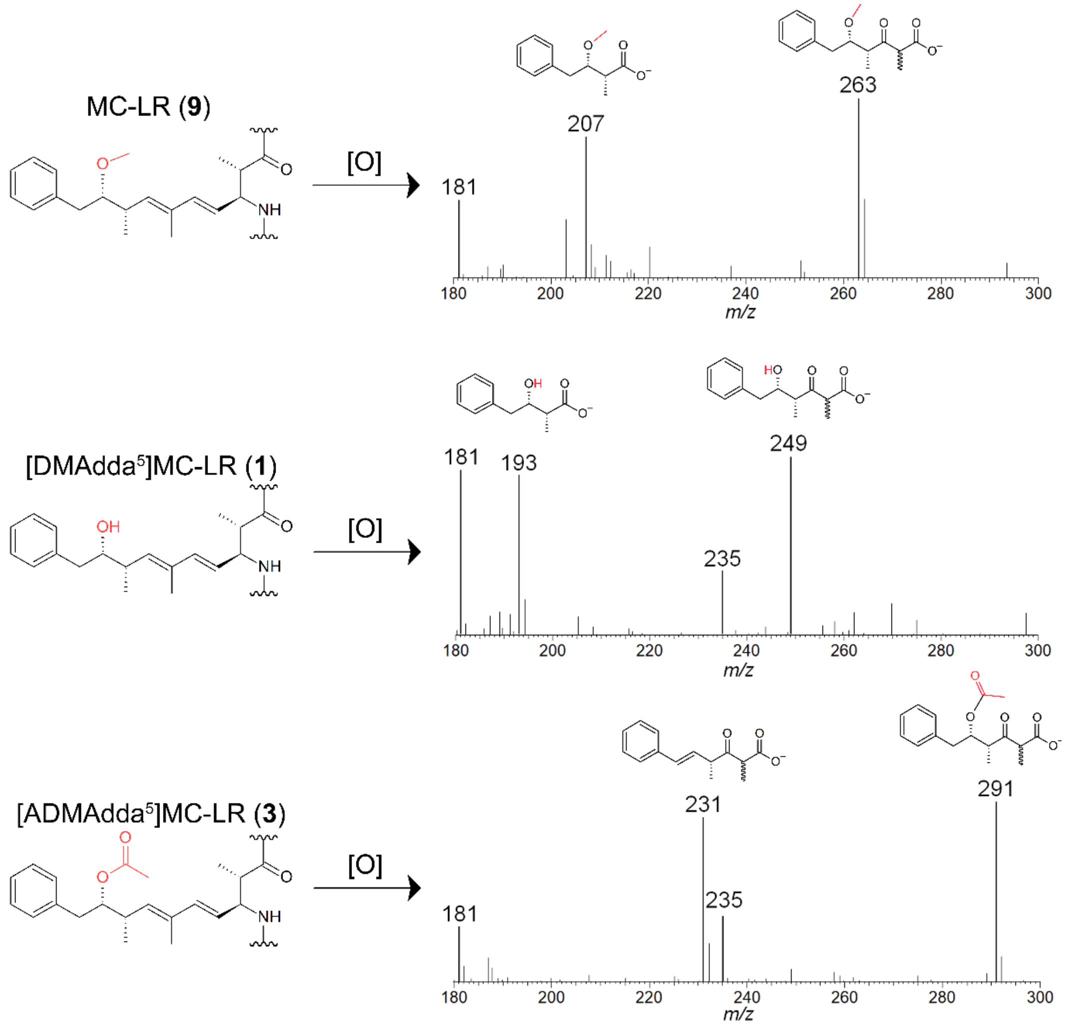

Figure S33: Ion trap full-scan mass spectra in negative ionization mode from LC–MS analysis of oxidized standards of MC-LR (**9**), [DMAdda^5]MC-LR (**1**) and [ADMAdda^5]MC-LR (**3**) used to identify target oxidation products, including MMPB (*m/z* 207), MHPB (*m/z* 193) and MOMAPH (*m/z* 291). The spectra were obtained from 2 to 10 min retention times. The ion corresponding to MAPB (*m/z* 235) was observed in both [ADMAdda^5]MC-LR and [DMAdda^5]MC-LR oxidations. The *m/z* 231 in the lower spectrum likely represents the facile loss of the acetyl group from *m/z* 291 in the source. The *m/z* 181 was present in all oxidations but not in blanks.

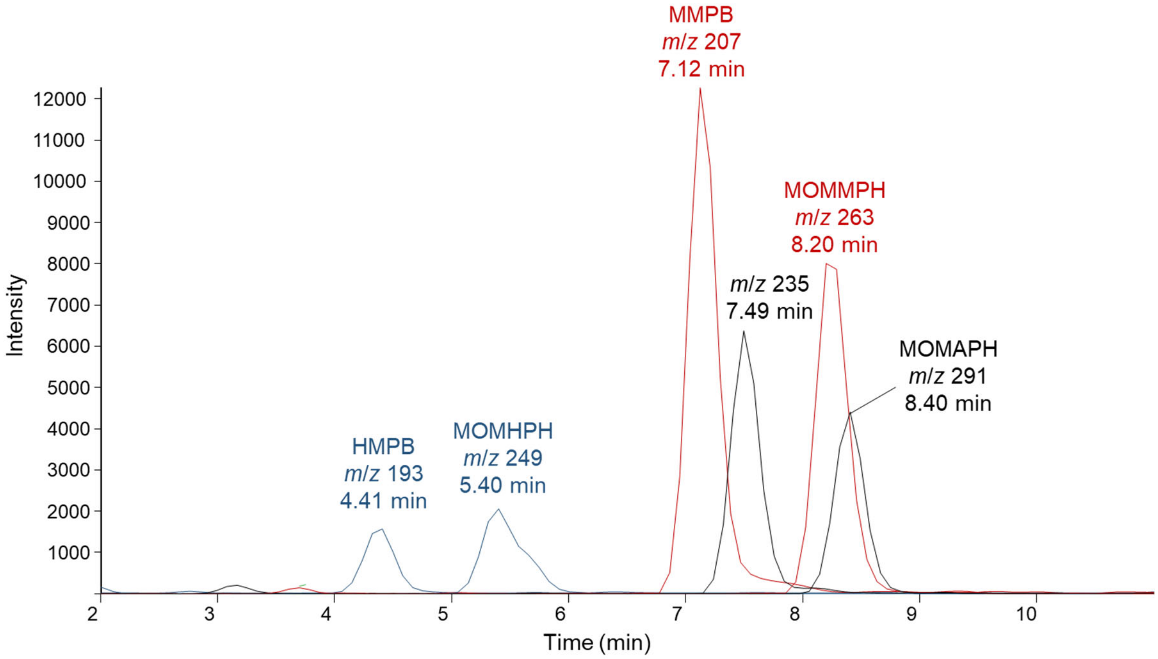

Figure S34: Triple quadrupole LC–MS/MS chromatogram of all observed oxidation products, including those arising from DMAdda- (HMPB, MOMHPH, *m*/*z* 235), Adda- (MMPB, MOMMPH) and ADMAdda-containing (*m*/*z* 235, MOMAPH) MCs.

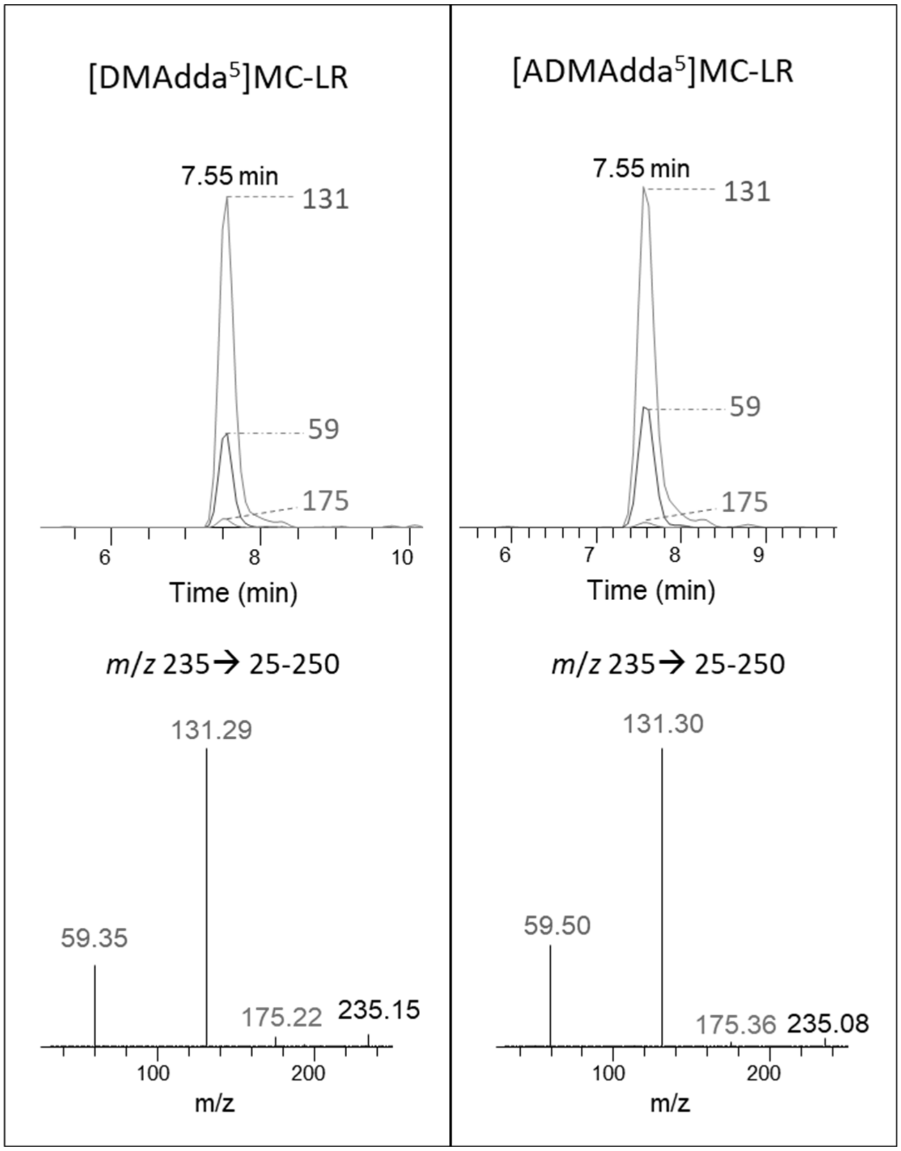

Figure S35: Triple quadrupole LC–MS/MS chromatograms and spectra from the peak with *m*/*z* 235 arising from oxidation of [DMAdda^5]MC-LR (**1**) (left) and [ADMAdda^5]MC-LR (**3**) (right). Both **1** and **3** provided peaks (top) in negative ionization mode with identical retention times (7.55 min) and spectra (bottom). Therefore, it was determined that the target product of ADMAdda oxidation (MAPB; *m*/*z* 235) did not present a unique ion and it was not utilized for ADMAdda analysis.

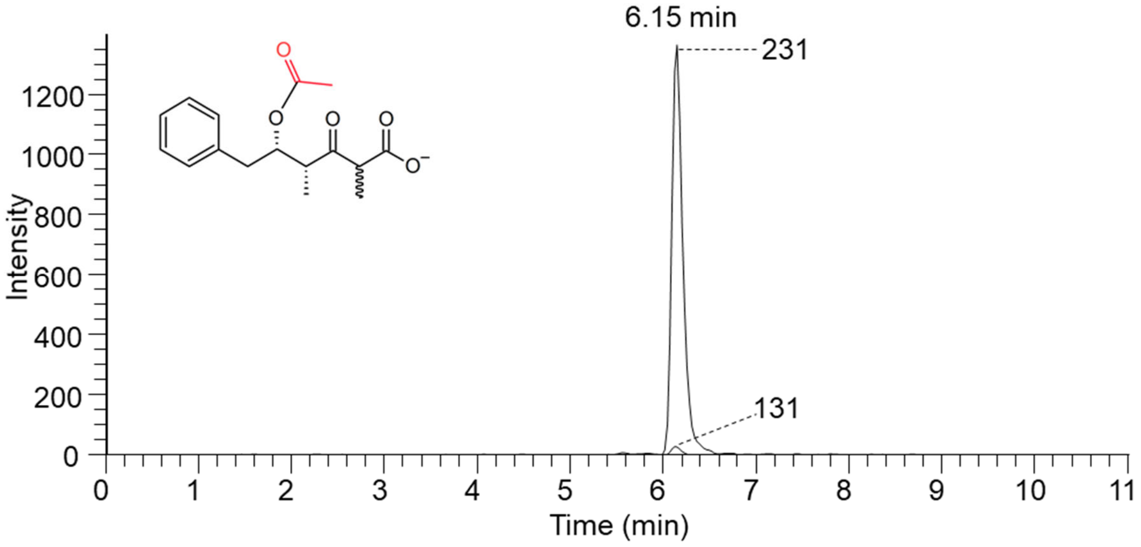

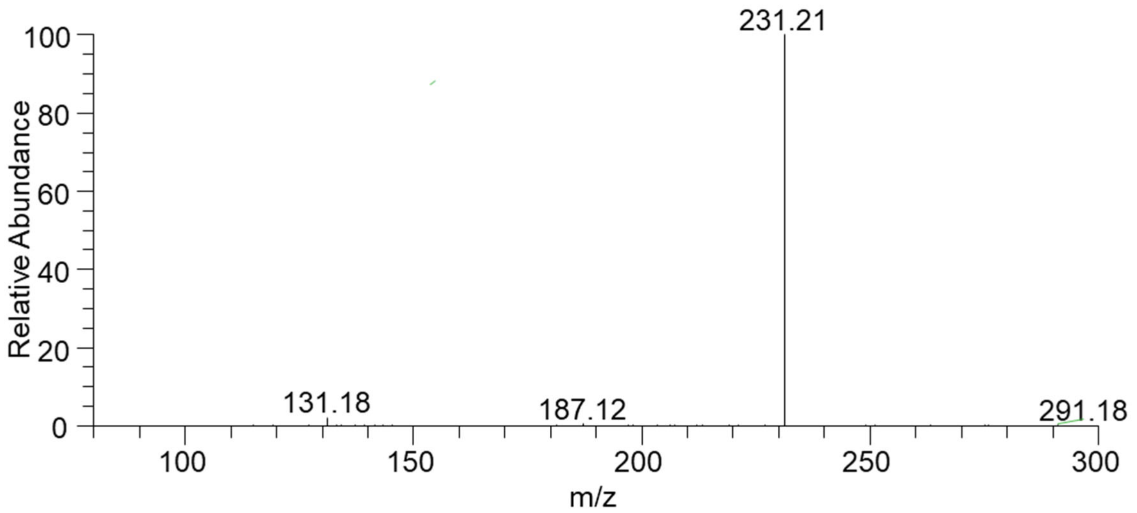

Figure S36: Ion trap LC–MS/MS chromatogram and spectrum of MOMAPH (*m*/*z* 291; negative ionization) from oxidized [ADMAdda^5]MC-LR (**3**).

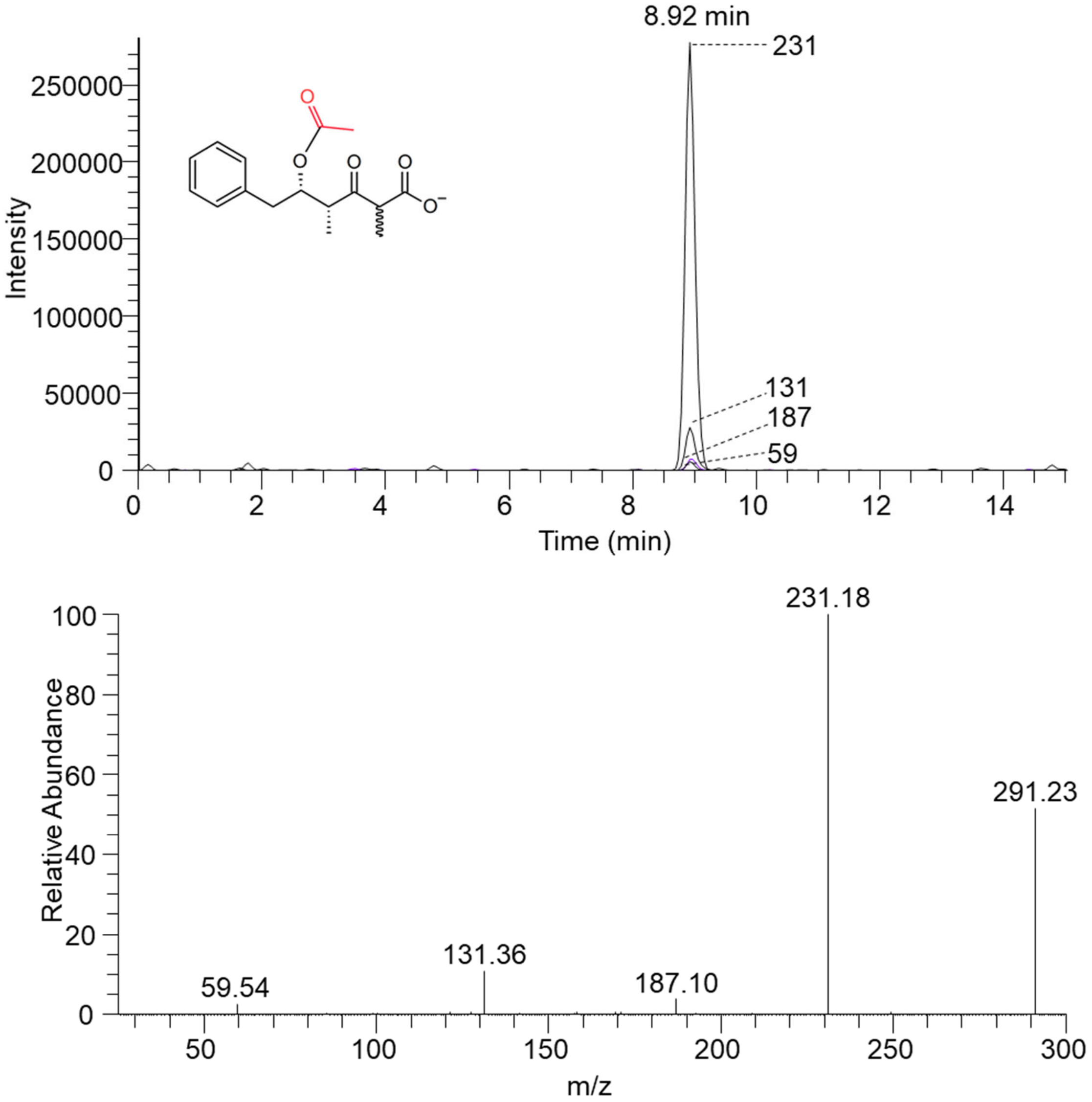

Figure S37: Triple quadrupole LC–MS/MS chromatogram and spectrum of MOMAPH (*m*/*z* 291; negative ionization) from oxidized [ADMAdda^5]MC-LR (**3**).

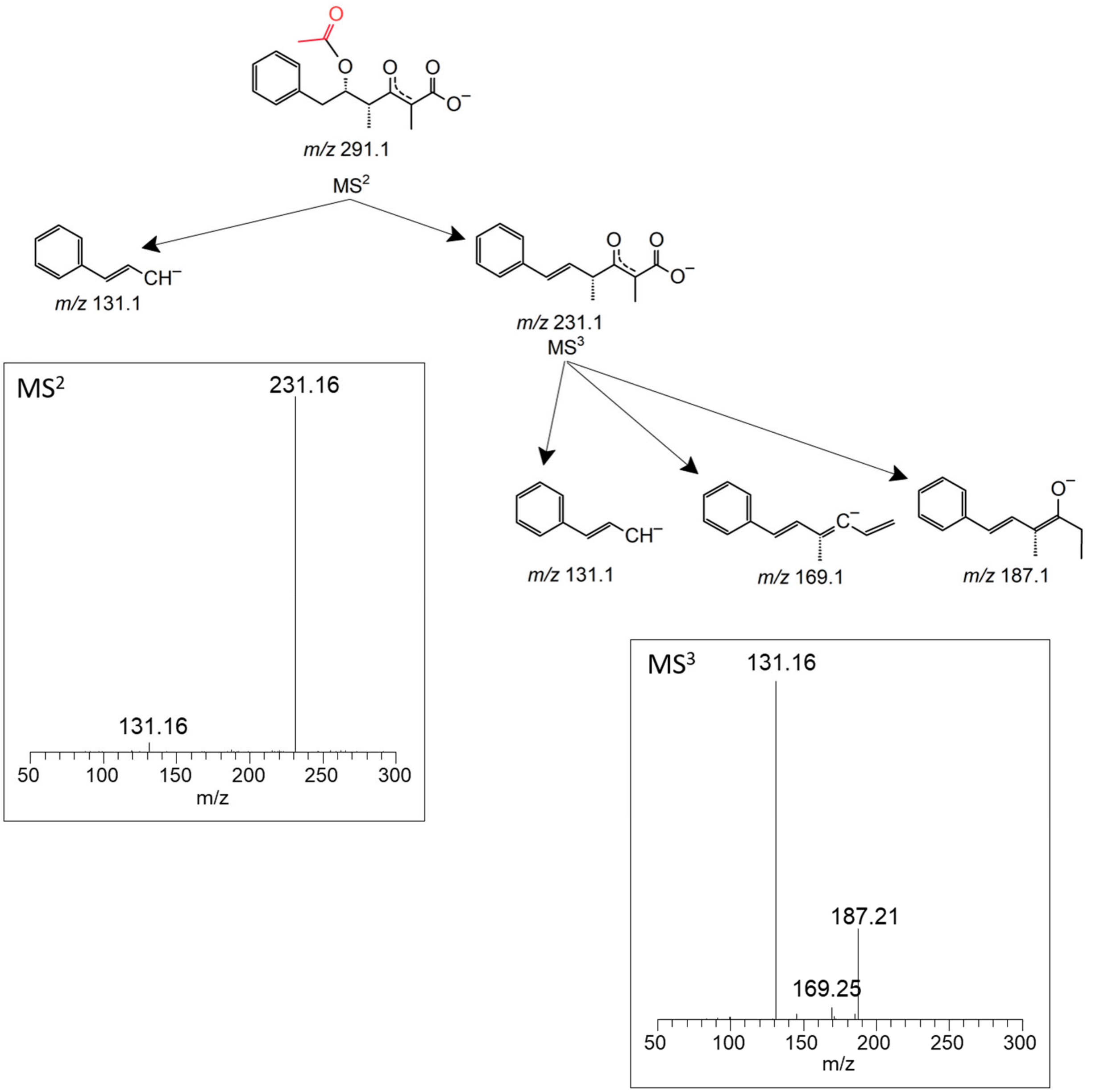

Figure S38: Ion trap negative ionization LC–MS/MS and –MS/MS/MS spectra of MOMAPH from oxidized [ADMAdda^{5}]MC-LR (**3**), with proposed fragmentation. The left-hand spectrum is *m*/*z* 291 fragmented CID 20% CE (-ve) in MS/MS scan mode with right-hand spectrum showing the MS/MS/MS of *m*/*z* 231.

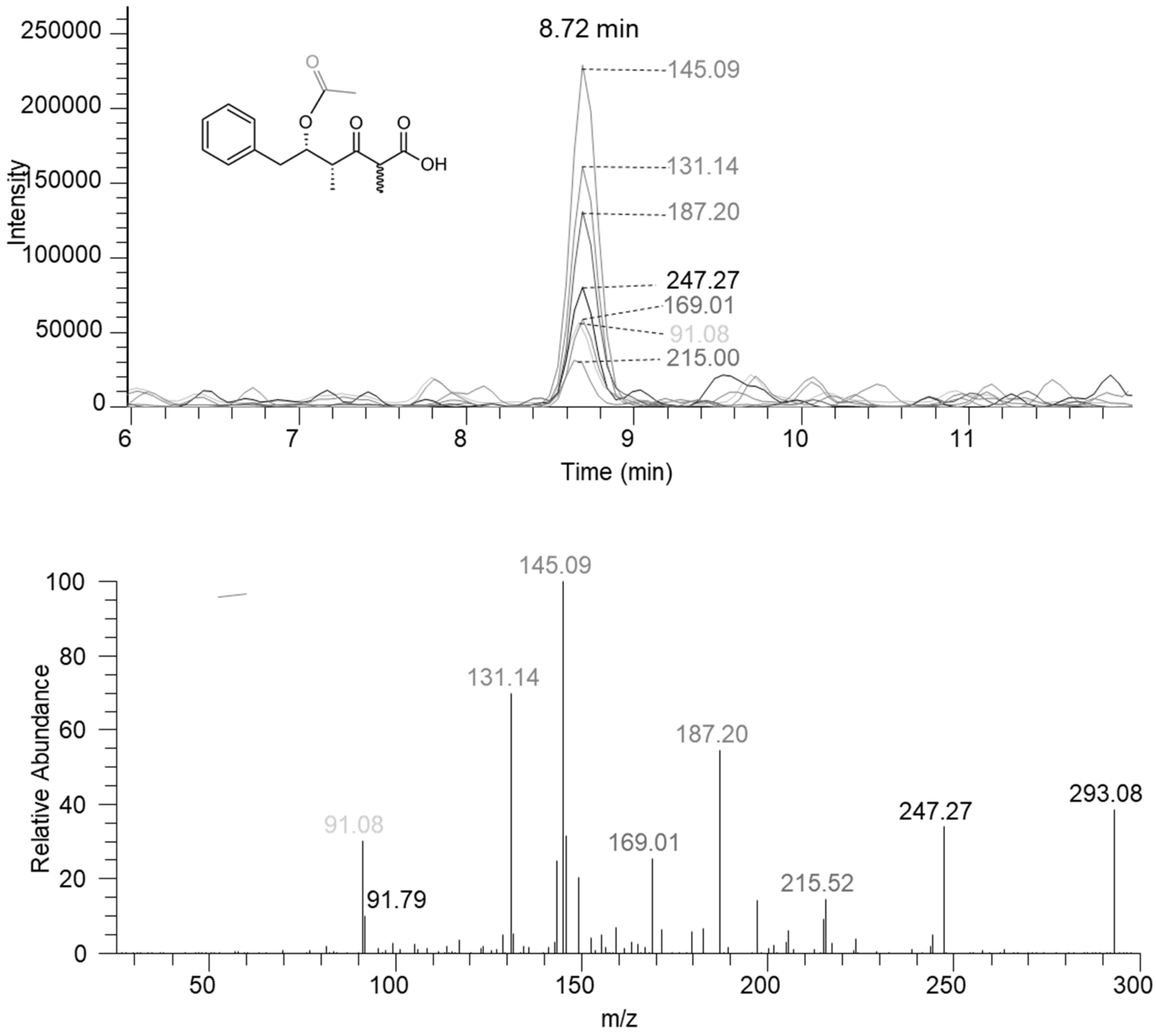

Figure S39: Ion trap LC–MS/MS chromatogram and spectrum of MOMAPH (*m*/*z* 293; positive ionization) from oxidized [ADMAdda^{5}]MC-LR (**3**).

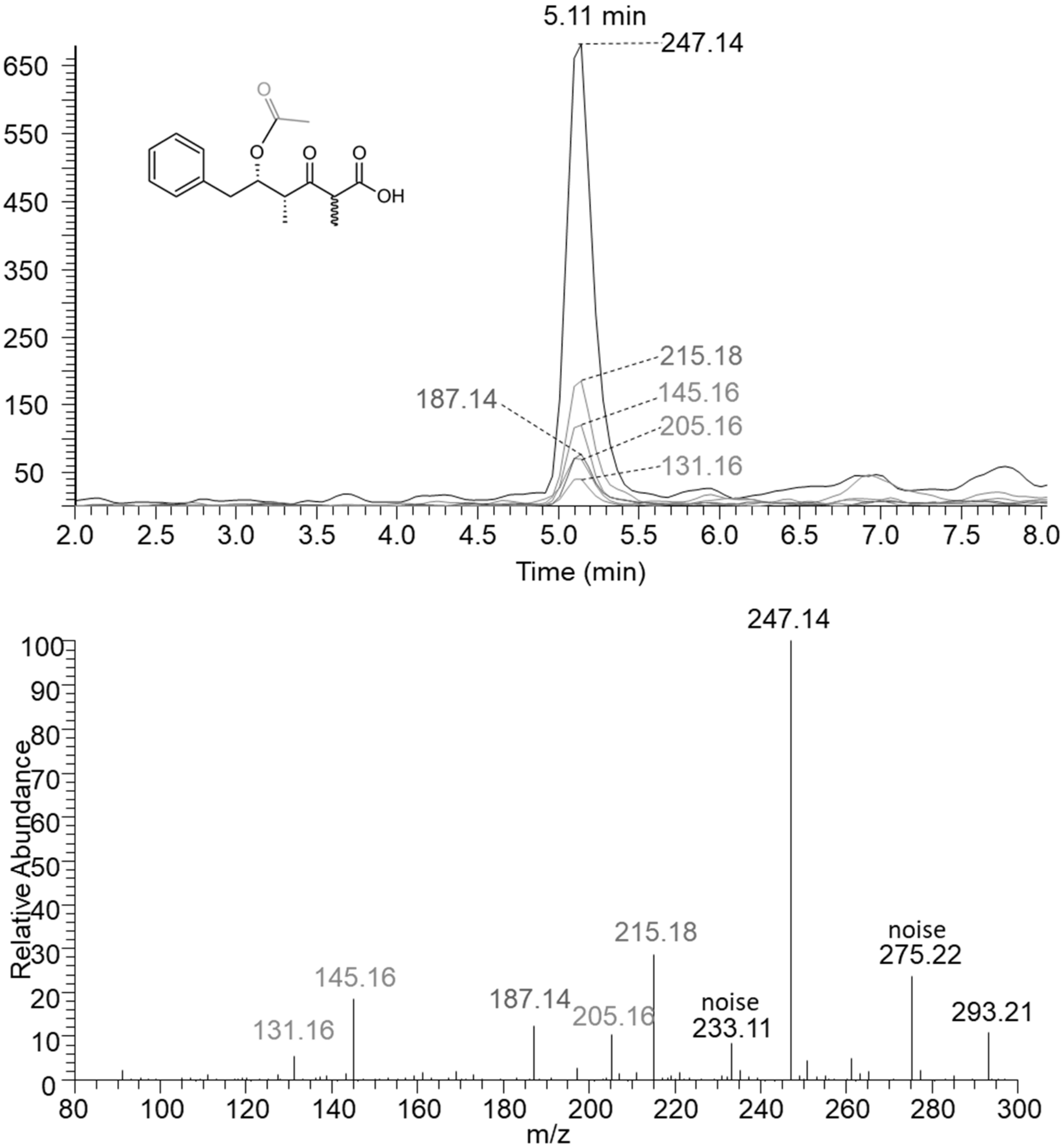

Figure S40: Triple-quadrupole LC–MS/MS chromatogram and spectrum of MOMAPH (*m*/*z* 293; positive ionization) from oxidized [ADMAdda^{5}]MC-LR (**3**).

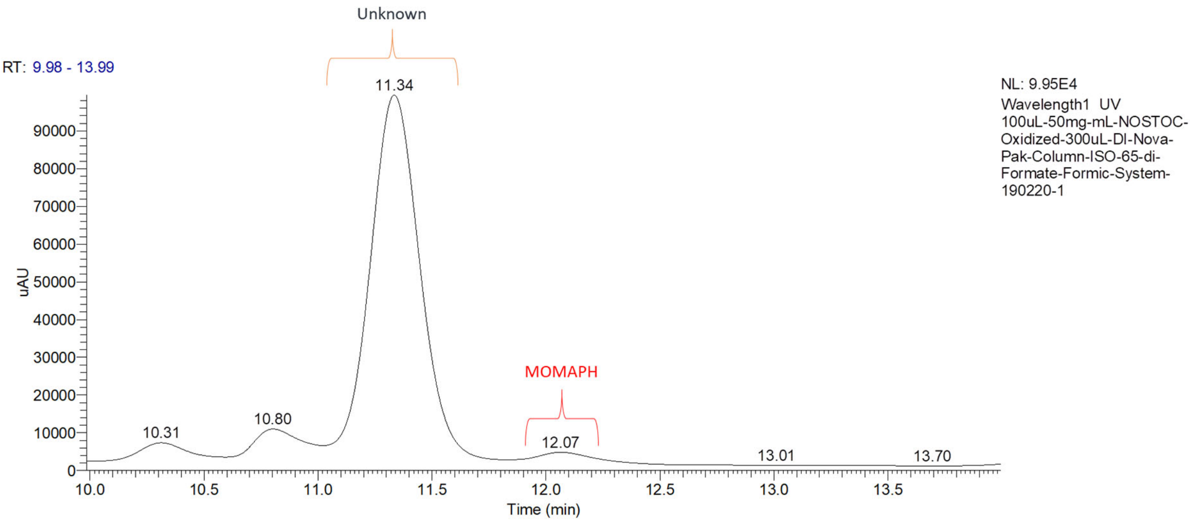

Figure S41: Semi-preparative HPLC–UV (254 nm) chromatogram of MOMAPH (from oxidized [ADMAdda^5]MC-LR (**3**)). The larger peak to the left represents an unknown with dominant signal at *m*/*z* 981 (+ve).

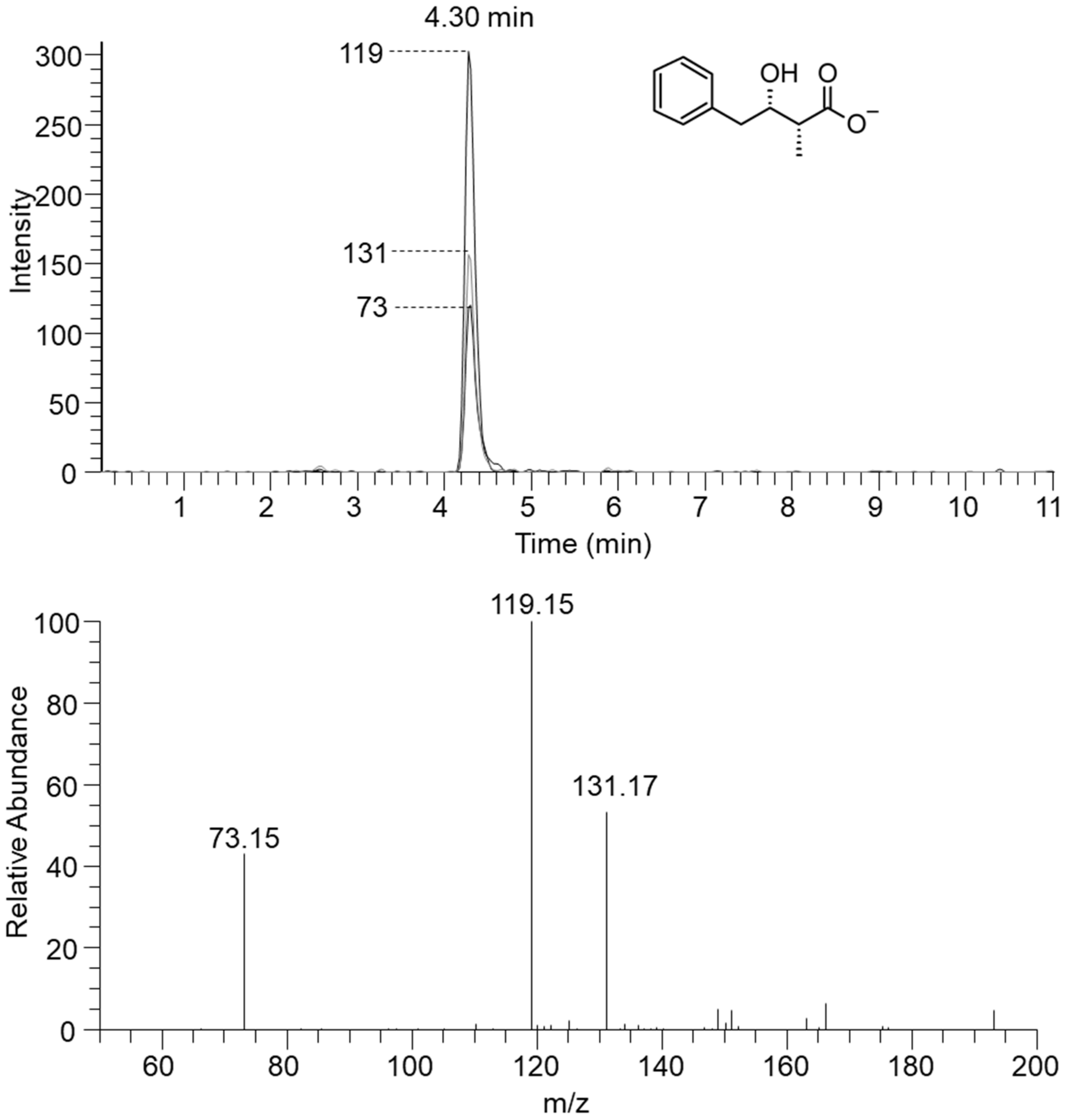

Figure S42: Ion trap LC–MS/MS chromatogram and spectrum of MHPB (*m*/*z* 193; negative ionization) from oxidized [DMAdda^{5}]MC-LR (**1**).

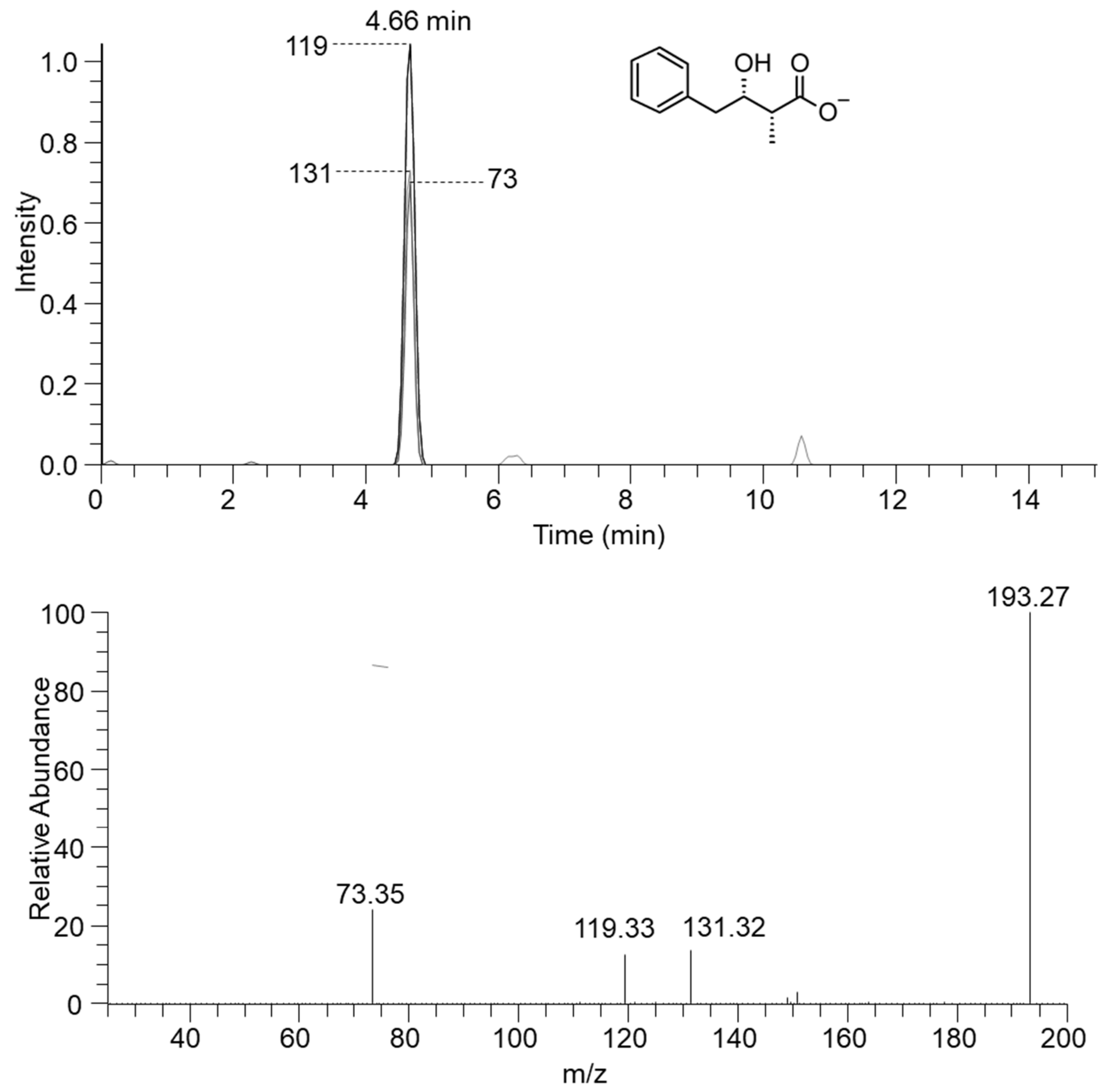

Figure S43: Triple-quadrupole LC–MS/MS chromatogram and spectrum of MHPB (*m*/*z* 193; negative ionization) from oxidized [DMAdda[5]]MC-LR (**1**).

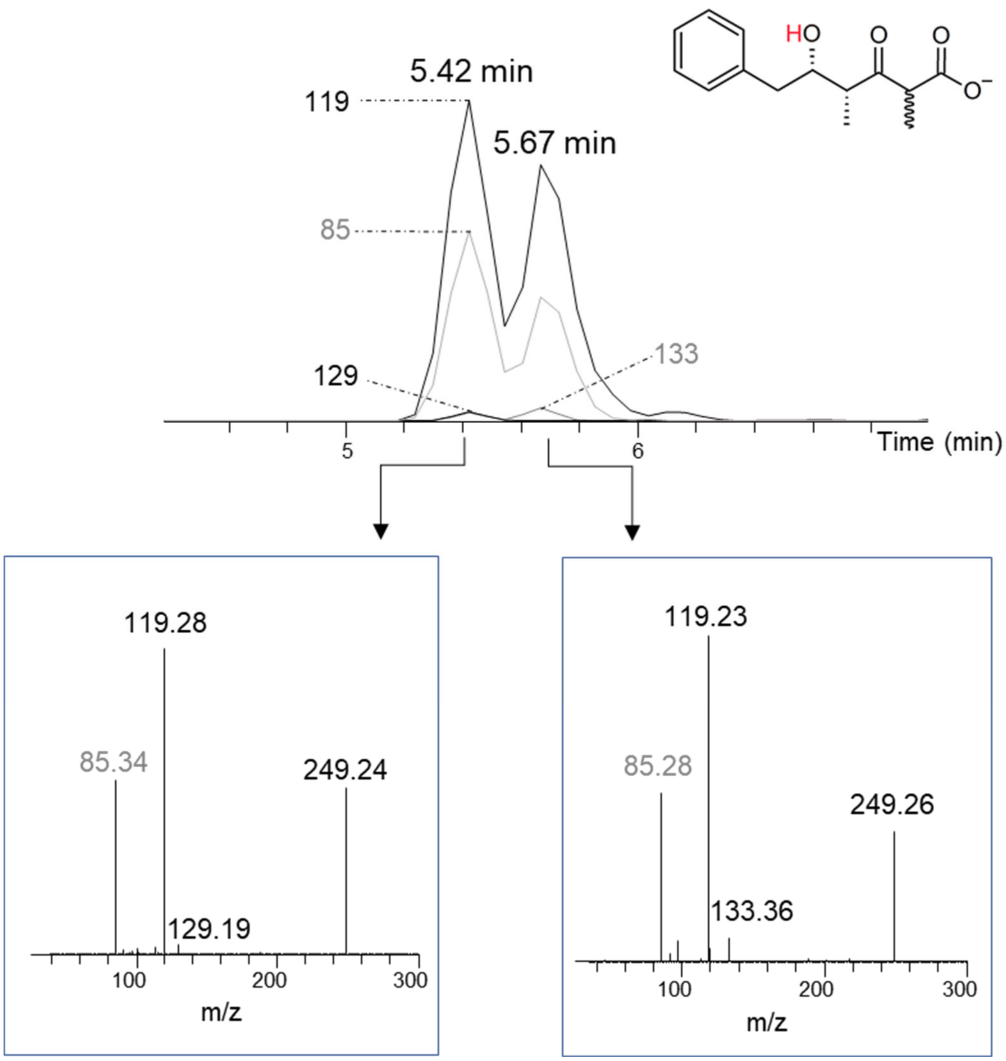

Figure S44: Triple-quadrupole LC–MS/MS chromatogram and spectra of MOMHPH (*m*/*z* 249; negative ionization) from oxidized [DMAdda^{5}]MC-LR (**1**). A double chromatographic peak was observed, possibly due to the production of isomers formed from the cleavage of 4,5-ene.

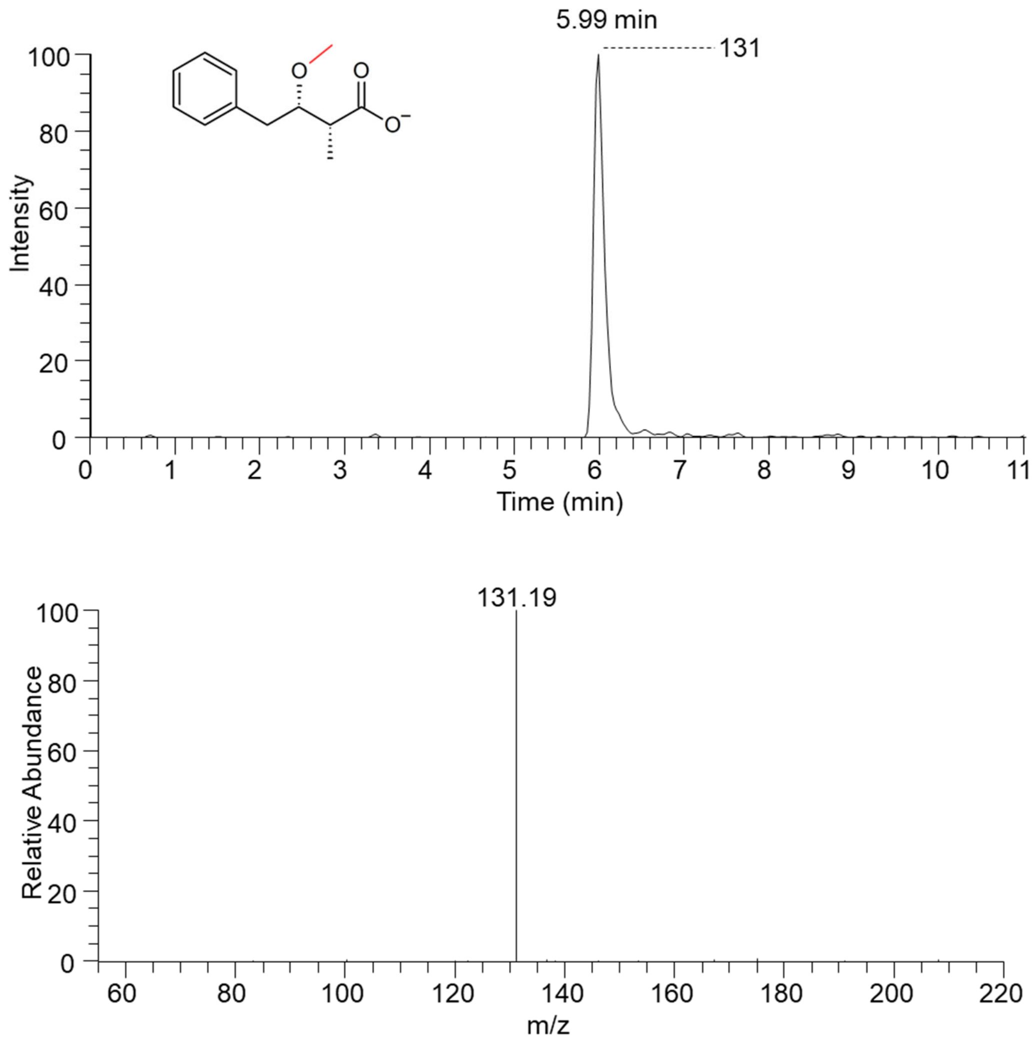

Figure S45: Ion trap LC–MS/MS chromatogram and spectrum of MMPB (*m*/*z* 207; negative ionization) from oxidized MC-LR (**9**).

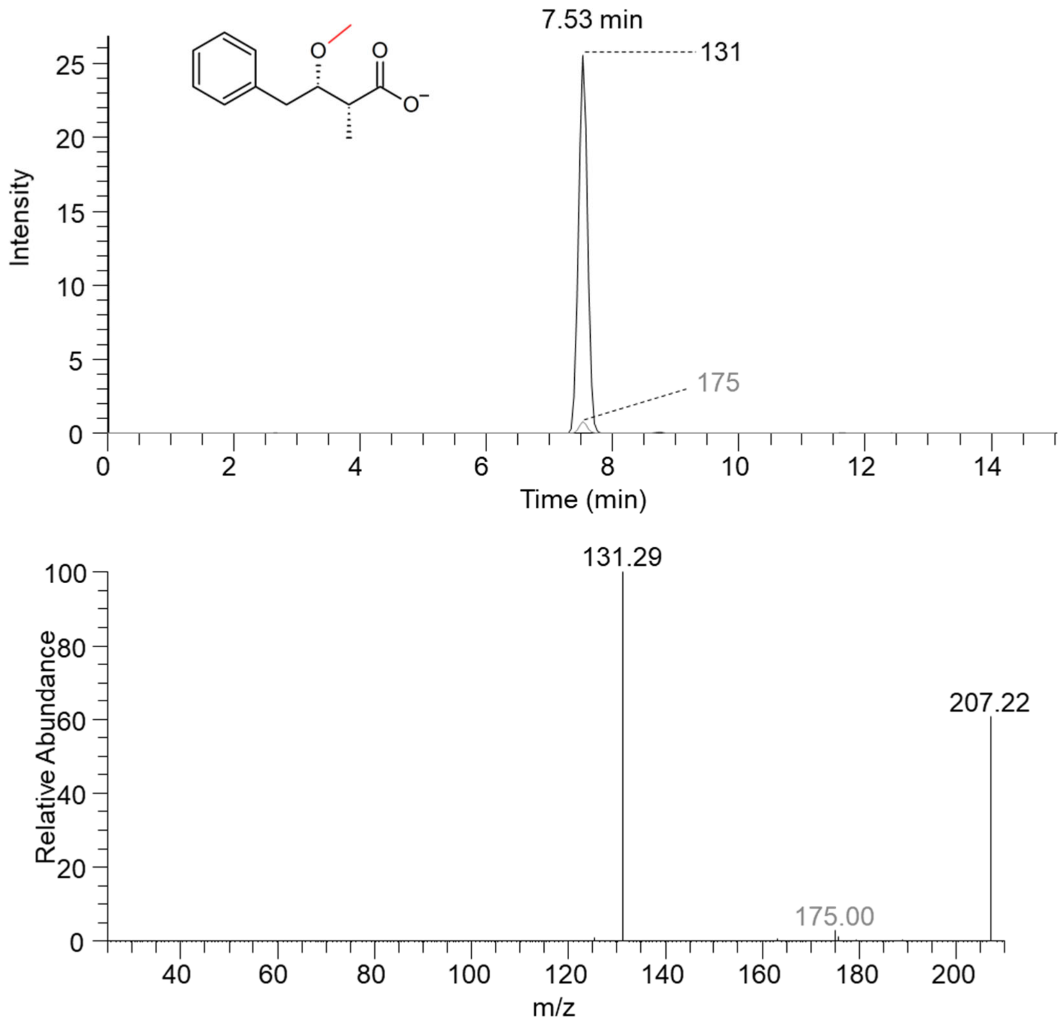

Figure S46: Triple-quadrupole LC–MS/MS chromatogram and spectrum of MMPB (*m*/*z* 207; negative ionization) from oxidized MC-LR (**9**).

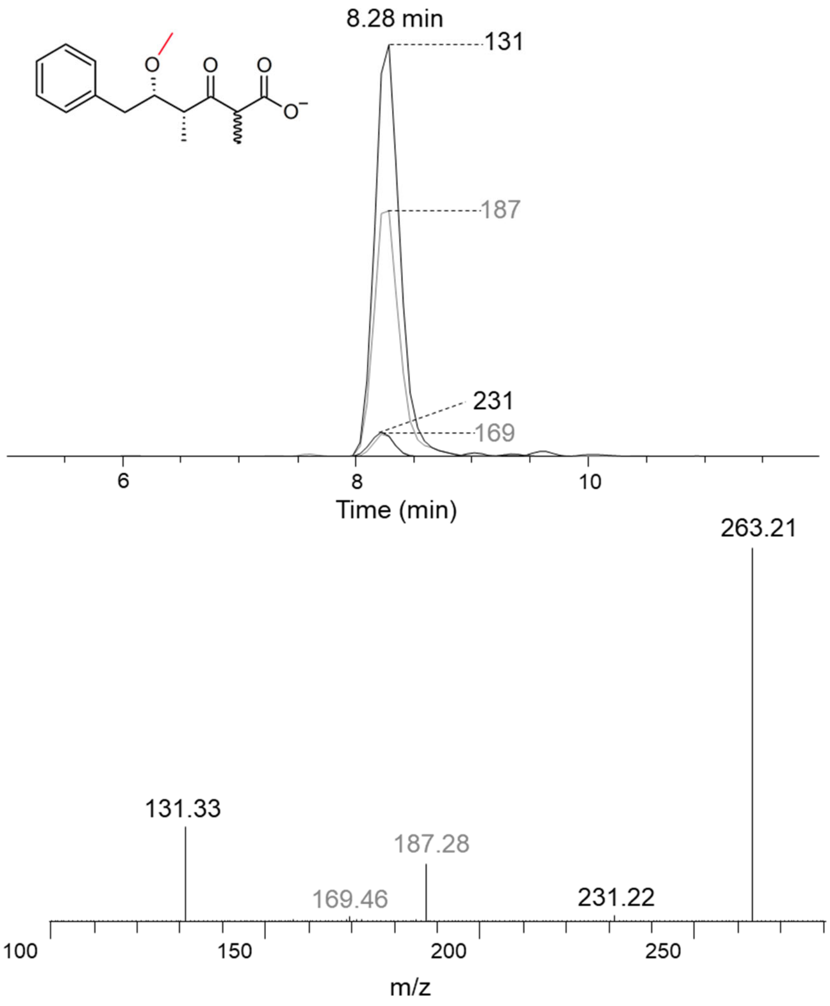

Figure S47: Triple-quadrupole LC–MS/MS chromatogram and spectrum of MOMMPH (*m*/*z* 263; negative ionization) from oxidized MC-LR (**9**).

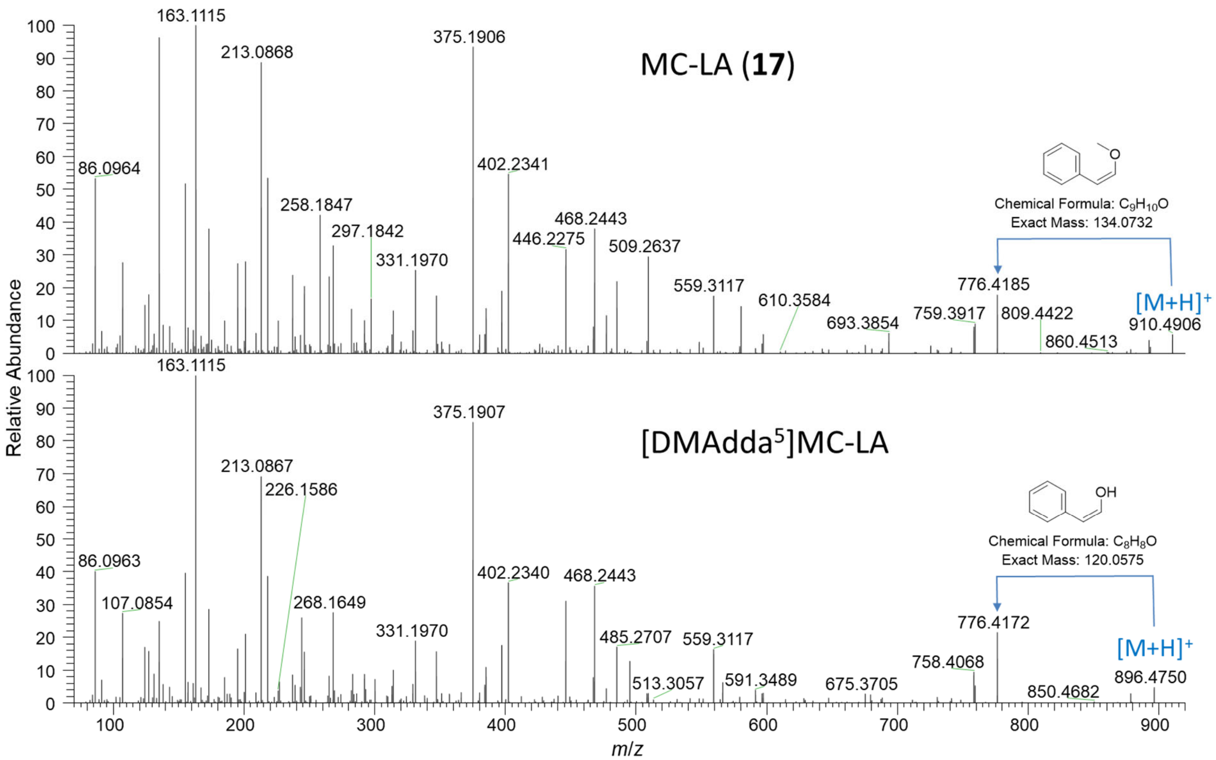

Figure S48: LC–HRMS/MS spectra obtained from an extract from the private lake in the mid-west of the USA, of MC-LA (**17**) (top, retention time 15.22 min) and [DMAdda^5]MC-LA (bottom, retention time 12.47 min), and displaying neutral loss of the Adda and DMAdda fragments (134.0732 and 120.0575 Da), respectively, to afford product ions at *m*/*z* 776.4189 in both spectra.
